# Hepatocyte specific expression of an oncogenic variant of β-catenin results in lethal metabolic dysfunction in mice

**DOI:** 10.18632/oncotarget.24346

**Published:** 2018-01-30

**Authors:** Ursula J. Lemberger, Claudia D. Fuchs, Christian Schöfer, Andrea Bileck, Christopher Gerner, Tatjana Stojakovic, Makoto M. Taketo, Michael Trauner, Gerda Egger, Christoph H. Österreicher

**Affiliations:** ^1^ Clinical Institute of Pathology, Medical University of Vienna, Vienna, Austria; ^2^ Hans Popper Laboratory for Molecular Hepatology, Department of Internal Medicine, Medical University of Vienna, Vienna, Austria; ^3^ Department of Cell and Developmental Biology, Medical University of Vienna, Vienna, Austria; ^4^ Department of Analytical Chemistry, Faculty of Chemistry, University of Vienna, Vienna, Austria; ^5^ Clinical Institute of Medical and Chemical Laboratory Diagnostics, Medical University of Graz, Graz, Austria; ^6^ Division of Experimental Therapeutics, Graduate School of Medicine, Kyoto University, Kyoto, Japan; ^7^ Ludwig Boltzmann Institute Applied Diagnostics, Vienna, Austria; ^8^ Institute of Pharmacology, Medical University Vienna, Vienna, Austria

**Keywords:** Wnt signaling pathway, liver cancer, lipid metabolism, glucose metabolism, mitochondrial disorder

## Abstract

**Background:**

Wnt/β-catenin signaling plays a crucial role in embryogenesis, tissue homeostasis, metabolism and malignant transformation of different organs including the liver. Continuous β-catenin signaling due to somatic mutations in exon 3 of the *Ctnnb1* gene is associated with different liver diseases including cancer and cholestasis.

**Results:**

Expression of a degradation resistant form of β-catenin in hepatocytes resulted in 100% mortality within 31 days after birth. *Ctnnb1*^*CA*^ ^*hep*^ mice were characterized by reduced body weight, significantly enlarged livers with hepatocellular fat accumulation around central veins and increased hepatic triglyceride content. Proteomics analysis using whole liver tissue revealed significant deregulation of proteins involved in fat, glucose and mitochondrial energy metabolism, which was also reflected in morphological anomalies of hepatocellular mitochondria. Key enzymes involved in transport and synthesis of fatty acids and cholesterol were significantly deregulated in livers of *Ctnnb1*^*CA*^ ^*hep*^ mice. Furthermore, carbohydrate metabolism was substantially disturbed in mutant mice.

**Conclusion:**

Continuous β-catenin signaling in hepatocytes results in premature death due to severe disturbances of liver associated metabolic pathways and mitochondrial dysfunction.

**Methods:**

To investigate the influence of permanent β-catenin signaling on liver biology we analyzed mice with hepatocyte specific expression of a dominant stable form of β-catenin (*Ctnnb1*^*CA*^ ^*hep*^) and their WT littermates by serum biochemistry, histology, electron microscopy, mRNA profiling and proteomic analysis of the liver.

## INTRODUCTION

The role of β-catenin has been extensively investigated, highlighting its importance for organogenesis, pattern formation, cell proliferation, tissue regeneration and apoptosis in different organs including the liver. Along these lines it has been shown that β-catenin is temporally active during liver development and is essential for hepatic morphogenesis and metabolic zonation [1, 2]. In adult liver β-catenin is rather inactive but remains an important regulator of liver homeostasis [3].

Hepatocyte specific deletion of β-catenin in mice (*Ctnnb1*^Δ*hep*^) results in underdeveloped livers characterized by immature hepatocytes, canalicular abnormalities, bile secretion defects and hence cholestasis and fibrosis [4–6]. Moreover, *Ctnnb1*^Δ*hep*^ mice display impaired liver regeneration after partial hepatectomy [7, 8]. Wnt/β-catenin signaling is responsible for the heterogeneity of hepatocytes, which results in a zonation with respect to metabolic processes. Periportal hepatocytes are predominantly involved in gluconeogenesis and β-oxidation, while hepatocytes located around central veins are responsible for glycolysis and lipogenesis. Close interaction between neighboring zones warrants quick and flexible adaption in response to different demands and ensures metabolic homeostasis [9, 10]. Disturbance of the Wnt signaling pathway affects the identity of hepatocytes resulting in perturbation of zonation and hence in metabolic liver disease [11–13].

Evidently, *Ctnnb1*^Δ*hep*^ mice are more susceptible to develop steatohepatitis in response to metabolic stress (MCD diet) and display aggravated steatohepatitis in response to alcohol intake [14, 15]. Finally, β-catenin is one of the most frequently mutated genes involved in malignant transformation and plays also a pivotal role in hepatic tumorigenesis [16]. Approximately 50% of patients suffering from hepatocellular carcinoma (HCC), up to 90% with hepatoblastoma (HB), and also patients with benign hepatoadenoma (HA) display mutations in the Wnt/β-catenin pathway [17, 18]. The vast majority of patients carry mutations located in exon 3 of the *CTNNB1* gene, resulting in a degradation resistant form of β-catenin [19]. These missense mutations in exon 3 of *CTNNB1* represent a subgroup of liver cancer with distinctive clinical and pathological features [20]. We recently presented a novel mouse model characterized by hepatocyte specific loss of exon 3 of *Ctnnb1 (Ctnnb1*^*CA hep*^ mice and *Ctnnb1*^*TCCA hep*^ mice). The resulting truncated version of β-catenin is fully functional but resistant to degradation. Using this model we demonstrated that continuous expression of β-catenin in hepatocytes induces cholestasis and a biliary type of fibrosis [21]. Following up on our previous study, here we analyzed the consequences of continuous β-catenin signaling on survival of *Ctnnb1*^*CA hep*^ mice and observed major metabolic defects leading to premature death and mitochondrial dysfunction.

## RESULTS

### Continuous β-catenin signaling in hepatocytes leads to premature death in mice

**Figure 1:**
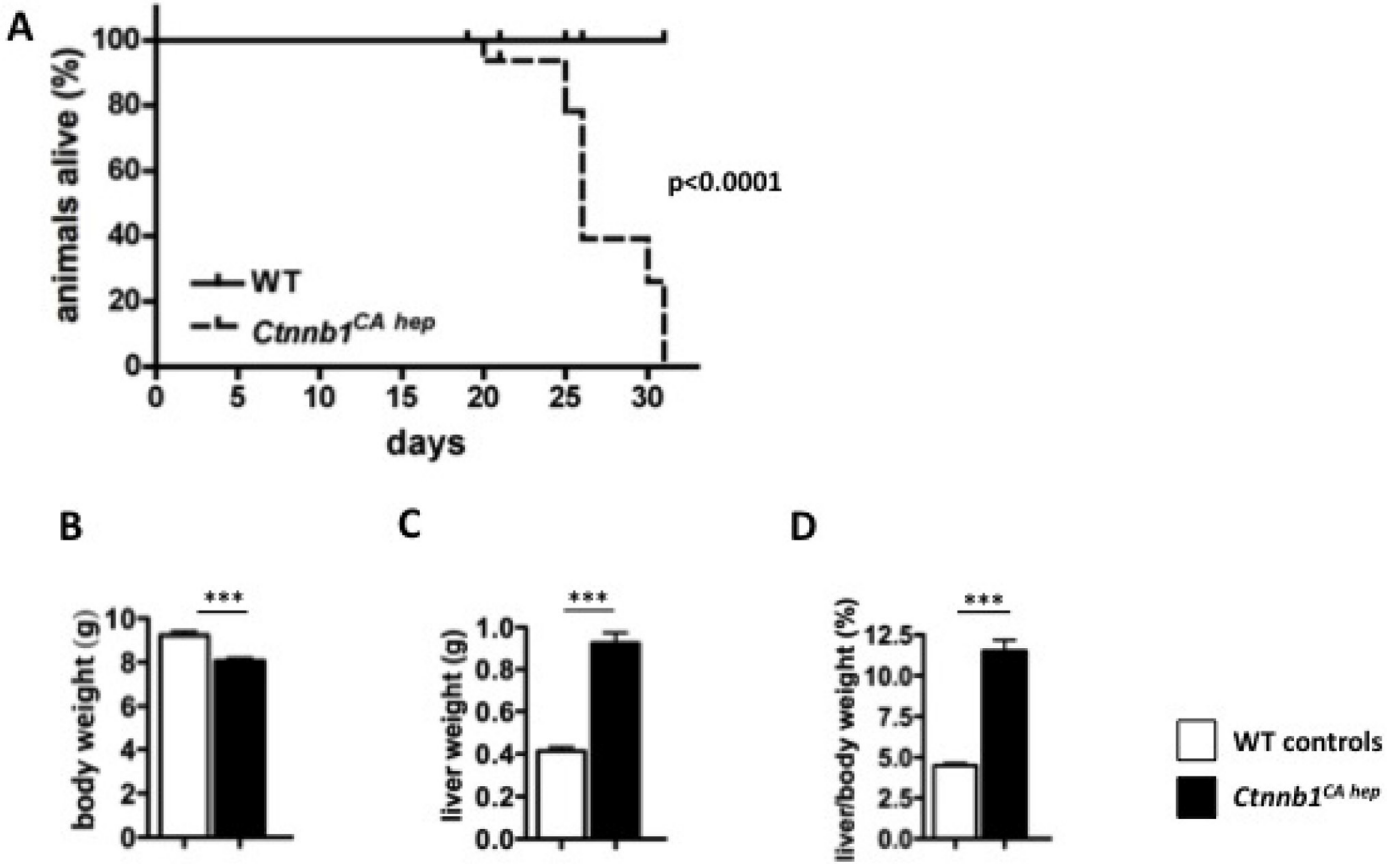
Continuous β-catenin signaling in hepatocytes results in 100% mortality and hepatomegaly All *Ctnnb1*^*CA hep*^ mice die within 31 days *post partum,* whereas WT animals display no mortality as shown by Kaplan Meier survival analysis (*n* = 15) (**A**). *Ctnnb1*^*CA hep*^ mice have significantly reduced body weight (**B**) but significantly enlarged livers (**C**) resulting in a highly increased liver/body weight ratio (**D**).

In our previous work we showed that *Ctnnb1*^*CA hep*^ mice, expressing a degradation resistant form of β-catenin, displayed severe deregulation of hepatic, renal and ileal transporters and disturbed liver architecture lacking lobule formation at weaning age [21]. To assess the long-term consequences of β-catenin expression we performed Kaplan Meier survival statistics (Figure 1A). All *Ctnnb1*^*CA hep*^ mice died within 31 days after birth with a median survival time of 25 days. *Ctnnb1*^*CA hep*^ mice were smaller and their body weight was significantly reduced compared to their Cre-negative littermates (8.05 g vs. 9.22 g; Figure 1B). Livers of *Ctnnb1*^*CA hep*^ mice were significantly enlarged (0.92 g vs. 0.41 g; Figure 1C) resulting in an increased liver to body weight ratio (11.5% vs. 4.5%; Figure 1D).

### Permanent active β-catenin leads to severe deregulation of protein homeostasis in livers

**Figure 2:**
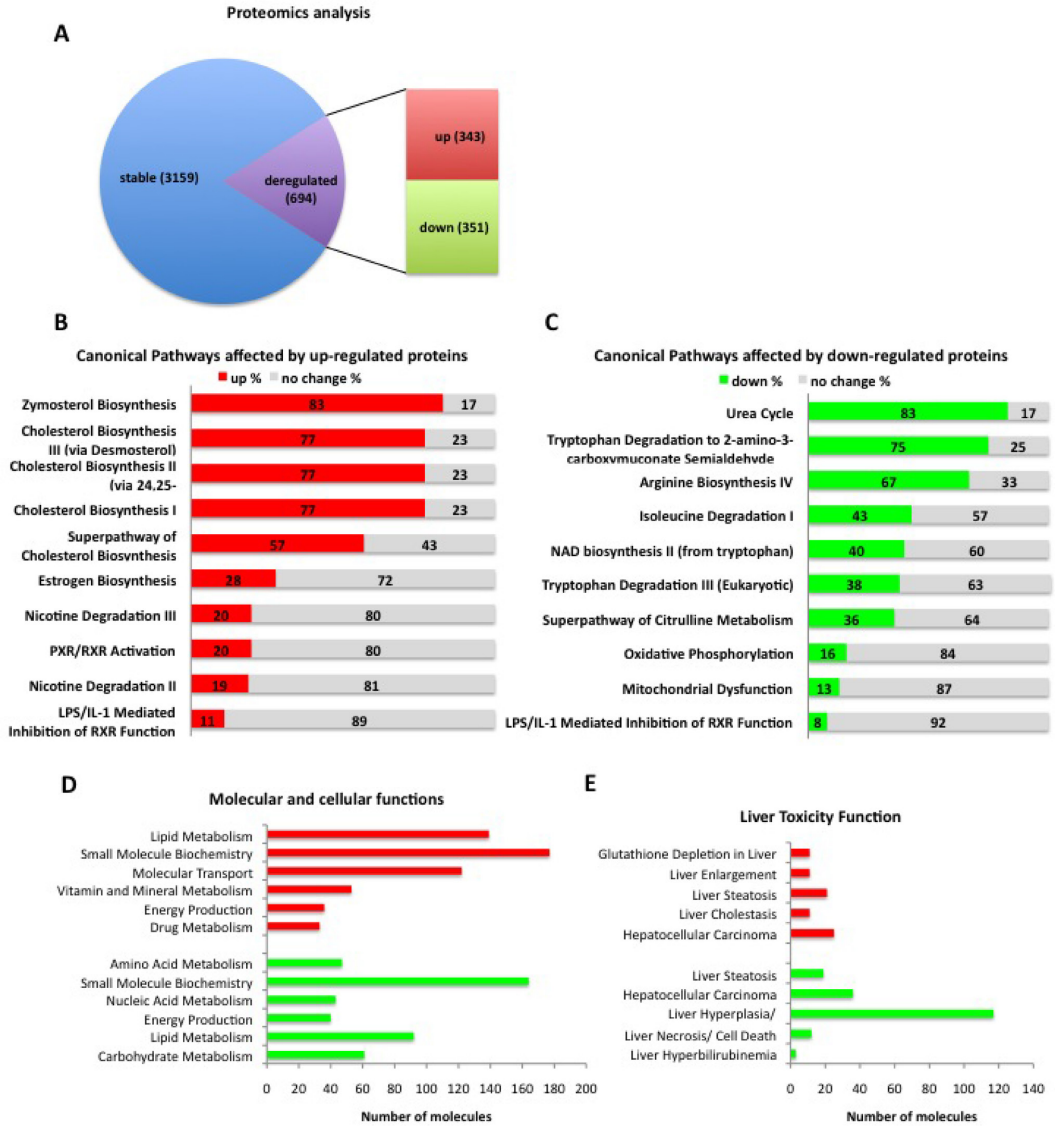
*Ctnnb1*^*CA hep*^ mice have severe disturbances in protein composition (**A**) Proteomics analysis identifies a total of 3863 proteins of which 3159 are expressed to a similar extend and 694 proteins are significantly deregulated in *Ctnnb1*^*CA hep*^ mice compared to WT littermates, (21.9%, *p*-value < 0.05; 0.58 log2 fold change in expression). 343 proteins are up-regulated and 351 proteins down-regulated. Ingenuity^®^ Pathway Analysis revealed top deregulated canonical pathways in *Ctnnb1*^*CA hep*^ mice compared to WT littermates (**B**, **C**). Deregulated proteins were analyzed according to their function and clustered into groups for molecular function (**D**) and liver toxicity (**E**).

In order to study the lethal phenotype of constitutive β-catenin signaling in hepatocytes in more detail, we performed whole liver proteomic analyses. Overall, 3853 individual proteins were identified by mass spectrometry analysis in livers of *Ctnnb1*^*CA hep*^ and control mice, of which 3159 were unaltered and 694 proteins (21.9%) were significantly deregulated in *Ctnnb1*^*CA hep*^ mice compared to WT littermates, respectively (*p*-value < 0.05; 0.58 log2 fold change in expression; Supplementary Table 1). From those, 343 proteins were up-regulated and 351 proteins were down-regulated in livers of *Ctnnb1*^*CA hep*^ mice compared to WT littermates. (Figure 2A).

In order to understand the biological relevance of the deregulated proteins we performed Ingenuity^®^ Pathway Analysis (IPA), which revealed that up-regulated proteins were involved in canonical pathways including the superpathway of cholesterol biosynthesis, PXR/RXR function and estrogen biosynthesis (Figure 2B). Analyzing the significantly down-regulated proteins, the top deregulated canonical pathways identified were mitochondrial dysfunction, oxidative phosphorylation, tryptophan degradation and the urea cycle (Figure 2C).

Moreover, up-regulated proteins were associated with molecular functions related to different metabolic processes (lipids, nucleic acids, amino acids) and energy production (Figure 2D). Analysis of down-regulated proteins identified proteins involved in metabolic functions (lipids, vitamins and minerals, drugs), for energy production and carbohydrate metabolism (Figure 2D). When analyzed for liver toxicity we identified a strong association of the deregulated proteins with liver steatosis, liver cholestasis and hyperplasia/carcinoma for up- and down regulated proteins, respectively (Figure 2E).

Together, these data suggest that aberrant hepatic β-catenin signaling largely affects proteins involved in essential metabolic and energy production pathways in the liver.

### Mice expressing constitutively active β-catenin in hepatocytes suffer from severe disturbances in lipid metabolism

**Figure 3:**
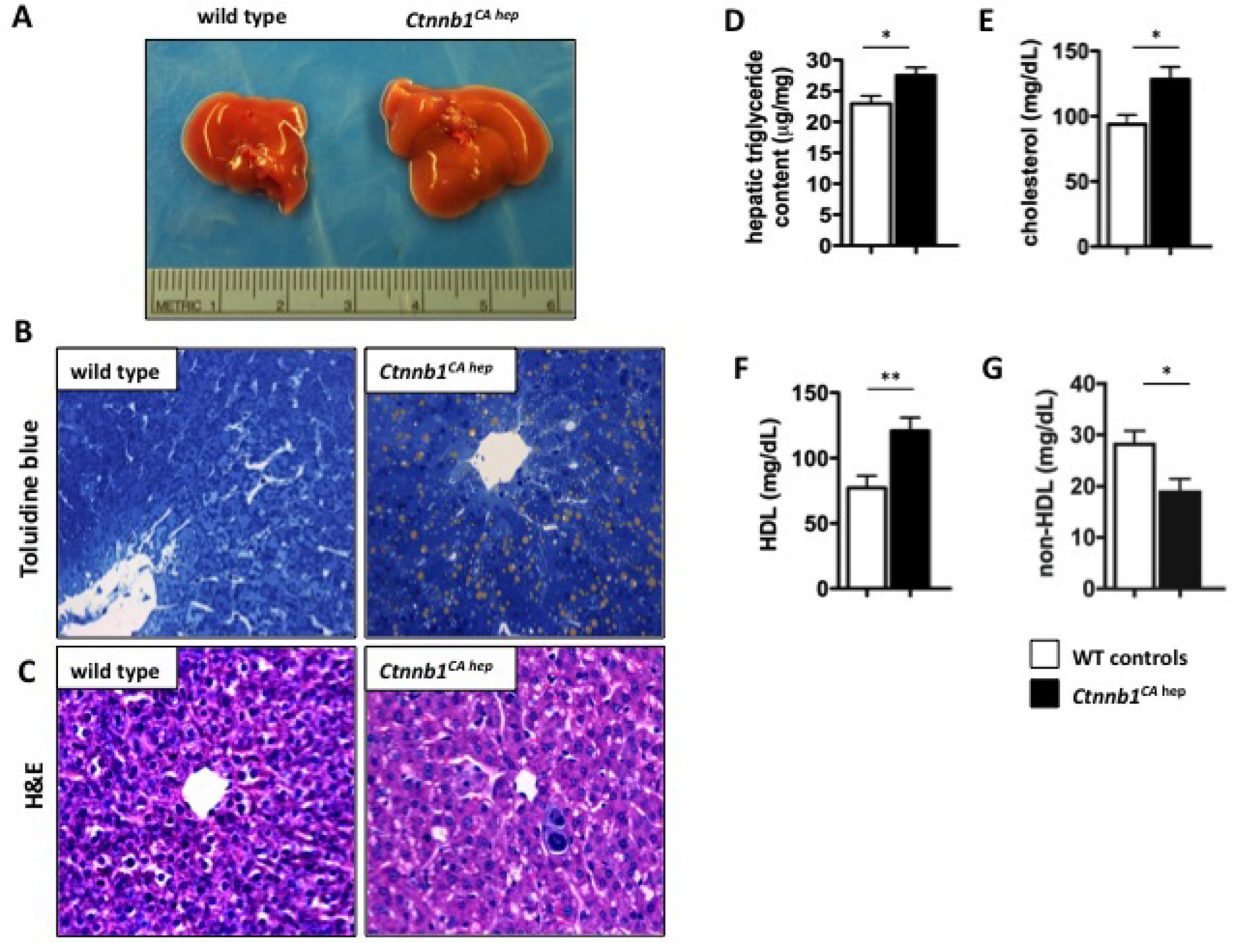
*Ctnnb1*^*CA hep*^ mice display hepatic steatosis Livers of *Ctnnb1*^*CA hep*^ mice are enlarged and show a yellowish appearance compared to livers of WT littermates (**A**). Livers of *Ctnnb1*^*CA hep*^ mice display hepatocellular fat accumulation around central veins confirmed by toluidine blue staining (**B**) and H and E staining (**C**). Livers are characterized by increased triglyceride content (**D**). Serum analysis showed significant increase of cholesterol and HDL cholesterol levels in *Ctnnb1*^*CA hep*^ mice (**E**, **F**), however there was a decrease in non-HDL cholesterol (**G**).

Macroscopically, livers of *Ctnnb1*^*CA hep*^ mice had a yellowish and swollen appearance indicating steatosis (Figure 3A). This was confirmed by toluidine blue staining, highlighting severe hepatocellular fat accumulation in *Ctnnb1*^*CA hep*^ mice (Figure 3B). Lipid droplets were predominantly observed in hepatocytes surrounding central veins (zone III of liver lobules) as revealed by H&E stained sections from livers of *Ctnnb1*^*CA hep*^ mice (Figure 3C). Further, livers of *Ctnnb1*^*CA hep*^ mice displayed increased triglyceride content (29.5 vs. 22.9 µg triglyceride/mg liver; Figure 3D). Serum cholesterol levels were also significantly elevated compared to WT animals (128.2 vs. 93.8 mg/dL; Figure 3E). Moreover, HDL levels were significantly increased in *Ctnnb1*^*CA hep*^ mice (120.8 vs. 77.1 mg/dL; Figure 3F), suggesting reverse cholesterol transport from peripheral white adipose tissue (WAT) to the liver. Interestingly, there was a decrease of non-HDL cholesterol levels in the serum of *Ctnnb1*^*CA hep*^ mice (18.8 vs. 28.2 mg/dL; Figure 3G). No changes with regards to free fatty acid (FA) levels were observed (Supplementary Figure 1A).

### Key enzymes involved in synthesis and transport of FA and cholesterol were significantly deregulated in livers of *Ctnnb1*^*CA hep*^ mice

**Figure 4:**
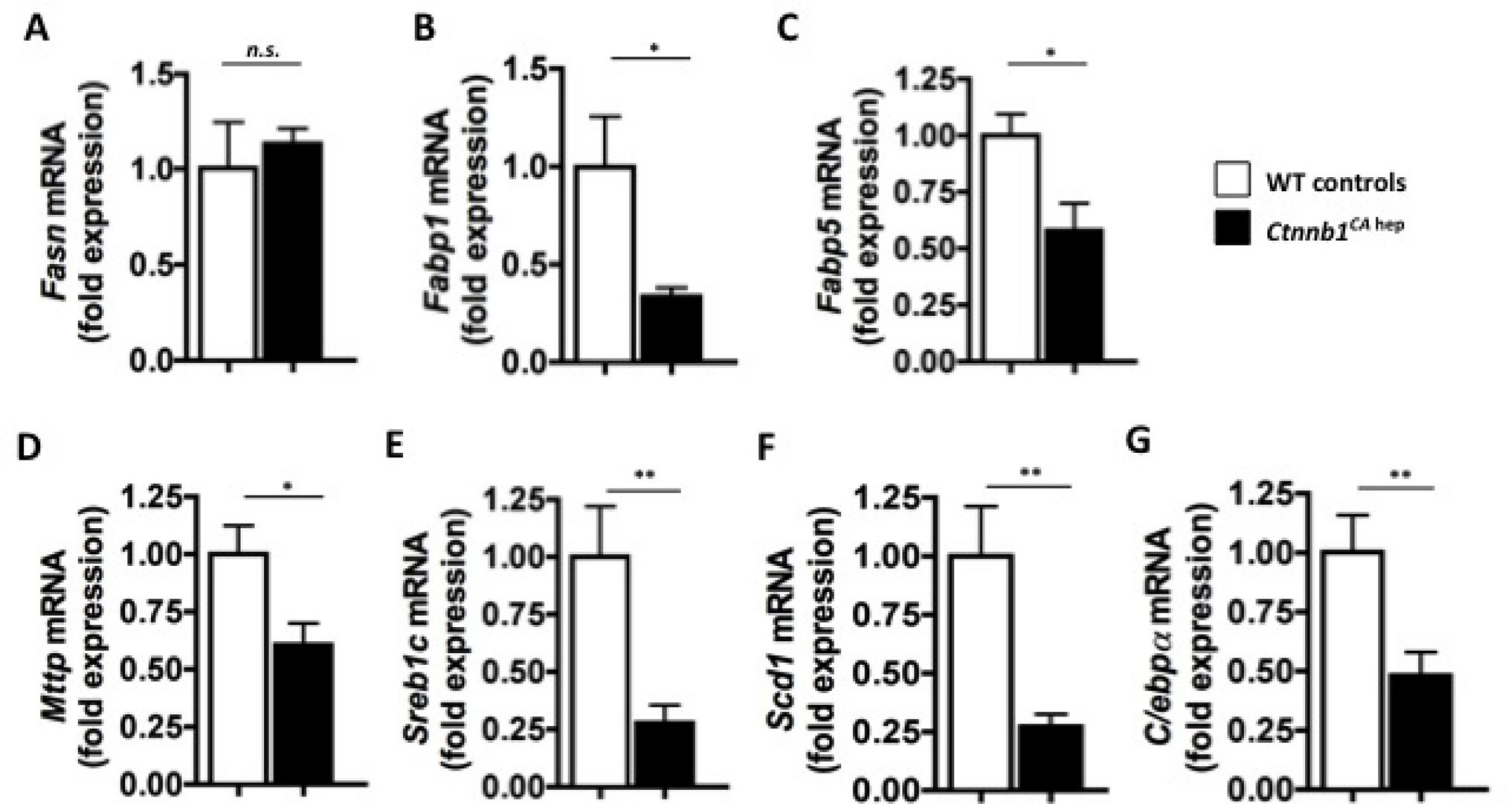
*Ctnnb1*^*CA hep*^ mice display deregulation of key enzymes of lipid metabolism Fatty acid synthase (*Fasn*; **A**), fatty acid binding proteins 1 (*Fabp1*; **B**) and 5 (*Fabp5*; **C**) microsomal triglyceride transfer protein large subunit (*Mttp*; **D**), sterol regulatory element binding protein 1c (*Sreb1c*; **E**) and stearoyl-CoA desaturase (*Scd1*; **F**) as well as adipogenic transcription factor CCAAT/enhancer binding protein alpha (C/ebpα, **G**) were down-regulated on mRNA level in *Ctnnb1*^*CA hep*^ mice.

To investigate the steatosis phenotype of *Ctnnb1*^*CA hep*^ mice in more detail we performed gene expression analysis of key genes involved in lipid metabolism. The mRNA expression of fatty acid synthase (*Fasn*; 1.1 vs. 1.0 fold expression; Figure 4A) was not significantly deregulated in *Ctnnb1*^*CA hep*^ mice compared to WT littermates. Interestingly, we detected significantly higher levels of FASN protein in *Ctnnb1*^*CA hep*^ mice based on our proteomics analyses, suggesting a posttranscriptional regulation of FASN. In contrast, mRNA levels of the main transporter for FA in the liver *Fabp1* (0.33 vs. 1.00 fold expression; Figure 4B) and the alternative transporter *Fabp5* (0.57 vs. 1.0 fold expression; Figure 4C) were significantly reduced. FABP5 was also significantly reduced on protein level (Supplementary Table 2A), while no significant changes were observed for FABP1 (Supplementary Table 1). These proteins facilitate the transport of lipophilic substances from outer cell membranes of hepatocytes to inner ones, and might be regulated by a negative feedback mechanism. These data suggest that a down-regulation of transporters may protect the liver from an overload of continuously provided lipids transported from the periphery in order to limit the aggravation of steatosis.

In line with these results, mRNA levels of microsomal triglyceride transfer protein large subunit, which plays a central role in lipoprotein assembly and hepatocellular excretion of lipids, were decreased in *Ctnnb1*^*CA hep*^ mice (*Mttp*; 0.6 vs. 1.0 fold expression; Figure 4D). Moreover, key regulators of *de novo* lipogenesis, sterol regulatory element-binding protein 1c (*Srebp*1c; 0.27 vs. 1.0 fold expression; Figure 4E) and stearoyl-CoA desaturase (*Scd1*; 0.27 vs. 1.0 fold expression; Figure 4F) were significantly reduced on mRNA levels, suggesting a compensatory reduction of lipid synthesis. These proteins were not detected in our proteomics analyses (Supplementary Table 1), however IPA analysis revealed an increase of proteins involved in the melanovate pathway. This pathway is responsible for syntheses of fatty acid from isoprenoid, which are further used in cholesterol generation and finally bile acid synthesis (Supplementary Table 2C).

Wnt/β-catenin signaling has been shown to maintain pre-adipocytes in an undifferentiated state through inhibition of the adipogenic transcription factors CCAAT/enhancer binding protein alpha (*C/ebp*α) and peroxisome proliferator-activated receptor gamma (*Pparγ*) [22]. In this respect, mRNA levels of *C/ebp*α were significantly reduced in livers of *Ctnnb1*^*CA hep*^ mice (0.48 vs. 1.0 fold expression; Figure 4G). No differences in mRNA levels of *Pparγ* and *Ppar*α between *Ctnnb1*^*CA hep*^ mice and littermate controls were observed (Supplementary Figure 1B, 1C). In proteomic analysis these proteins were not detected.

### Mice expressing constitutively active β-catenin in hepatocytes suffer from severe disturbances in glucose metabolism

**Figure 5:**
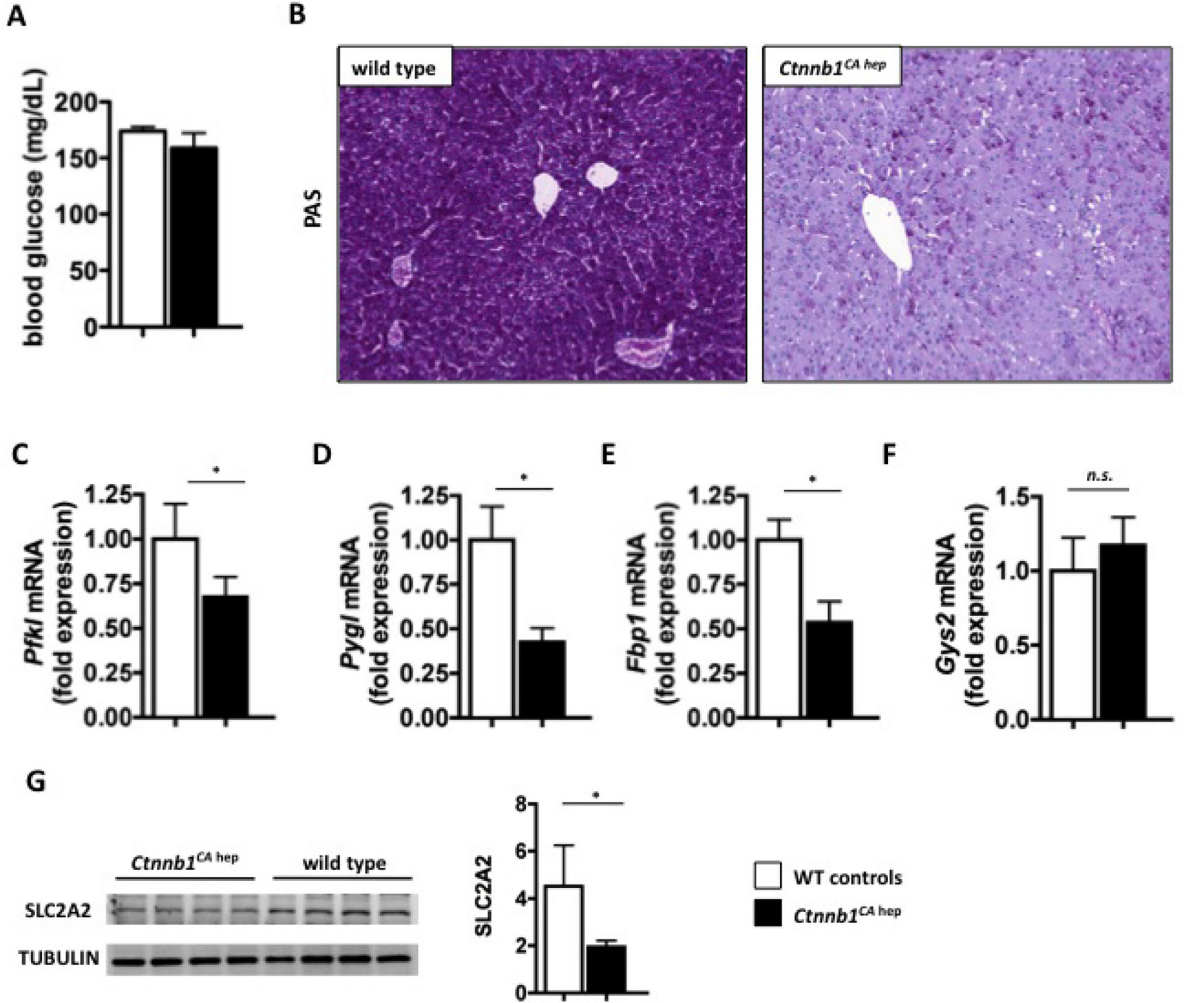
*Ctnnb1*^*CA hep*^ mice display severe disturbances in carbohydrate metabolism No difference in blood glucose levels between *Ctnnb1*^*CA hep*^ and WT littermates was detectable (**A**). PAS staining shows severely decreased levels of glycogen in *Ctnnb1*^*CA hep*^ mice (**B**). mRNA levels of key enzymes in glucose metabolism including Phosphofructokinase (Pfkl, **C**), liver specific glycogen phosphorylase (Pygl, **D**) and fructose 1,6-bisphosphatase (Fbp1, **E**) were significantly down-regulated in livers of Ctnnb1^CA hep^ mice. Glycogen synthase 2 (Gys2, **F**) was not deregulated on mRNA level. Western blot analysis revealed down-regulation of main glucose transporter GLUT2 (SLC2A2) in livers of *Ctnnb1*^*CA hep*^ mice compared to WT controls (**G**).

*Ctnnb1*^*CA hep*^ mice displayed rapid wasting, suggesting a deregulation of energy metabolism. Under baseline conditions blood glucose levels of *Ctnnb1*^*CA hep*^ mice did not differ from littermate controls (Figure 5A). However, hepatocytes of *Ctnnb1*^*CA hep*^ mice almost completely lacked glycogen storage as demonstrated by periodic acid-Schiff (PAS) staining (Figure 5B). In line with this observation, mRNA levels of liver-specific phosphofructokinase (*Pfkl*), the rate-limiting enzyme in glycolysis, was down-regulated (Figure 5C). Liver specific glycogen phosphorylase (*Pygl*) and fructose 1,6-bisphosphatase (*Fbp1*), key and rate-limiting enzymes in glycogen degradation and gluconeogenesis, respectively, were also significantly reduced in livers of *Ctnnb1*^*CA hep*^ mice (Figure 5D and 5E). Glycogen synthase (*Gys2*), the key enzyme in storage of glucose as glycogen, did not differ between *Ctnnb1*^*CA hep*^ mice and controls (Figure 5F). The deregulation of these key enzymes was also reflected in our proteomics data in addition to significant down-regulation of numerous enzymes involved in carbohydrate metabolism (Supplementary Table 2B).

Glucose and other carbohydrates are shuttled into the liver cells by the family of integral membrane glucose transporter (GLUT) molecules. The most abundant transporter in the liver is GLUT2 (solute carrier transporter family 2 member a2 SLC2A2), which was down-regulated on protein level as confirmed by Western blot (Figure 5G) and proteomics analysis in *Ctnnb1*^*CA hep*^ mice (Supplementary Table 1). Western blot analysis of two other abundant glucose transporters GLUT1 and SCL5A1 revealed no changes between the phenotypes (data not shown). Together, these data suggest that the deregulation of proteins involved in carbohydrate breakdown resulted from a deficiency of their substrate glucose and glycogen in livers of *Ctnnb1*^*CA hep*^ mice. Interestingly, liver proteomics revealed an up-regulation of alternative energy pathways. In *Ctnnb1*^*CA hep*^ mice enzymes involved in the Cahill cycle, which generates pyruvate from muscle-derived alanine, as well as the glutamine synthase cycle enzymes were highly up-regulated (Supplementary Table 2E). The increased use of muscle proteins for energy supply is associated with cachexia, reflected by the decreased body weight and feeble appearance of *Ctnnb1*^*CA hep*^ mice. Moreover, the key enzyme in lactate metabolism, L-lactate dehydrogenase subunit B (LDHB), which generates pyruvate from muscle derived lactate and was significantly up-regulated (Supplementary Table 2E). Increased lactate metabolism is a key mechanism of the cancer specific Warburg effect, which is characterized by glycolysis followed by lactate acid fermentation in the cytosol, rather than beta-oxidation in mitochondria. Another remarkable observation was the up-regulation of rate limiting enzymes of glycolysis (hexokinase 2, phosphofructokinase, and pyruvate kinase; Supplementary Table 2B). This feature has been observed in different tumors, like colon, prostate and also liver cancer and is also an indication for anaerobe glycolysis in cancer cells [23, 24].

### Continuous β-catenin signaling leads to massive mitochondrial damage

Proteomic analysis revealed a severe disturbance of mitochondrial protein homeostasis, and IPA pathways analysis predicted mitochondrial dysfunction based on deregulation of proteins associated with mitochondrial function (Figure 2C). A high number of enzymes involved in beta-oxidation and in the respiratory chain, the most important pathways to generate ATP and therefore energy, were significantly down-regulated in *Ctnnb1*^*CA hep*^ mice (Supplementary Table 2C). Additionally, proteins of the citrate-cycle, responsible for the energy-producing break down of metabolites from carbohydrate, lipid and protein metabolism, were also massively reduced (Supplementary Table 2C). Moreover livers of *Ctnnb1*^*CA hep*^ mice displayed deregulation of molecules involved in oxidative as well as ER stress responses. Glutathione reductase, glutathione S-transferase and paraoxonase 1 were up-regulated, while glutathione reductases were down-regulated (Supplementary Table 2C).

**Figure 6:**
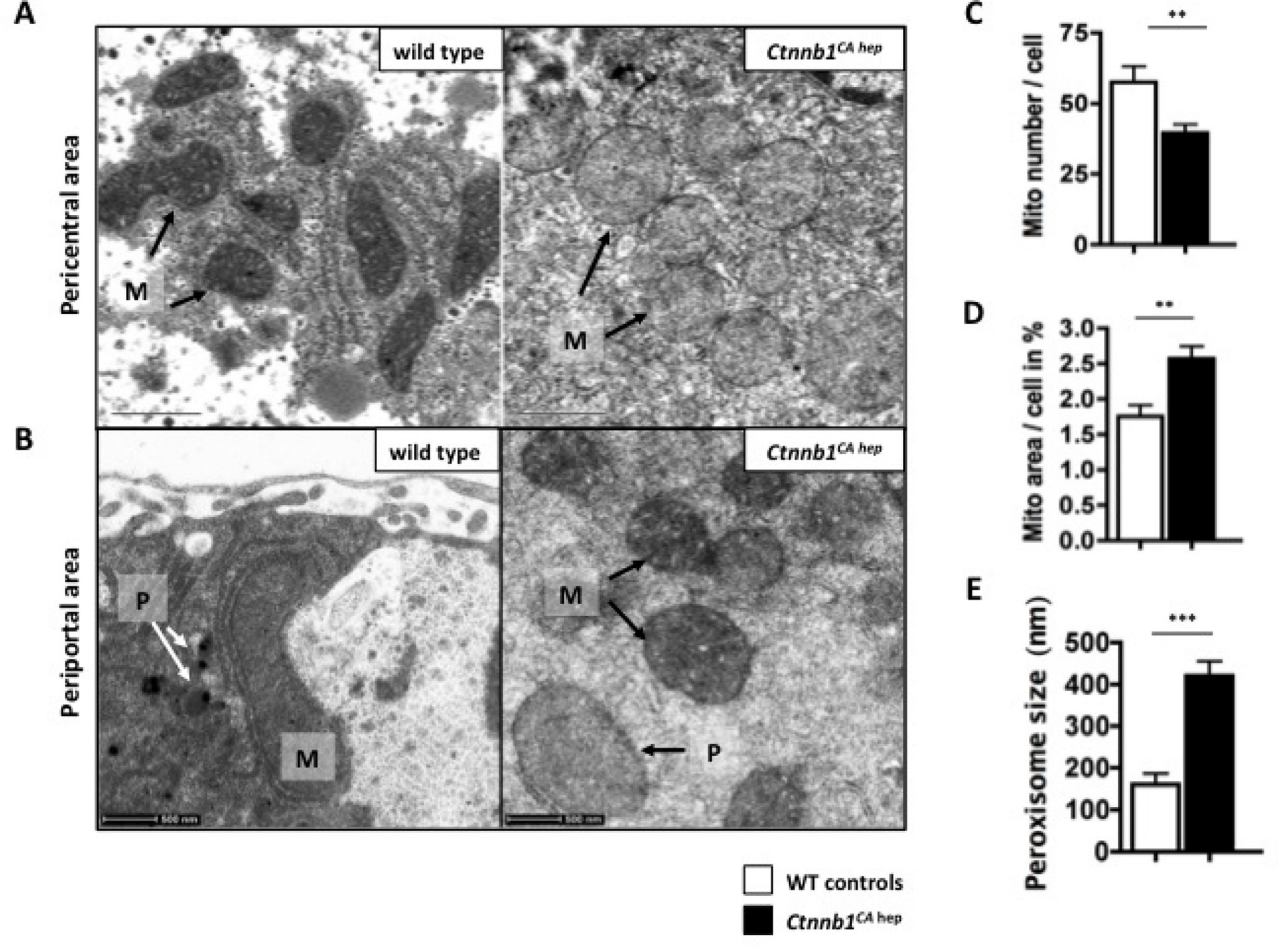
*Ctnnb1*^*CA hep*^ mice have a mitochondrial phenotype Mitochondria (M) of *Ctnnb1*^*CA hep*^ mice display a pathological phenotype shown by electron microscopic imaging. Especially around the central veins mitochondria are swollen with a rounded shape (**A**). Number of mitochondria per hepatocyte is decreased in *Ctnnb1*^*CA hep*^ mice (**B**), however they occupy a larger area in the cell compared to WT animals (**C**). Peroxisoma (**D**) of *Ctnnb1*^*CA hep*^ mice are highly enlarged and increased in number in *Ctnnb1*^*CA hep*^ mice (**B**, **E**).

Electron microscopy demonstrated that hepatic mitochondria of *Ctnnb1*^*CA hep*^ mice indeed had a pathologic phenotype (Figure 6A, 6B). They were swollen with rounded shape and displayed reduced matrix density compared to mitochondria of WT controls. This phenotype was more severe around central veins than in periportal areas. The number of mitochondria per hepatocyte was slightly decreased in *Ctnnb1*^*CA hep*^ mice compared to WT controls (Figure 6C). However the area occupied by mitochondria was larger in *Ctnnb1*^*CA hep*^ mice, which might be due to matrix swelling (Figure 6D).

Beside mitochondria, beta-oxidation can also take place in peroxisomes. Indeed, levels of peroxisomal proteins were highly increased in *Ctnnb1*^*CA hep*^ mice, indicating an increase of peroxisomal activity (Supplementary Table 2D). Moreover, electron microscopy revealed that peroxisomes of *Ctnnb1*^*CA hep*^ mice are highly enlarged but not increased in number (Figure 6B, 6E). Together, these data suggest that the massive deregulation of metabolic homeostasis in *Ctnnb1*^*CA hep*^ mice results in pathological alterations of mitochondria and peroxisomes.

### Mice expressing constitutively active β-catenin in hepatocytes do not tolerate fasting

**Figure 7:**
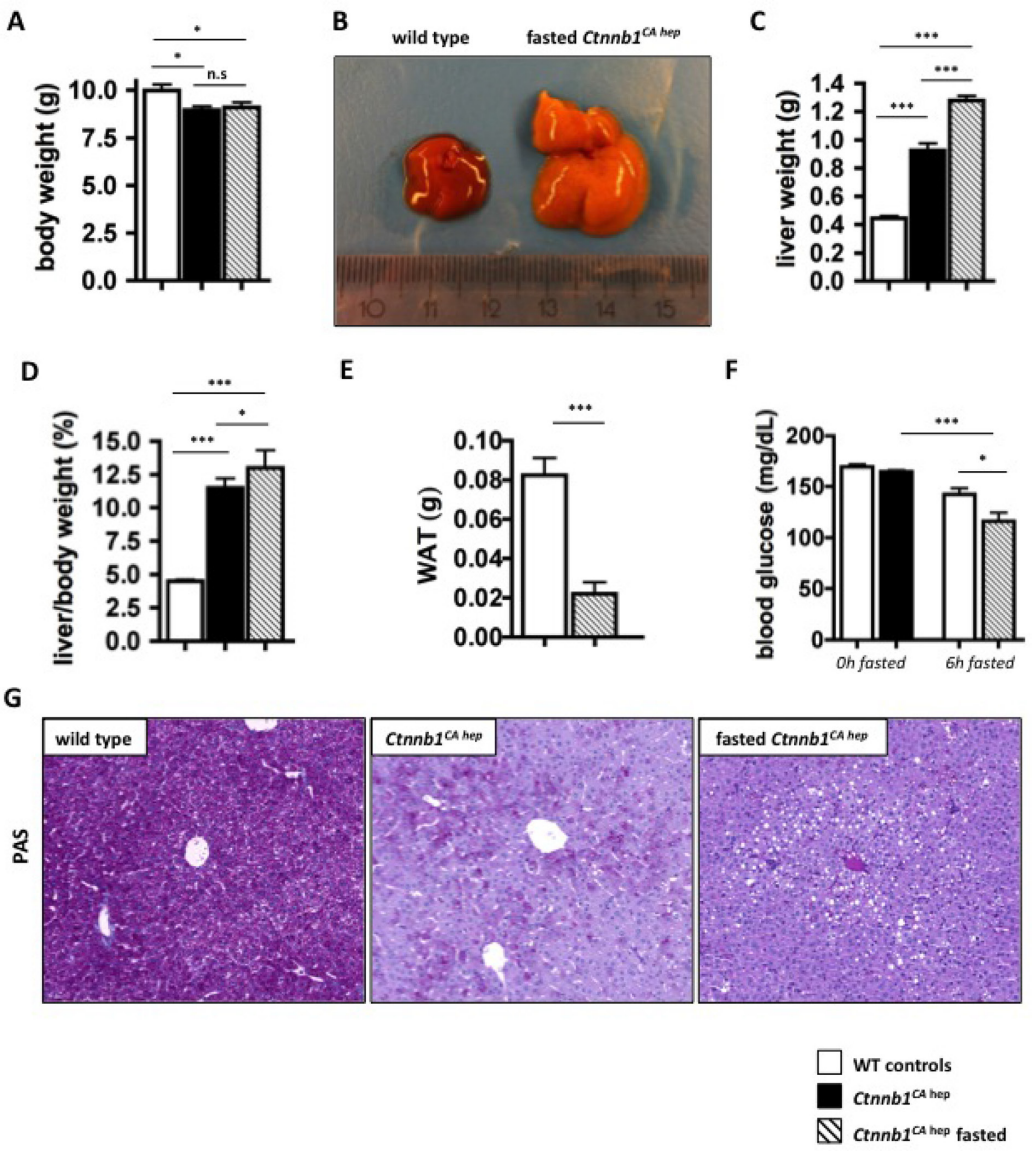
Fasting aggravates metabolic disturbances in *Ctnnb1*^*CA hep*^ mice After 6 hours of fasting *Ctnnb1*^*CA hep*^ mice showed no difference in body weight compared to normal fed *Ctnnb1*^*CA hep*^ mice (**A**) but significantly enlarged livers (**B**, **C**) resulting in a significantly increased liver/body weight ratio (**D**). Fasted *Ctnnb1*^*CA hep*^ showed reduced white adipose fat (WAT; **E**), reduced blood glucose levels (**F**) and the inability to form glycogen displayed by PAS staining (**G**).

**Figure 8:**
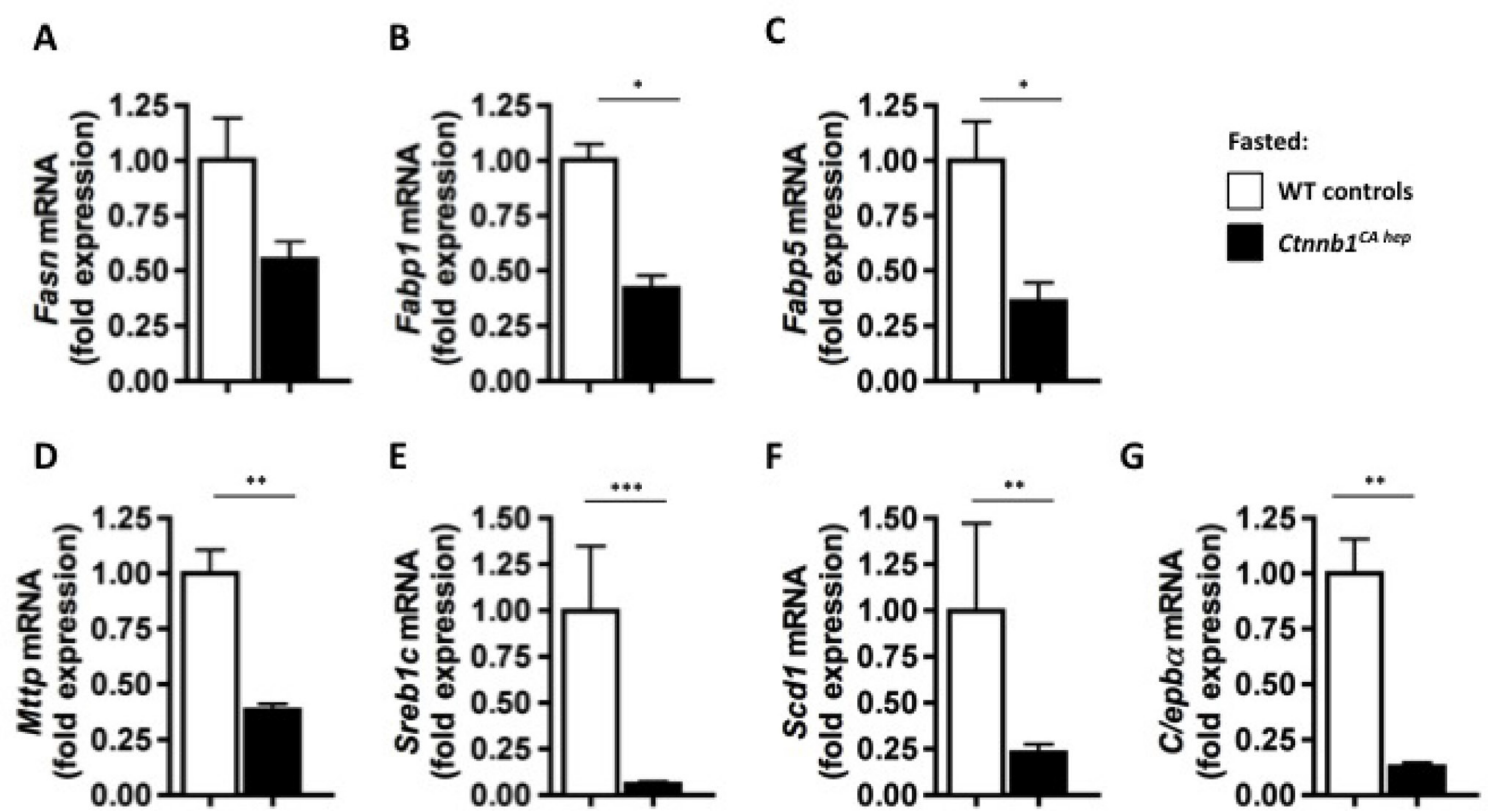
Fasted *Ctnnb1*^*CA hep*^ mice display aggravated down regulation of key enzymes of lipid metabolism Fatty acid synthase (*Fasn*; **A**), fatty acid binding proteins 1 (*Fabp1*; **B**) and 5 (*Fabp5*; **C**) microsomal triglyceride transfer protein large subunit (*Mttp*; **D**), sterol regulatory element binding protein 1c (*Sreb1c*; **E**) and stearoyl-CoA desaturase (*Scd1*; **F**) as well as adipogenic transcription factor CCAAT/enhancer binding protein alpha (C/ebpα, **G**) were down-regulated on mRNA level in fasted *Ctnnb1*^*CA hep*^ mice compared to their healthy littermates.

To further investigate the metabolic conditions of *Ctnnb1*^*CA hep*^ mice we performed fasting experiments. After weaning at the age of 21 days, food was withdrawn from *Ctnnb1*^*CA hep*^ mice and wild type controls. After 6 hours, the experiment had to be terminated as *Ctnnb1*^*CA hep*^ mice became lethargic and showed a weak constitution. After the fasting period, *Ctnnb1*^*CA hep*^ mice did not show a change in overall body weight (Figure 7A). However, livers of fasted *Ctnnb1*^*CA hep*^ mice appeared yellowish and spongy (Figure 7B) and were significantly heavier compared to un-fasted *Ctnnb1*^*CA hep*^ mice and WT littermates (1.29 vs. 0.92 vs. 0.44 g, Figure 7C) resulting in an even more pronounced liver to body weight ratio than in fed animals (13.0 vs. 11.5 vs. 4.5, Figure 7D). The visceral fat pad was significantly reduced in *Ctnnb1*^*CA hep*^ mice (0.02 vs. 0.08 g, Figure 7E). After 6 hours of fasting *Ctnnb1*^*CA hep*^ mice displayed significantly reduced serum glucose levels compared to fed *Ctnnb1*^*CA hep*^ mice and their WT littermates (116.2 vs. 142.2 vs. 164.6 mg/dL, Figure 7F). PAS staining revealed a complete lack of glycogen and additionally highlighted the severe hepatocellular steatosis around central veins in fasted *Ctnnb1*^*CA hep*^ mice (Figure 7G). Despite the accelerated hepatic lipid accumulation of fasted *Ctnnb1*^*CA hep*^ mice compared to unfasted mice, they displayed a reduction of *Fasn* (0.55 vs. 1.00 fold expression, Figure 8A), *Fabp1* (0.42 vs. 1.03 fold expression, Figure 8B), *Fabp5* (0.36 vs. 1.00 fold expression, Figure 8C). However the mRNA levels of *Mttp* (0.38 vs. 1.0 fold expression; Figure 8D), *Srebp*1c (0.05 vs. 0.99 fold expression; Figure 8E), *Scd1* (0.23 vs. 0.99 fold expression; Figure 8F) and *C/ebp*α (0.12 vs. 1.0 fold expression; Figure 8G) were even stronger reduced in fasted compared to unfasted *Ctnnb1*^*CA hep*^ mice compared to their wild type controls.

### The metabolic phenotype is inducible in *Ctnnb1*^*TCCA hep*^ mice

**Figure 9:**
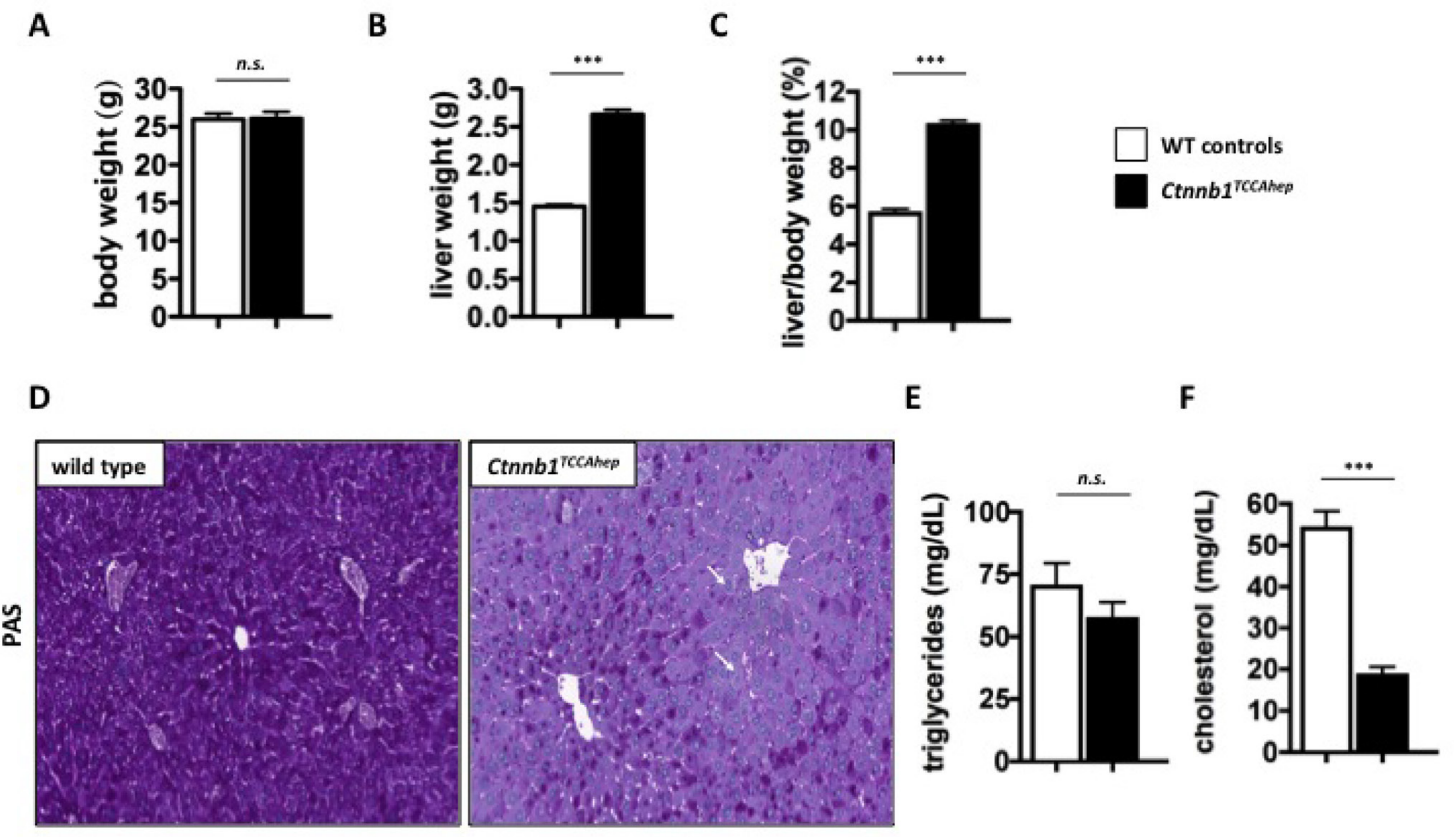
*Ctnnb1*^*TCCA hep*^ mice display same metabolic deregulations as *Ctnnb1*^*CA hep*^ mice 10-week-old *Ctnnb1*^*TCCA hep*^ mice display normal body weight (**A**) but significantly enlarged livers (**B**) resulting in a highly increased liver/body weight ratio (**C**) after induction of continuous β-catenin signaling. They also display lack of glycogen storage in the liver indicated by PAS staining, lipid accumulation in hepatocytes is indicated by white arrows (**D**). Serum triglycerides were not changed (**E**), however serum cholesterol levels were significantly reduced (**F**).

To investigate if the deregulation of metabolic pathways results from a developmental defect of Ctnnb1 deletion, we also investigated inducible *Ctnnb1*^*TCCA hep*^ mice, where continuous β-catenin signaling is induced by tamoxifen injection. Ten-week-old *Ctnnb1*^*TCCA hep*^ mice and their healthy littermates were treated with tamoxifen as described before and analyzed 14 days after the first administration [25]. *Ctnnb1*^*TCCA hep*^ mice displayed normal body weight (26.0 g vs. 25.9 g; Figure 9A) compared to their healthy littermates, however their livers were highly enlarged (2.6 g vs. 1.4 g; Figure 9B) resulting in an impaired liver to body ratio (10.2 % vs 5.6 %; Figure 9C).

Similar to *Ctnnb1*^*CA hep*^ mice, *Ctnnb1*^*TCCA hep*^ mice also displayed a severe lack of glycogen, especially around portal fields as indicated by PAS staining (Figure 9D). In addition we also observed a mild steatosis around portal fields (Figure 9D), although no significant changes in hepatic triglycerides were detected (57.0 vs 70.0 µg triglyceride/mg liver; Figure 9E). Serum analysis revealed significantly reduced blood cholesterol levels (18.5 vs. 54.0 mg/dL; Figure 9F).

**Figure 10:**
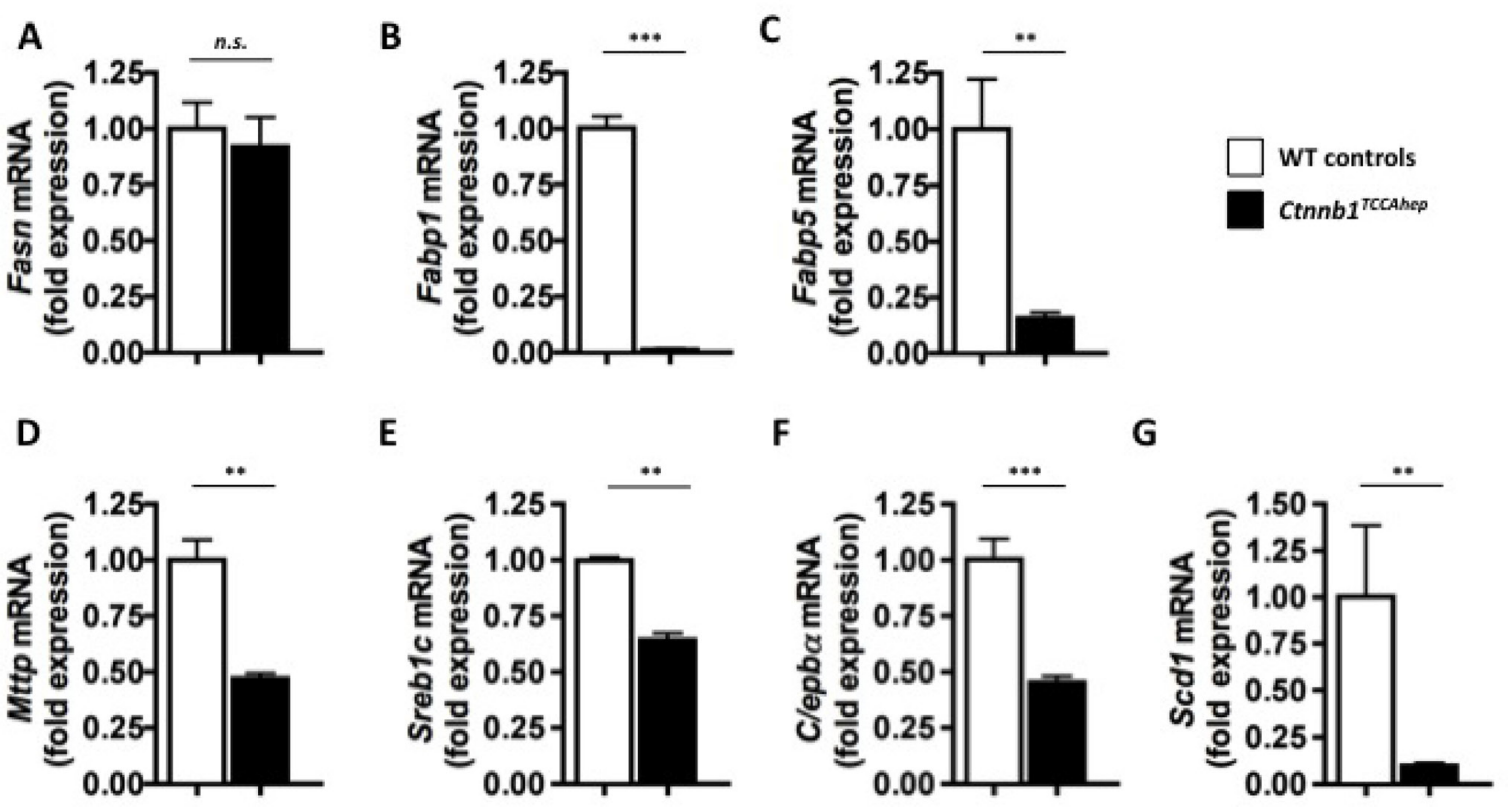
*Ctnnb1*^*TCCA hep*^ mice display deregulation of key enzymes of lipid metabolism *Fasn*; (**A**), *Fabp1* (**B**), *Fabp5* (**C**), *Mttp* (**D**), *Sreb1c* (**E**) , *Scd1* (**F**) as well as C/ebpα (**G**) were down-regulated on mRNA level in *Ctnnb1*^*TCCA hep*^ mice.

Moreover, qPCR analysis of key enzymes involved in lipid metabolism displayed a similarly decreased mRNA expression pattern as observed in the conditional *Ctnnb1*^*CA hep*^ mice: *Fasn* (0.92 vs. 1.00 fold expression, Figure 10A), *Fabp1* (0.01 vs. 1.02 fold expression, Figure 10B), *Fabp5* (0.15 vs. 1.00 fold expression, Figure 10C), *Mttp* (0.47 vs. 1.0 fold expression; Figure 10D), *Srebp*1c (0.64 vs. 0.99 fold expression; Figure 10E), *Scd1* (0.09 vs. 1.00 fold expression; Figure 10F) and *C/ebp*α (0.45 vs. 1.00 fold expression; Figure 10G).

This data indicate that the early lethal phenotype observed in conditional *Ctnnb1*^*CA hep*^ mice is not due to perturbation of hepatic development, but rather can be induced in adult mice by constitutional expression of ß-catenin, highlighting the vital role of β-catenin in liver homeostasis and function.

## DISCUSSION

Approximately 50% of patients with HCC display mutations in the Wnt/β-catenin signaling pathway [20]. Recently, it was demonstrated that HCCs harboring somatic missense mutations in exon 3 of *CTNNB1* represent a subgroup of liver cancer with distinctive clinical and pathological features [26, 27]. Here we show that hepatocyte specific expression of exon 3 mutated *Ctnnb1* in mice (*Ctnnb1*^*CA hep*^) resulted in a lethal phenotype with a median survival of 25 days after birth. At this time point not a single mouse had developed a lesion or tumor nodule. However, *Ctnnb1*^*CA hep*^ mice displayed a severe phenotype characterized by disturbed liver architecture, deregulation of transporters and bile acids as well as cholestasis and biliary type of fibrosis [25]. The most impressive symptom was the rapid wasting of *Ctnnb1*^*CA hep*^ mice from day 14 until their premature death, suggesting that continuous β-catenin signaling had a severe impact on metabolism and energy balance. The liver is the central organ of glucose, lipid and protein metabolism and responsible for energy supply. Comparing the phenotype of *Ctnnb1*^*CA hep*^ mice to human liver disease we find parallels, especially in patients with metabolic liver disorders resulting in HCC or HB. A recent study described two distinct metabolic phenotypes of fetal and embryonic liver cancer. Fetal HB is characterized by large deletions in *CTNNB1*, which encompass exon 3 and parts of exon 4, whereas embryonal HB more frequently display point mutations in the exon3 region of *CTNNB1* [27]. Our data partially overlap with both phenotypes, however there is more similarity to the fetal subtype of HB, especially concerning the glucose deficiencies and increased hexokinase 2 expression. Moreover, there are different metabolic disease like Glycogen storage disease (GSD), a rare disease characterized by defects in different key enzymes involved in glucose/glycogen metabolism. Patients suffer from deregulation of lipid metabolism and fatty liver steatosis [28]. The liver cancer incidence in these patients is high, although the pathogenesis of tumor formation is not understood [29]. However, 28% of all GSD patients diagnosed with hepatocellular adenoma display mutations in *CTNNB1*, which accelerate the metabolic phenotype and the malignant transformation towards HCC [30]. Also in *Ctnnb1*^*CA hep*^ mice one of the fundamental metabolic problems is associated with lack of glucose/glycogen in the liver; the reasons therefore could be numerous. In our previous work we showed significant reduction of *Fxr* expression on mRNA as well as on protein level in *Ctnnb1*^*CA hep*^ mice. It has been demonstrated that *Fxr* KO mice also suffer from severe disturbances in glucose metabolism due to the development of insulin resistance [31]. However, we can exclude insulin-resistance in *Ctnnb1*^*CA hep*^ mice since we did not observe differences in blood glucose concentration of either genotype, although enzymes involved in gluconeogenesis were also down-regulated.

In general continuous expression of the truncated form of β-catenin could lead to an over expression of target genes but also to a loss of interaction with specific co-activators of Wnt-target genes resulting in down regulation of target genes.

Most of the key enzymes responsible for glycolysis, glycogenesis and glycogenolysis were down-regulated on mRNA as well as on protein level suggesting a physiological regulation of enzymes due to the lack of their substrates glucose and glycogen. However, we cannot exclude down regulation due to defects on transcriptional level. Consequently, other substrates and pathways are needed to generate ATP.

An alternative way to maintain energy supply is the oxidation of free fatty acids in the mitochondria via beta-oxidation and citric acid cycle and finally the respiratory chain. In *Ctnnb1*^*CA hep*^ mice, especially in fasted animals, the increased fatty acid mobilization from the peripheral WAT seems to be an energetic rescue mechanism in order to perform beta-oxidation. According to the high intake and production of lipids, liver steatosis is observed in *Ctnnb1*^*CA hep*^ mice, representing a typical feature of hepatic dysfunction. The decrease of key regulators of fatty acid transport on mRNA level seems to be a physiological response to prevent further accumulation of lipids in the liver. The release of lipids into the blood compartment combined with a decreased lipid clearance from the blood leads to hypercholesterolaemia and hypertriglyceridaemia similar to a human subgroup of patients suffering liver disease [32, 33]. In the liver two possible clearance strategies are observed; increase in bile acid synthesis to metabolize cholesterol and/or increased beta-oxidation, respectively. Indeed, *Ctnnb1*^*CA hep*^ mice display increased levels of enzymes involved in the melanovate pathway, which is responsible for isoprenoid synthesis in order to generate cholesterol and steroids for bile acid synthesis. These findings suggest that the observed increase in bile acid synthesis, described in our previous work, is a rescue mechanism to clear the liver from lipids in order to prevent liver steatosis.

Beside mitochondria, also peroxisomes can perform beta-oxidation. Peroxisome activation and especially beta-oxidation comes in effect in conditions of substrate overload, facilitating clearance of especially long chain fatty acids. We therefore assume that the observed peroxisome phenotype is a reaction to the steatosis.

However, peroxisome beta-oxidation harbors the jeopardy of generating high levels of reactive oxygen species (ROS) and unlike mitochondria also hydrogen peroxide, which is converted to highly reactive hydroxyl radicals. Evidentially, significantly deregulated levels of enzymes involved in oxidative stress reaction were detected. These enzymes catalyze detoxification of lipid-peroxides and ROS and are an indicator of oxidative injury of mitochondria. Moreover, levels of extracellular superoxide dismutase, another key mediator preventing oxidative damage were significantly down-regulated in *Ctnnb1*^*CA hep*^ mice [34].

Finally, we suggest that the high levels of ROS and other peroxisomal derived radicals combined with the deregulation of oxidative stress response destroy mitochondria, especially around the central veins, where FA supply is the highest. Moreover, it has been shown that high activation of proto-oncogenes is able to induce down-regulation of mitochondrial enzymes, which might be an additional problem in mice with continuous expression of β-catenin [35]. Interestingly, hepatocyte specific ablation of β-catenin also results in down-regulation of mitochondrial enzymes, highlighting its important role in maintenance of mitochondrial homeostasis [36].

Investigation of different mouse models overexpressing β-catenin display significant differences concerning the targeted effector molecule. *Ctnnb1*^*CA hep*^ mice have a similar phenotype compared to hepatocyte specific *Apc* KO mice, which are also characterized by continuous β-catenin signaling. *Apc* KO mice also display an up-regulation of bile acid/drug metabolism and glutamine synthesis pathway combined with a down- regulation of glucose, lipid and amino acid catabolism and an energetic shift towards anaerobic glycolysis [13, 37]. In fact the top 5 up/down-regulated proteins in our liver proteomics analysis are the same as observed in *Apc* KO mice. Although the phenotype of *Apc* KO mice was similar, these mice displayed increased glucose uptake demonstrated via a glucose analogue tracer 2-[18F]-FDG [38]. Therefore truncated β-catenin might directly interfere with proteins involved in glucose metabolism.

In summary, our study provides further evidence linking β-catenin signaling to metabolic deregulation and down-regulation of genes involved in energy-producing processes as observed in a subgroup of liver cancer patients, especially in the pediatric subtype of liver cancer.

However, further studies are necessary to understand the impact and complexity of continuous β-catenin signaling in hepatocytes.

## MATERIALS AND METHODS

### Mice

*Ctnnb1*^*CA hep*^ and *Ctnnb1*^*TCCAhep*^ mice were generated as described before [21]. All *Ctnnb1*^*CA hep*^ mice were sacrificed at the age of 21 days. *Ctnnb1*^*TCCAhep*^ mice were injected with tamoxifen at the age of 10 weeks and sacrificed 12 days afterwards.

Cre-negative littermates were exclusively used as controls throughout the entire study. All experiments performed in this study were approved by the local ethical committee (BMWFW-66.009/0134-WF/V/3b/2015).

### Serum parameters

Serum parameters were quantified using standard methods as described previously [39].

### Histology

Liver tissue was processed as described previously [21]. Toluidine blue staining, H&E and Periodic acid-Schiff (PAS) staining were performed using standard protocols. Detailed protocols for each staining will be provided upon request.

### Quantitative real-time PCR

Total cellular RNA was isolated using TRI^®^ Reagent RNA Isolation Reagent (Sigma Aldrich) from snap frozen liver tissue following the instructions of the manufacturer. Complementary DNA (cDNA) was generated from 2 µg of total RNA using the High Capacity cDNA Reverse Transcription Kit (Applied Biosystems) following the instructions of the manufacturer. iQ™ SYBR^®^ Green Supermix (BioRad) was used for quantitative PCR (qPCR). Primer sequences were obtained from the qPrimerDepot (http://mouseprimerdepot.nci.nih.gov) and will be provided upon request. Melting curve analysis and agarose gel electrophoresis was performed to assess the quality of primers and the qPCR.

### Proteomics

For whole liver proteomic analysis 3 *Ctnnb1*^*CA hep*^ mice and 3 WT littermates were harvested at the age of 20 days and livers were snap frozen in liquid nitrogen. 2 mg of tissue were homogenized with an ultrasonic homogenizer in Sample Buffer (Urea, Thiourea, CHAPS, SDS, DTT). Then the solution was digested with trypsin.

Proteins were detected by LC (UltiMateTM 3000 RSLCnano) - MS/MS (QExactive Orbitrap) as described before [40]. Obtained data were evaluated by *T*-test (t2 test to consider the variance between groups (difference of mean) but also the variance within groups. Values of *p* < 0.05 and 0.58 log2 fold change were considered significant.

### Data analysis

Proteomic data was analyzed using Ingenuity^®^ Pathway Analysis (IPA; http://www.ingenuity.com).

## SUPPLEMENTARY MATERIALS FIGURE AND TABLES







## Abbreviations

AOX: Fatty acyl-coenzyme oxidase
CPT1: Carnitine palmitoyltransferase
CTNNB1: β-catenin (cadherin-associated protein) beta 1
FA: Fatty acid
FABP: Fatty acid binding protein
FASN: Fatty acid synthase
FBP1: Fructose 1,6-bisphosphatase
GLUT2: Glucose transporter 2
GSK-3β: glycogen synthase kinase-3β
GYS2: Glycogen synthase
H and E: hematoxylin and eosin
HB: Hepatoblastoma
HCC: Hepatocellular carcinoma
HDL: High-density lipoprotein
LDL: Low-density lipoprotein
MTTP: Microsomal triglyceride transfer protein
PFKL: Liver-specific phosphofructokinase
PPARα: Peroxisome proliferator-activated receptor alpha
PYGL: Liver specific glycogen phosphorylase
SCD1: Stearoyl CoA desaturase 1
SREBP-1c: Sterol-regulatory element-binding protein-1c
WAT: white adipose tissue
WT: wild type
