# Supplementary material for: Hepatocyte specific expression of an oncogenic variant of β-catenin results in lethal metabolic dysfunction in mice
Source: Oncotarget. 2018 Jan 30;9(13):11243–57. doi: 10.18632/oncotarget.24346 (PMC5834276; doi:10.18632/oncotarget.24346)
Supplement: Supplementary file 1 [file oncotarget-09-11243-s001.pdf]

Hepatocyte specific expression of an oncogenic variant of β-catenin results in lethal metabolic dysfunction in mice

SUPPLEMENTARY MATERIALS

Supplementary Table 1: Whole liver proteomics. List of al proteins detected in whole liver proteomics of 3 Ctnnb1CA hep mice and 3 WT littermates. Proteins are sorted according their gene name in alphabetic order. See Supplementary_Table_1

Supplementary Table 2: Significant deregulated proteins of metabolism. List of proteins involved in metabolic pathways and energy homeostasis. Significantly up-regulated proteins are marked in pink, significantly down-regulated in green. Livers of three mice of each genotype were analyzed in proteomics. Obtained data were evaluated by *T*-test (difference of mean) and variance analysis within groups. Values of p-value < 0.05; 0.58 log2 fold change in expression were considered significant. See Supplementary_Table_2

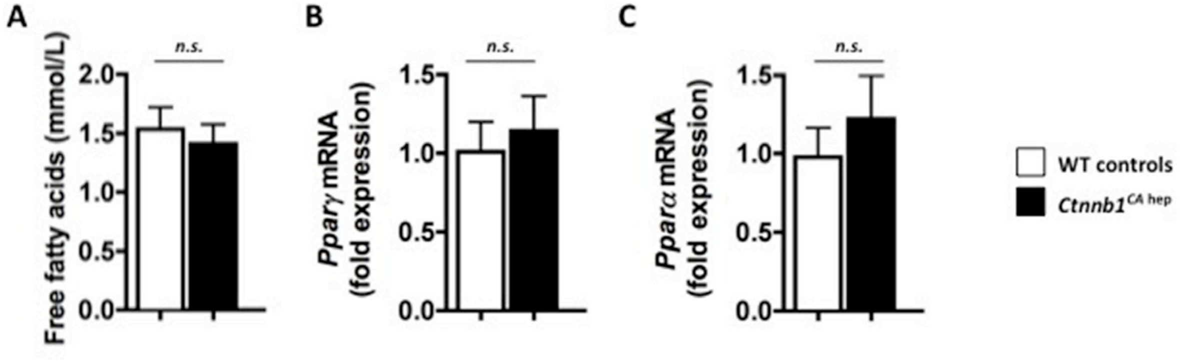

Supplementary Figure 1: mRNA data of key molecules in liver homeostasis, which were not significantly deregulated.
