# Supplementary material for: Hepatocyte specific expression of an oncogenic variant of β-catenin results in lethal metabolic dysfunction in mice
Source: Oncotarget. 2018 Jan 30;9(13):11243–57. doi: 10.18632/oncotarget.24346 (PMC5834276; doi:10.18632/oncotarget.24346)
Supplement: Supplementary file 2 [file oncotarget-09-11243-s002.docx]

| **Gene name** | ***Ctnnb1^CA hep^* vs. WT**  **p-value** | ***Ctnnb1^CA hep^* vs. WT**  **Log 2 Difference** |
| --- | --- | --- |
| A16A1 | 0,640332 | 0,0842743 |
| A16L1 | 0,180133 | 0,832646 |
| A1AT2 | 0,0970651 | -0,360855 |
| A1AT3 | 0,031435 | -0,578688 |
| A1AT4 | 0,00372142 | -0,633307 |
| A1CF | 0,827189 | 0,03088 |
| A2AP | 0,249875 | -0,131613 |
| A2M | 0,0157445 | 0,373359 |
| A7L3B | 0,878777 | -0,0690962 |
| AAAD | 0,77882 | -0,0508296 |
| AAAS | 0,476569 | -1,01111 |
| AACS | 1,75E-06 | 4,28662 |
| AADAT | 0,000440372 | -1,03046 |
| AAKB1 | 0,00178022 | -1,704 |
| AAKG1 | 0,431315 | -0,32292 |
| AAPK1 | 0,00740579 | -0,908506 |
| AAPK2 | 0,00345304 | -0,482321 |
| AASD1 | 0,666649 | 0,102519 |
| AASS | 0,0383201 | -0,312536 |
| AATC | 1,03E-05 | -1,36163 |
| AATM | 0,000214121 | -0,553895 |
| ABC8B | 0,130948 | -0,626469 |
| ABCA1 | 0,444069 | -0,550703 |
| ABCA6 | 0,469999 | -0,101952 |
| ABCB6 | 0,467489 | 0,417193 |
| ABCB7 | 0,719156 | -0,0770871 |
| ABCB8 | 0,359513 | -0,312094 |
| ABCBA | 0,0537522 | -0,254958 |
| ABCBB | 0,0429196 | 0,138163 |
| ABCD1 | 0,00085375 | 1,52221 |
| ABCD2 | 0,608718 | 0,0804564 |
| ABCD3 | 7,28E-06 | 1,06345 |
| ABCE1 | 0,50142 | 0,111659 |
| ABCF1 | 0,0911765 | 0,263669 |
| ABCF2 | 0,134902 | 0,345825 |
| ABCF3 | 0,245084 | 0,58861 |
| ABCG2 | 0,81347 | -0,0479679 |
| ABCG5 | 0,0039658 | -1,76766 |
| ABCG8 | 0,0107927 | -0,747582 |
| ABD12 | 2,28E-05 | 1,74176 |
| ABH15 | 0,118467 | -0,440791 |
| ABHD1 | 0,032519 | 0,824908 |
| ABHD2 | 0,0108027 | 2,39172 |
| ABHD4 | 0,000357654 | 5,44365 |
| ABHD6 | 0,0136751 | 2,45871 |
| ABHDA | 0,439531 | 0,34724 |
| ABHDB | 0,897542 | 0,0634947 |
| ABHEB | 0,0506872 | -0,310045 |
| ABHGA | 0,47305 | 0,320544 |
| ABI1 | 0,123515 | 1,49415 |
| ABLM1 | 0,583458 | 0,391292 |
| ABLM3 | 0,0603473 | 0,824039 |
| ABRAL | 0,694326 | -0,252038 |
| ACACA | 6,92E-05 | 0,600629 |
| ACAD8 | 0,00116506 | -0,586241 |
| ACAD9 | 0,00310471 | -0,483979 |
| ACADL | 0,000161331 | -0,628378 |
| ACADM | 0,0333829 | -0,422879 |
| ACADS | 0,00134434 | -0,642947 |
| ACADV | 6,90E-06 | -0,65356 |
| ACAP2 | 0,965393 | -0,0328356 |
| ACBD5 | 0,00873824 | 0,445181 |
| ACBP | 0,0619429 | 0,74214 |
| ACD10 | 0,348292 | -0,109296 |
| ACD11 | 0,00620493 | 0,45695 |
| ACDSB | 0,000760605 | -0,695174 |
| ACINU | 0,0651872 | -0,325889 |
| ACL6A | 0,446857 | 0,325002 |
| ACLY | 0,0130108 | 0,382836 |
| ACMSD | 0,426258 | 0,655552 |
| ACNT1 | 0,340207 | 0,184306 |
| ACNT2 | 0,624003 | -0,068526 |
| ACO12 | 0,275904 | -0,1465 |
| ACO13 | 0,23138 | 1,27712 |
| ACOC | 0,86977 | 0,0278724 |
| ACON | 0,230828 | -0,127032 |
| ACOT1 | 7,60E-10 | 1,72282 |
| ACOT2 | 0,00348786 | 1,62116 |
| ACOT3 | 1,33E-05 | 2,99023 |
| ACOT3 | 0,000113816 | 2,39407 |
| ACOT4 | 4,90E-07 | 1,56905 |
| ACOT8 | 0,0160819 | 0,837537 |
| ACOT9 | 0,0969403 | 0,698144 |
| ACOX1 | 0,000154736 | 1,15807 |
| ACOX2 | 0,0662156 | 0,487204 |
| ACOX3 | 0,605567 | 0,264274 |
| ACPM | 0,731569 | 0,302739 |
| ACSA | 0,000158752 | 1,81841 |
| ACSF2 | 0,000721242 | 0,377091 |
| ACSF3 | 0,0952944 | -0,641912 |
| ACSL1 | 2,44E-06 | -0,614971 |
| ACSL3 | 0,0253157 | 1,03514 |
| ACSL4 | 0,00135644 | 1,05573 |
| ACSL5 | 0,947854 | -0,00793425 |
| ACSM1 | 0,884101 | 0,0175343 |
| ACSM3 | 0,000202006 | -0,69915 |
| ACSM5 | 0,348912 | -0,0868394 |
| ACSS3 | 0,00322133 | -0,502466 |
| ACTB | 0,706415 | -0,224223 |
| ACTBL | 0,143879 | 0,582032 |
| ACTC | 0,398032 | 0,207205 |
| ACTG | 0,00230167 | 0,395629 |
| ACTN1 | 0,102934 | -0,33084 |
| ACTN4 | 0,152799 | -0,157097 |
| ACTY | 0,567366 | 0,244483 |
| ACTZ | 0,912569 | 0,0263284 |
| ACY1 | 1,72E-05 | -1,10028 |
| ACY2 | 0,356883 | -0,64648 |
| ACY3 | 0,0587739 | -0,32725 |
| ACYP1 | 0,855475 | 0,14807 |
| ADA | 0,484925 | -0,32798 |
| ADA10 | 0,0566825 | -1,27007 |
| ADAS | 2,03E-05 | 1,59006 |
| ADCK1 | 0,18746 | 1,28768 |
| ADCK3 | 6,72E-05 | -0,919466 |
| ADCK4 | 0,421578 | 0,514386 |
| ADCK5 | 0,303942 | 0,566991 |
| ADDA | 0,0777153 | 0,561204 |
| ADDG | 0,917546 | -0,0667486 |
| ADH1 | 0,365131 | 0,195791 |
| ADH4 | 0,440932 | -0,985337 |
| ADH7 | 0,337947 | 0,172791 |
| ADHX | 0,170802 | -0,0868276 |
| ADK | 0,0905523 | -0,26801 |
| ADNP | 0,60979 | 0,372716 |
| ADPGK | 0,36937 | -0,274261 |
| ADRO | 0,00222002 | -0,551917 |
| ADT1 | 0,000457973 | -0,73088 |
| ADT2 | 0,121002 | -0,280551 |
| ADXL | 0,274221 | -0,52229 |
| AEDO | 0,901898 | -0,0549501 |
| AFAD | 0,024968 | 0,352405 |
| AFAM | 0,010236 | 1,09989 |
| AFG31 | 0,0774119 | -0,333186 |
| AFG32 | 0,0724369 | -0,507332 |
| AGFG1 | 0,480876 | -0,175292 |
| AGFG2 | 0,419977 | 0,765227 |
| AGK | 0,00320018 | -0,46059 |
| AGM1 | 0,694345 | -0,063454 |
| AGO1 | 0,955961 | 0,0395508 |
| AGO2 | 0,0106829 | -0,546136 |
| AGT2 | 0,887907 | 0,0144323 |
| AHR | 0,200257 | 0,429996 |
| AHSA1 | 0,615863 | 0,0663268 |
| AHSA2 | 0,866028 | 0,0899162 |
| AIF1 | 0,420107 | -0,649672 |
| AIFM1 | 0,101352 | 0,22735 |
| AIFM2 | 0,261525 | -0,573217 |
| AIMP1 | 0,245654 | -0,154135 |
| AIMP2 | 0,165801 | 0,548073 |
| AIP | 0,51429 | 0,143152 |
| AK1A1 | 2,47E-05 | 0,846096 |
| AK1CD | 0,0198194 | 0,476638 |
| AK1D1 | 0,026654 | -0,690897 |
| AKAP1 | 0,269574 | 0,337439 |
| AKAP2 | 0,0136241 | -0,961987 |
| AKC1H | 0,845822 | 0,0586519 |
| AKCL2 | 0,109532 | -0,199509 |
| AKNA | 0,827666 | -0,0533864 |
| AKT1 | 0,3842 | -0,34453 |
| AKT2 | 0,440983 | 0,30818 |
| AL1A1 | 1,13E-06 | 3,38673 |
| AL1A7 | 2,95E-06 | 2,43664 |
| AL1B1 | 1,19E-08 | -2,61595 |
| AL1L1 | 0,194236 | -0,0862617 |
| AL3A2 | 5,93E-09 | 1,26802 |
| AL4A1 | 0,40729 | 0,134037 |
| AL7A1 | 0,00207587 | -0,613445 |
| AL8A1 | 3,22E-07 | -0,666031 |
| AL9A1 | 0,314531 | -0,144856 |
| ALAT1 | 0,00241695 | -0,423099 |
| ALAT2 | 6,83E-05 | 0,605698 |
| ALBU | 0,217587 | -0,149652 |
| ALD1 | 6,39E-05 | 1,2824 |
| ALD2 | 0,499269 | -0,982047 |
| ALDH2 | 0,00512242 | 0,390258 |
| ALDOA | 0,57145 | -0,0915712 |
| ALDOB | 3,05E-10 | -1,15212 |
| ALDOC | 0,357543 | 0,609422 |
| ALDR | 0,340789 | -0,259753 |
| ALG2 | 0,645862 | -0,1518 |
| ALG5 | 0,0237165 | 0,996325 |
| ALG8 | 0,330722 | -0,580105 |
| ALKMO | 0,0100798 | 0,827787 |
| ALR | 0,0109628 | -1,2161 |
| ALS2 | 0,73678 | -0,173382 |
| AMACR | 2,48E-08 | 1,04487 |
| AMBP | 0,559175 | -0,128778 |
| AMFR | 0,206412 | 0,930979 |
| AMPB | 0,00602432 | 0,422283 |
| AMPD2 | 0,527714 | -0,171152 |
| AMPE | 0,000192727 | 0,612718 |
| AMPL | 0,403013 | 0,0641737 |
| AMPN | 0,761696 | 0,0372798 |
| AMRP | 0,517254 | -0,191052 |
| AMY1 | 0,981708 | 0,00769075 |
| AMYP | 0,784049 | 0,136491 |
| AN32A | 0,129829 | -0,293075 |
| AN32B | 0,378071 | -0,14792 |
| AN32E | 0,0458984 | 0,668067 |
| ANFY1 | 0,581855 | 0,0586888 |
| ANGL3 | 0,000743856 | -1,04995 |
| ANGT | 0,615921 | -0,0945024 |
| ANK1 | 0,277024 | -0,305069 |
| ANK3 | 0,0584711 | -0,396545 |
| ANM1 | 0,0132241 | 0,343355 |
| ANM5 | 0,0925426 | 0,502067 |
| ANR17 | 0,453569 | 0,340952 |
| ANR28 | 0,217285 | -0,813789 |
| ANT3 | 0,1114 | 0,257181 |
| ANX11 | 0,0564129 | -0,200545 |
| ANXA1 | 0,662426 | 0,0888287 |
| ANXA2 | 0,000339704 | 0,754687 |
| ANXA3 | 0,0495435 | -0,401365 |
| ANXA4 | 0,136201 | -0,173031 |
| ANXA5 | 0,00473707 | 0,380324 |
| ANXA6 | 0,00546167 | -0,25202 |
| ANXA7 | 6,39E-06 | 1,12877 |
| AOFA | 1,76E-08 | 2,38904 |
| AOFB | 1,00E-06 | -0,714646 |
| AOXA | 8,45E-06 | 3,9641 |
| AOXC | 0,000129266 | 1,03742 |
| AP1B1 | 0,0892163 | -0,209676 |
| AP1G1 | 0,448556 | -0,142037 |
| AP1M1 | 0,0585649 | -0,310048 |
| AP1S1 | 0,451485 | 0,605557 |
| AP2A1 | 0,994543 | -0,000875473 |
| AP2A2 | 0,016097 | -0,203422 |
| AP2B1 | 0,440661 | 0,134873 |
| AP2M1 | 0,0935176 | -0,215614 |
| AP2S1 | 0,0248331 | -0,519234 |
| AP3B1 | 0,103924 | 0,329592 |
| AP3D1 | 0,130254 | 0,335686 |
| AP3M1 | 0,257713 | 0,234596 |
| AP3S1 | 0,131263 | 0,793154 |
| AP4A | 0,00129298 | 0,70735 |
| APEH | 0,00264281 | -0,431355 |
| APEX1 | 0,203179 | 0,130865 |
| API5 | 0,151576 | -0,294918 |
| APMAP | 0,293006 | -0,189432 |
| APOA1 | 0,307713 | -0,131852 |
| APOA4 | 0,248561 | -0,245269 |
| APOA5 | 0,653603 | 0,238967 |
| APOB | 0,0143833 | 0,267202 |
| APOC1 | 0,0963483 | 1,61174 |
| APOC3 | 0,692445 | -0,125388 |
| APOD | 0,0385342 | 0,507086 |
| APOE | 0,206158 | 0,166897 |
| APOH | 0,189094 | -0,239182 |
| APOM | 0,131225 | 0,443273 |
| APOO | 0,158573 | -0,321817 |
| APOOL | 0,125039 | -0,350232 |
| APT | 5,17E-06 | 1,6271 |
| AQP1 | 0,190401 | -1,02686 |
| ARAF | 0,351697 | -0,156032 |
| ARAP1 | 0,131935 | 0,841758 |
| ARBK1 | 0,0165298 | -1,65873 |
| ARC1A | 0,118813 | -0,405515 |
| ARC1B | 0,481402 | -0,07689 |
| ARCH | 0,000252899 | 0,982021 |
| ARF1 | 0,966869 | 0,00709375 |
| ARF2 | 0,0239867 | -0,575381 |
| ARF4 | 0,0092698 | 0,4726 |
| ARF5 | 0,0735186 | -0,846136 |
| ARF6 | 0,738156 | -0,16645 |
| ARFG1 | 0,279945 | 0,186148 |
| ARFG2 | 0,638782 | -0,0585636 |
| ARGAL | 0,429313 | 0,351242 |
| ARGI1 | 5,65E-13 | -3,90511 |
| ARHG1 | 0,587266 | -0,192001 |
| ARHG2 | 0,215772 | -0,923742 |
| ARHG7 | 0,323592 | -0,823704 |
| ARHGC | 0,456073 | -0,172266 |
| ARHL2 | 0,036305 | -0,380295 |
| ARI1 | 0,599161 | -0,362763 |
| ARI2 | 0,222057 | 0,194547 |
| ARK72 | 0,013827 | 0,322231 |
| ARL1 | 0,715223 | 0,118533 |
| ARL3 | 0,112049 | -0,723083 |
| ARL8B | 0,334618 | -0,134696 |
| ARLY | 2,79E-05 | -1,16169 |
| ARM10 | 0,311739 | -0,769954 |
| ARMC1 | 0,0627871 | -0,660665 |
| ARMC4 | 0,540442 | -0,607669 |
| ARMC8 | 0,0189172 | 1,33945 |
| ARP19 | 0,0840403 | 1,20812 |
| ARP2 | 0,586434 | -0,0715284 |
| ARP3 | 0,939122 | -0,0110779 |
| ARP5L | 0,268697 | -0,363987 |
| ARPC2 | 0,0345225 | -0,247814 |
| ARPC3 | 0,196686 | -0,539451 |
| ARPC4 | 0,114057 | -0,325638 |
| ARPC5 | 0,35264 | -0,199066 |
| ARRB1 | 0,00842653 | -0,59635 |
| ARRB2 | 0,175494 | -0,629171 |
| ARSB | 0,00653914 | -0,751915 |
| ARVC | 0,839411 | 0,113593 |
| ARY2 | 0,176576 | -0,400194 |
| AS3MT | 0,0697175 | 0,51484 |
| ASAH1 | 0,817046 | -0,26482 |
| ASAP1 | 0,248891 | 0,373602 |
| ASCC3 | 0,0152161 | 1,11474 |
| ASGL1 | 0,000474833 | -2,04647 |
| ASGR1 | 0,946134 | -0,0155506 |
| ASGR2 | 0,665837 | 0,228252 |
| ASNA | 0,38208 | 0,178109 |
| ASNS | 0,0176896 | 1,57677 |
| ASPC1 | 0,0120003 | 0,74569 |
| ASPD | 0,000197799 | -0,820734 |
| ASPG | 0,40823 | -0,429372 |
| ASPH | 0,107842 | -0,302416 |
| ASSY | 4,69E-07 | -0,783678 |
| AT11C | 0,000617991 | 0,5435 |
| AT131 | 0,104677 | -0,366517 |
| AT1A1 | 0,0205892 | -0,304555 |
| AT1A4 | 0,067094 | 1,02477 |
| AT1B1 | 0,21254 | -0,273962 |
| AT1B3 | 0,984126 | -0,00984542 |
| AT2A2 | 0,129663 | -0,153446 |
| AT2B2 | 0,0871673 | -1,50515 |
| AT2L1 | 4,70E-07 | -5,55896 |
| AT5F1 | 0,00404069 | -0,589477 |
| ATAD1 | 0,195786 | 0,353746 |
| ATAD3 | 0,936159 | -0,0114104 |
| ATG2A | 0,288889 | -0,724007 |
| ATG2B | 0,873313 | 0,139774 |
| ATG3 | 0,472435 | -0,232465 |
| ATG4B | 0,0993713 | 0,367744 |
| ATG5 | 0,0420359 | 0,502472 |
| ATG7 | 0,0129018 | 0,294554 |
| ATHL1 | 0,246008 | -0,524987 |
| ATLA2 | 0,00483973 | 0,356632 |
| ATLA3 | 8,62E-05 | 1,11728 |
| ATOX1 | 0,135779 | 0,578648 |
| ATP5H | 0,000438439 | -0,723829 |
| ATP5I | 0,00972835 | -0,517178 |
| ATP5J | 0,0638285 | -0,476236 |
| ATP5L | 0,000266979 | -0,674317 |
| ATP5S | 0,396184 | 0,203297 |
| ATP8 | 0,712216 | 0,6653 |
| ATPA | 0,00048616 | -0,564377 |
| ATPB | 1,08E-05 | -0,535054 |
| ATPD | 0,197838 | -0,594922 |
| ATPF1 | 0,564369 | 0,459518 |
| ATPF2 | 0,00829671 | -0,53605 |
| ATPG | 0,00189734 | -0,786052 |
| ATPK | 0,00760412 | -0,835874 |
| ATPO | 0,0041839 | -0,58345 |
| ATRX | 0,203604 | -0,386213 |
| ATTY | 0,499109 | 0,242673 |
| ATX10 | 0,0308421 | 0,557536 |
| ATX2 | 0,0964711 | 0,457035 |
| ATX2L | 0,212044 | 0,424395 |
| AUHM | 0,00931871 | -0,516178 |
| AUP1 | 0,150824 | 0,998864 |
| B2CL1 | 0,50447 | -0,319151 |
| B2L13 | 0,00359384 | -0,453366 |
| B2MG | 0,962384 | 0,0128822 |
| B3AT | 0,395671 | -0,440346 |
| B4GN1 | 0,0493439 | -1,12101 |
| B4GT1 | 0,413125 | 0,516806 |
| BAAT | 0,0376766 | -0,68126 |
| BABA1 | 0,417313 | 0,474709 |
| BACH | 0,627492 | 0,103421 |
| BAF | 0,578981 | 0,557902 |
| BAG3 | 0,0305697 | 0,553921 |
| BAG5 | 0,185293 | -0,805038 |
| BAG6 | 0,0665628 | 0,362004 |
| BAIP2 | 0,998119 | 0,000252088 |
| BAK | 0,352468 | 0,596565 |
| BAP31 | 0,71667 | 0,0950104 |
| BASI | 0,357004 | -0,195608 |
| BAX | 0,190291 | 0,494482 |
| BAZ1B | 0,924516 | -0,0325324 |
| BCAP | 0,000276632 | 1,46611 |
| BCAS3 | 0,163351 | 0,841834 |
| BCCIP | 0,423688 | -0,166707 |
| BCDO1 | 0,869699 | 0,124447 |
| BCDO2 | 0,0980051 | 0,348573 |
| BCLF1 | 0,0466116 | 0,4683 |
| BCS1 | 0,011373 | -0,550278 |
| BDH | 0,0402374 | -0,255536 |
| BECN1 | 0,0380349 | -1,07704 |
| BET1 | 0,625742 | -0,425214 |
| BGAL | 0,00342182 | -2,39473 |
| BGH3 | 0,572138 | 0,180903 |
| BGLR | 0,00528694 | -1,49937 |
| BHMT1 | 0,000277589 | 0,702691 |
| BHMT2 | 0,0251434 | 1,6244 |
| BI1 | 0,952509 | -0,0340033 |
| BI2L1 | 2,39E-06 | 2,00807 |
| BICD2 | 0,00480023 | 3,2928 |
| BIEA | 0,272788 | -0,139921 |
| BIG1 | 0,0487184 | 0,356045 |
| BIG2 | 0,489894 | 0,505098 |
| BIN1 | 0,00336712 | -1,31177 |
| BIN2 | 0,896749 | -0,0622225 |
| BIRC6 | 0,436154 | 0,176504 |
| BLMH | 0,957258 | -0,0110661 |
| BLVRB | 3,82E-08 | 1,29383 |
| BODG | 0,00124663 | -0,547203 |
| BOLA1 | 0,679006 | 0,137944 |
| BOLA2 | 0,126712 | 0,719851 |
| BOLA3 | 0,864504 | -0,125633 |
| BPHL | 0,00577545 | -0,533502 |
| BPNT1 | 0,677009 | -0,0521396 |
| BRAF | 0,22881 | -1,3706 |
| BRAP | 0,00305541 | 1,10874 |
| BRCC3 | 0,956084 | -0,0206003 |
| BRD3 | 0,270325 | -0,344076 |
| BRD4 | 0,70495 | -0,159154 |
| BRE | 0,31594 | -0,441626 |
| BRE1A | 0,467739 | 0,430316 |
| BRI3B | 0,635107 | -0,147012 |
| BRK1 | 0,686025 | -0,128418 |
| BT3L4 | 0,998084 | -0,00180149 |
| BTD | 0,535496 | -0,272958 |
| BTF3 | 0,842331 | -0,0578992 |
| BUB3 | 0,383784 | -0,189885 |
| BUD31 | 0,373144 | 0,651735 |
| BUP1 | 0,0163987 | -0,659033 |
| BYST | 0,0261073 | 0,859802 |
| BZW1 | 0,870242 | -0,0260127 |
| BZW2 | 0,895334 | 0,0714588 |
| C10 | 0,830348 | -0,194585 |
| C19L1 | 0,015219 | -0,777369 |
| C1QA | 0,157676 | -0,80645 |
| C1QB | 0,819661 | -0,156926 |
| C1QBP | 0,594153 | -0,165982 |
| C1QC | 0,33968 | -0,52104 |
| C1RL | 0,0636819 | 2,00559 |
| C1TC | 0,0111328 | -0,31188 |
| C1TM | 0,0823822 | -0,389936 |
| C2C2L | 0,104247 | 1,31049 |
| C43BP | 0,263186 | 0,202574 |
| C4BPA | 0,000517759 | 1,66103 |
| C560 | 0,43092 | -0,298567 |
| CA050 | 0,480177 | 0,593877 |
| CA123 | 0,981683 | 0,0194438 |
| CAB39 | 0,80699 | 0,0578165 |
| CAB45 | 0,834993 | -0,0650657 |
| CACP | 0,505291 | 0,06215 |
| CADH1 | 0,696382 | -0,189252 |
| CADH2 | 0,713879 | 0,0860914 |
| CADH5 | 0,0160269 | -0,563761 |
| CAF17 | 0,673829 | -0,151801 |
| CAH1 | 0,54781 | -0,169333 |
| CAH14 | 0,000595657 | -2,7086 |
| CAH2 | 0,000946178 | 1,26904 |
| CAH3 | 0,00138105 | 1,16941 |
| CAH5A | 0,0132699 | -0,668296 |
| CAH8 | 0,109741 | -0,476982 |
| CALM | 0,112155 | 0,457112 |
| CALR | 0,313324 | -0,190248 |
| CALRL | 0,991653 | 0,00516446 |
| CALU | 0,51544 | -0,210406 |
| CALX | 0,209799 | 0,139357 |
| CAMP3 | 0,450743 | 0,161103 |
| CAN1 | 0,075779 | 0,554848 |
| CAN2 | 0,125779 | -0,308521 |
| CANB1 | 0,606545 | 0,517713 |
| CAND1 | 0,385787 | -0,098272 |
| CAP1 | 0,316287 | 0,108381 |
| CAPG | 0,924788 | 0,0521304 |
| CAPR1 | 0,0263713 | 0,397847 |
| CAPZB | 0,781235 | 0,205593 |
| CAPZB | 0,581774 | -0,0712137 |
| CARF | 0,652412 | -0,136142 |
| CARM1 | 0,729696 | -0,196139 |
| CASP3 | 0,278318 | -0,902512 |
| CASP6 | 0,0020258 | -0,876609 |
| CASP7 | 0,650383 | 0,172625 |
| CASP8 | 0,48059 | 0,166617 |
| CATA | 0,256721 | -0,0987695 |
| CATB | 0,126851 | 0,265595 |
| CATC | 0,112511 | -0,518547 |
| CATD | 0,858949 | 0,033254 |
| CATF | 0,0267884 | -1,37597 |
| CATH | 0,366334 | 0,323253 |
| CATL1 | 0,0018329 | 2,77552 |
| CATS | 0,0899821 | 0,399508 |
| CATZ | 0,953247 | -0,00644938 |
| CAZA1 | 0,491197 | 0,167268 |
| CAZA2 | 0,381137 | -0,114262 |
| CB043 | 0,0706228 | -0,931419 |
| CB047 | 0,322138 | -0,289253 |
| CB072 | 0,61212 | -0,415036 |
| CB072 | 0,205811 | -0,68113 |
| CB39L | 0,98272 | 0,0162652 |
| CBG | 5,25E-05 | 1,59629 |
| CBPD | 0,111853 | -0,640209 |
| CBPQ | 0,128075 | -0,324613 |
| CBR1 | 0,00670706 | -0,403805 |
| CBR4 | 0,00879718 | -0,548102 |
| CBS | 2,53E-05 | -1,35839 |
| CBX1 | 0,63548 | 0,471361 |
| CBX3 | 0,626133 | -0,119202 |
| CBX5 | 0,0758832 | -0,936409 |
| CC124 | 0,28899 | 0,491676 |
| CC50A | 0,0758462 | 2,12281 |
| CC90B | 0,214407 | -0,843142 |
| CCAR1 | 0,5164 | 0,0851676 |
| CCAR2 | 0,113028 | -0,225838 |
| CCD22 | 0,128381 | -0,686896 |
| CCD25 | 0,384914 | 0,542517 |
| CCD47 | 0,160785 | -0,195078 |
| CCD51 | 0,426855 | -0,220851 |
| CCD53 | 0,6688 | 0,230018 |
| CCD58 | 0,22308 | -0,606343 |
| CCD93 | 0,0448588 | -1,99498 |
| CCHL | 0,265514 | 0,232857 |
| CCNT1 | 0,520778 | -0,444852 |
| CCNY | 0,21155 | 0,307919 |
| CCS | 0,535608 | 0,427258 |
| CCZ1 | 0,0514065 | 1,42484 |
| CD11B | 0,0422263 | 0,372136 |
| CD123 | 0,340954 | -0,360709 |
| CD166 | 0,125078 | -0,488817 |
| CD1D1 | 0,713586 | 0,421699 |
| CD2AP | 0,00516205 | 0,91522 |
| CD302 | 0,282983 | -0,300863 |
| CD36 | 0,121973 | -0,368412 |
| CD38 | 0,0497215 | -0,570367 |
| CD47 | 0,0235817 | 0,646402 |
| CD81 | 0,531911 | 0,168586 |
| CD82 | 0,160181 | 0,661271 |
| CDC16 | 0,121169 | 0,712546 |
| CDC23 | 0,288583 | 0,705072 |
| CDC37 | 0,473903 | -0,144331 |
| CDC42 | 0,586624 | 0,782125 |
| CDC42 | 0,797713 | 0,109084 |
| CDC5L | 0,291548 | -0,243997 |
| CDC73 | 0,497455 | 0,680849 |
| CDD | 0,246997 | 0,470069 |
| CDIPT | 0,745609 | -0,0756744 |
| CDK1 | 0,399072 | 0,450589 |
| CDK13 | 0,862977 | -0,0942329 |
| CDK17 | 0,718352 | -0,214165 |
| CDK5 | 0,0616987 | 1,11853 |
| CDK6 | 0,0133838 | 0,517985 |
| CDK7 | 0,274939 | 1,03504 |
| CDN2C | 0,0446601 | 0,800308 |
| CDO1 | 5,25E-06 | -4,15988 |
| CDS2 | 0,0854903 | 0,648634 |
| CDV3 | 0,985509 | -0,00664012 |
| CE051 | 0,706637 | 0,273935 |
| CEAM1 | 4,30E-05 | -1,60461 |
| CECR5 | 0,0239174 | -0,795647 |
| CELF1 | 0,907116 | -0,0186179 |
| CELF2 | 0,00588462 | 1,26017 |
| CENPV | 0,620617 | 0,0750176 |
| CEPT1 | 0,127942 | -0,934478 |
| CERS2 | 0,105699 | 0,782216 |
| CERU | 0,506719 | 0,105471 |
| CES1D | 0,95081 | 0,0109994 |
| CF211 | 0,24461 | 0,643542 |
| CF226 | 0,194482 | 1,07589 |
| CFAB | 0,504804 | 0,0697559 |
| CFAH | 0,00175847 | -0,692353 |
| CFAI | 0,561498 | -0,144402 |
| CFDP1 | 0,715733 | 0,177658 |
| CGL | 0,144934 | -0,572063 |
| CGNL1 | 0,520274 | 0,346641 |
| CH082 | 0,00439928 | -0,873767 |
| CH10 | 0,272346 | -0,391268 |
| CH60 | 0,000530542 | -0,458851 |
| CHAC2 | 0,055587 | -0,76985 |
| CHCH1 | 0,218461 | 1,06494 |
| CHCH2 | 0,000643279 | -0,834656 |
| CHCH3 | 0,0164855 | -0,298809 |
| CHCH5 | 0,027241 | 1,50123 |
| CHD4 | 0,526515 | 0,135125 |
| CHDH | 0,0256457 | -0,44095 |
| CHERP | 0,81092 | -0,0846554 |
| CHIP | 0,293246 | 0,548593 |
| CHKB | 0,875279 | 0,0452439 |
| CHLE | 0,00921513 | -0,597824 |
| CHM1A | 0,529256 | 0,752803 |
| CHM2A | 0,172832 | -0,49932 |
| CHM4B | 0,492653 | 0,303316 |
| CHMP6 | 0,244238 | -0,561968 |
| CHP1 | 0,186247 | 0,215285 |
| CHRD1 | 0,975725 | -0,00485961 |
| CHSP1 | 0,145623 | -0,555692 |
| CIA30 | 0,297101 | -0,189943 |
| CIAO1 | 0,659227 | -0,284012 |
| CIDEB | 0,196351 | 0,723609 |
| CING | 0,167045 | -0,325669 |
| CISD1 | 0,00445256 | -0,634412 |
| CISD2 | 0,328285 | -0,228338 |
| CISY | 0,000443084 | 0,935628 |
| CK054 | 0,00385414 | 0,523 |
| CK068 | 0,154133 | -0,608156 |
| CK083 | 0,000357343 | -2,33115 |
| CK5P3 | 0,314889 | -0,167809 |
| CKAP4 | 0,00748996 | -0,470184 |
| CKAP5 | 0,677849 | -0,177087 |
| CLAP1 | 0,0633071 | 1,27629 |
| CLC2D | 0,151427 | -0,491761 |
| CLC4F | 0,0494368 | -0,377899 |
| CLC4G | 0,276259 | 0,845141 |
| CLCA | 0,314732 | -1,13856 |
| CLCB | 0,109605 | 0,218932 |
| CLCC1 | 0,655323 | -0,130261 |
| CLD3 | 0,0393936 | 0,482853 |
| CLH1 | 0,610893 | -0,0498556 |
| CLIC1 | 0,923992 | 0,0291004 |
| CLIC4 | 0,926101 | 0,017526 |
| CLIP1 | 0,283071 | 0,339605 |
| CLP1L | 0,494576 | 0,365712 |
| CLPB | 0,010299 | 0,597692 |
| CLPP | 0,615867 | 0,0708281 |
| CLPT1 | 0,0665467 | 1,36812 |
| CLPX | 0,156702 | 0,269727 |
| CLU | 0,556654 | 0,0466919 |
| CLUS | 0,00160359 | 0,817613 |
| CLYBL | 0,00194123 | -0,552108 |
| CMAH | 0,160242 | -0,977992 |
| CMBL | 0,0041014 | 0,950515 |
| CMC1 | 0,000368564 | -0,735101 |
| CMC2 | 0,0821434 | -0,223258 |
| CMLO1 | 0,400629 | 0,191689 |
| CMLO2 | 4,54E-05 | 1,40571 |
| CMPK2 | 0,742072 | 0,125038 |
| CMTR1 | 0,298179 | -0,677224 |
| CN159 | 0,0106917 | -0,637306 |
| CN166 | 0,0208802 | 0,410663 |
| CN37 | 0,00412159 | -1,17233 |
| CNBP | 0,775871 | 0,115772 |
| CNDP2 | 0,125776 | -0,103517 |
| CNN2 | 0,444241 | 0,490386 |
| CNN3 | 0,401346 | 0,202579 |
| CNOT1 | 0,813583 | -0,021671 |
| CNOT2 | 0,607449 | -0,339193 |
| CNOT3 | 0,0998312 | -1,32753 |
| CNPY2 | 0,363415 | 0,182975 |
| CNPY3 | 0,396136 | -0,257125 |
| CNPY4 | 0,686607 | 0,0928485 |
| CO1A1 | 0,00465165 | -0,630212 |
| CO1A2 | 0,221063 | 0,286407 |
| CO2A1 | 0,402325 | -0,230799 |
| CO3 | 0,000210599 | 0,350473 |
| CO3A1 | 0,0769602 | -0,792792 |
| CO4A2 | 0,00288946 | 0,580421 |
| CO4B | 4,04E-06 | -1,04099 |
| CO5 | 0,00651268 | -1,09482 |
| CO6A1 | 0,131795 | -0,215551 |
| CO6A2 | 0,0351497 | -0,313879 |
| CO8A | 0,0102925 | -0,979114 |
| CO8B | 0,000283298 | -1,12826 |
| CO8G | 0,305092 | -0,476114 |
| COA3 | 0,00966618 | -0,568966 |
| COA7 | 0,554477 | 0,142416 |
| COAC | 0,224347 | 0,370962 |
| COASY | 0,887939 | 0,0311492 |
| COBL1 | 0,0153571 | 0,397284 |
| COEA1 | 0,00610347 | -0,269795 |
| COF1 | 0,134588 | -0,198829 |
| COF2 | 0,0404568 | -0,43559 |
| COG1 | 0,0959738 | 0,438739 |
| COG4 | 0,35345 | 0,988728 |
| COG5 | 0,673566 | -0,209196 |
| COG7 | 0,245466 | 1,04874 |
| COIA1 | 0,00378746 | 0,664312 |
| COMD1 | 0,249304 | -0,143684 |
| COMD2 | 0,996894 | 0,00114377 |
| COMD3 | 0,7256 | 0,0843566 |
| COMD6 | 0,944925 | 0,0261771 |
| COMD7 | 0,354533 | -0,550077 |
| COMD9 | 0,0701223 | -0,751563 |
| COMDA | 0,371643 | -0,256 |
| COMT | 0,00119305 | 1,06316 |
| COPA | 0,804109 | 0,0259008 |
| COPB | 0,386425 | -0,0511675 |
| COPB2 | 0,0814904 | -0,148328 |
| COPD | 0,367321 | -0,115658 |
| COPE | 0,483505 | -0,111553 |
| COPG1 | 0,104854 | -0,121112 |
| COPG2 | 0,329465 | 0,130842 |
| COPT1 | 0,575045 | -0,121177 |
| COPZ1 | 0,431316 | 0,162763 |
| COPZ2 | 0,00119622 | -2,16017 |
| COQ3 | 0,512148 | 0,1757 |
| COQ4 | 0,837806 | -0,0724583 |
| COQ5 | 0,0605641 | -0,311746 |
| COQ6 | 0,0878754 | 0,215102 |
| COQ7 | 0,564837 | 0,125067 |
| COQ9 | 0,584829 | 0,2286 |
| COR1A | 0,00234162 | 0,534858 |
| COR1B | 0,608873 | -0,0918261 |
| COR1C | 0,00639315 | 1,33878 |
| CORO7 | 0,179817 | -0,241701 |
| COTL1 | 0,932361 | -0,0305026 |
| COX15 | 0,0344942 | -1,27599 |
| COX17 | 0,560094 | 0,436215 |
| COX2 | 0,430737 | -0,524339 |
| COX20 | 0,450896 | 0,77825 |
| COX41 | 0,00134809 | -0,449376 |
| COX5A | 0,00682506 | -0,946393 |
| COX5B | 0,0222848 | -0,47424 |
| COX6C | 0,303523 | -0,435566 |
| COX7C | 0,123958 | -1,37748 |
| COX7R | 0,0274408 | -0,840778 |
| COXM1 | 0,208608 | 1,10636 |
| CP013 | 0,00816872 | -0,648854 |
| CP058 | 0,454978 | -0,253543 |
| CP062 | 0,67722 | 0,416447 |
| CP17A | 0,20025 | 1,05293 |
| CP1A2 | 0,000264638 | 1,35766 |
| CP20A | 0,446845 | 0,161144 |
| CP237 | 0,0419405 | -0,123029 |
| CP240 | 0,21491 | -0,202953 |
| CP254 | 0,036054 | 1,16585 |
| CP255 | 2,12E-06 | 4,07511 |
| CP270 | 4,92E-06 | -1,39576 |
| CP27A | 0,555311 | 0,116006 |
| CP2A4 | 0,249374 | 0,473524 |
| CP2A5 | 7,18E-07 | 3,04355 |
| CP2AC | 3,63E-10 | -1,91403 |
| CP2B9 | 8,86E-07 | -1,63216 |
| CP2BA | 0,263446 | 0,96386 |
| CP2BJ | 0,215952 | 1,11989 |
| CP2CT | 0,00836785 | 0,731645 |
| CP2D9 | 0,137166 | 0,331102 |
| CP2DA | 0,00384129 | 0,455626 |
| CP2DB | 0,583375 | 0,125299 |
| CP2DQ | 0,396851 | 0,196926 |
| CP2E1 | 0,000130632 | 0,659149 |
| CP2F2 | 1,37E-07 | -5,92563 |
| CP2J5 | 0,00196377 | -1,64981 |
| CP39A | 0,139978 | -1,22306 |
| CP3AB | 0,0242173 | 0,58231 |
| CP3AD | 2,43E-07 | -1,60926 |
| CP3AG | 0,00531758 | -1,697 |
| CP3AP | 0,0179649 | -0,616538 |
| CP4AA | 1,87E-06 | 3,66663 |
| CP4AE | 9,62E-06 | 1,16783 |
| CP4F3 | 0,677464 | -0,271658 |
| CP4FE | 0,000781471 | 1,02424 |
| CP4V2 | 1,11E-06 | 1,44053 |
| CP51A | 4,10E-07 | 7,69862 |
| CP7A1 | 1,45E-07 | 3,54931 |
| CP8B1 | 0,000642818 | -2,44144 |
| CPN2 | 0,837296 | -0,110762 |
| CPNE1 | 0,881617 | -0,0262057 |
| CPNE3 | 0,00207329 | -0,556487 |
| CPNS1 | 0,998795 | 0,000220299 |
| CPPED | 0,202915 | -0,620163 |
| CPSF1 | 0,982133 | -0,0107155 |
| CPSF2 | 0,492405 | 0,42368 |
| CPSF3 | 0,133041 | 1,901 |
| CPSF5 | 0,0642246 | -0,358617 |
| CPSF6 | 0,515346 | -0,137507 |
| CPSF7 | 0,0106922 | -0,547254 |
| CPSM | 9,63E-11 | -2,47337 |
| CPT1A | 0,406785 | 0,115796 |
| CPT2 | 0,0185938 | -0,301777 |
| CR1L | 0,0407563 | 0,404988 |
| CREG1 | 3,40E-05 | 1,79725 |
| CREL1 | 0,112866 | -1,36612 |
| CREL2 | 0,230254 | -0,310262 |
| CRIP2 | 0,0158664 | 0,471684 |
| CRK | 0,918397 | 0,0201921 |
| CRKL | 0,762664 | 0,0724211 |
| CRLS1 | 0,0287464 | 0,913824 |
| CRYL1 | 0,00413548 | -1,35571 |
| CRYM | 0,0748731 | -1,80282 |
| CS010 | 0,588971 | -0,610881 |
| CS012 | 0,542317 | 0,477925 |
| CS043 | 0,881656 | -0,0335344 |
| CS060 | 0,0380923 | 1,18025 |
| CS1A | 0,0552162 | -1,03853 |
| CSAD | 0,696728 | -0,0834894 |
| CSDE1 | 0,988955 | 0,00181961 |
| CSK | 0,648579 | 0,0794694 |
| CSK21 | 0,00502809 | 0,585418 |
| CSK22 | 0,703092 | -0,0730432 |
| CSK2B | 0,906343 | 0,134254 |
| CSKP | 0,726224 | 0,190875 |
| CSN1 | 0,215255 | 0,243545 |
| CSN2 | 0,222875 | -0,205862 |
| CSN3 | 0,767261 | 0,0488838 |
| CSN4 | 0,801269 | -0,0334949 |
| CSN5 | 0,234251 | 0,266865 |
| CSN6 | 0,617588 | -0,081653 |
| CSN7A | 0,768856 | 0,0476844 |
| CSN8 | 0,167851 | 0,866259 |
| CSRP1 | 0,0479317 | 0,219027 |
| CSRP2 | 0,00511969 | 2,14609 |
| CSTF1 | 0,136999 | -0,428722 |
| CSTF2 | 0,713755 | 0,0887953 |
| CSTF3 | 0,49258 | -0,107134 |
| CTBL1 | 0,0124205 | -0,741809 |
| CTBP1 | 0,353366 | -0,190947 |
| CTBP2 | 0,163316 | 1,11106 |
| CTDS1 | 0,275931 | 0,524203 |
| CTGE5 | 0,0111019 | 0,437071 |
| CTIF | 0,251036 | -0,524465 |
| CTNA1 | 0,199212 | 0,130031 |
| CTNA2 | 0,507403 | -0,704709 |
| CTNB1 | 0,104657 | 0,179572 |
| CTND1 | 0,00354158 | 0,35331 |
| CTR2 | 0,000434898 | 2,42197 |
| CTU2 | 0,719079 | 0,185373 |
| CUL1 | 0,914802 | -0,022893 |
| CUL2 | 0,581928 | -0,0801614 |
| CUL3 | 0,478597 | 0,178631 |
| CUL4A | 0,00545758 | -0,467504 |
| CUL4B | 0,446293 | 0,279554 |
| CUL5 | 0,965054 | -0,0118262 |
| CUTA | 0,90556 | 0,140476 |
| CUTC | 0,0120857 | -0,593263 |
| CX6A1 | 0,813717 | 0,179576 |
| CX6B1 | 0,0963647 | -0,733412 |
| CX7A2 | 0,119021 | -0,423947 |
| CXAR | 0,00267079 | 0,699753 |
| CXB1 | 0,248077 | -0,317852 |
| CY1 | 0,000592962 | -0,518476 |
| CY250 | 0,0375043 | 0,628824 |
| CYB5 | 0,524958 | 0,300634 |
| CYB5B | 0,0462504 | 0,597033 |
| CYBP | 0,4221 | 0,108335 |
| CYC | 0,111251 | -0,741224 |
| CYFP1 | 0,726191 | -0,0674928 |
| CYTB | 0,117592 | 0,310464 |
| D2HDH | 0,261904 | -0,482141 |
| D39U1 | 0,574324 | 0,350816 |
| DAAM2 | 0,117142 | 0,907274 |
| DAB2 | 0,0968922 | -0,3689 |
| DAD1 | 0,283694 | -0,261444 |
| DAG1 | 0,0190664 | 0,788603 |
| DAP1 | 0,00623814 | 0,912306 |
| DAPK1 | 0,0294623 | -2,42447 |
| DAZP1 | 0,228353 | -0,447761 |
| DBNL | 0,136104 | 0,27615 |
| DC1I2 | 0,755992 | -0,138667 |
| DC1L1 | 0,0836308 | 0,359497 |
| DC1L2 | 0,0764647 | 0,544774 |
| DCA11 | 0,203092 | -0,299071 |
| DCAF8 | 0,235953 | 0,238353 |
| DCAKD | 0,54607 | 0,170447 |
| DCMC | 0,0769111 | -0,274941 |
| DCNL1 | 0,911639 | -0,0280062 |
| DCPS | 0,658669 | -0,135467 |
| DCTN1 | 0,0667652 | 0,190739 |
| DCTN2 | 0,257475 | 0,285658 |
| DCTN3 | 0,285164 | -0,71378 |
| DCTN4 | 0,598814 | 0,0954186 |
| DCTN5 | 0,755988 | -0,145534 |
| DCUP | 0,129909 | 0,175133 |
| DCXR | 0,00180549 | -0,796811 |
| DD19A | 0,173674 | -0,350844 |
| DDAH1 | 0,953531 | 0,00993888 |
| DDAH2 | 0,38968 | -0,179961 |
| DDB1 | 0,867279 | -0,0164967 |
| DDC | 1,35E-05 | 1,5096 |
| DDI2 | 0,000153315 | -0,721608 |
| DDX1 | 0,126214 | 0,147566 |
| DDX17 | 0,749544 | -0,0524441 |
| DDX21 | 0,191639 | 0,381257 |
| DDX27 | 0,285142 | 0,751773 |
| DDX3X | 0,137419 | -0,0847511 |
| DDX3Y | 0,0813003 | 1,31785 |
| DDX41 | 0,226141 | 0,518012 |
| DDX42 | 0,489744 | 0,247544 |
| DDX46 | 0,842634 | 0,0332692 |
| DDX5 | 0,54805 | -0,0837425 |
| DDX58 | 0,0219722 | 0,390915 |
| DDX6 | 0,494351 | -0,10566 |
| DECR | 0,00442684 | -0,625244 |
| DECR2 | 0,438725 | 0,158893 |
| DEK | 0,453227 | -0,144164 |
| DEN4C | 0,101703 | 0,8483 |
| DEN5B | 0,0826993 | 0,7351 |
| DENR | 0,202334 | 0,518152 |
| DEOC | 0,213973 | -0,234102 |
| DEPD7 | 0,00740932 | -0,839795 |
| DERL1 | 0,0844089 | 0,486122 |
| DERL2 | 0,208058 | -0,585652 |
| DESM | 0,0722399 | -0,745216 |
| DESP | 0,020717 | -0,522685 |
| DEST | 0,0401279 | 0,477988 |
| DGAT1 | 0,503546 | -0,572334 |
| DGUOK | 0,462343 | -0,346264 |
| DHAK | 1,95E-05 | -0,790414 |
| DHB11 | 0,0374528 | 0,481197 |
| DHB12 | 0,000729123 | 0,801427 |
| DHB13 | 0,00109506 | -1,3809 |
| DHB2 | 0,00126619 | -0,965379 |
| DHB4 | 1,12E-05 | 1,11044 |
| DHB5 | 1,92E-05 | 1,11436 |
| DHB7 | 8,78E-05 | 3,3553 |
| DHB8 | 0,0103959 | -0,508866 |
| DHC24 | 6,94E-06 | 1,9133 |
| DHCR7 | 0,0315792 | 1,20028 |
| DHDH | 0,0878996 | -0,297502 |
| DHE3 | 0,000911017 | -0,755435 |
| DHI1 | 3,18E-06 | -2,14815 |
| DHPR | 0,838637 | -0,0321773 |
| DHR11 | 0,595009 | -0,16299 |
| DHR13 | 0,180344 | 0,351771 |
| DHRS1 | 0,00995802 | 0,453105 |
| DHRS3 | 0,618561 | 0,308566 |
| DHRS4 | 0,505008 | -0,225691 |
| DHRS7 | 0,492568 | 0,818762 |
| DHRSX | 0,492097 | -0,356961 |
| DHSD | 0,266998 | -1,00215 |
| DHSO | 2,31E-05 | 0,69259 |
| DHTK1 | 0,323525 | 0,349892 |
| DHX15 | 0,856474 | 0,0274824 |
| DHX29 | 0,514905 | 0,304211 |
| DHX30 | 0,7463 | 0,0785074 |
| DHX36 | 0,12572 | 1,17346 |
| DHX58 | 8,92E-06 | 1,99562 |
| DHX9 | 0,931905 | 0,012743 |
| DI3L2 | 0,491471 | 0,187115 |
| DIA1 | 6,97E-05 | -1,77588 |
| DIAC | 0,971656 | -0,0149816 |
| DIAP1 | 0,761942 | -0,0511227 |
| DIAP2 | 0,0385419 | 2,05405 |
| DIC | 0,0903609 | -0,35245 |
| DIDO1 | 0,241907 | -0,642461 |
| DIP2B | 0,222583 | 0,514974 |
| DJB11 | 0,643782 | -0,0872189 |
| DJB12 | 0,0267965 | 0,603366 |
| DJC10 | 0,124634 | -0,39856 |
| DJC11 | 0,254113 | -0,160918 |
| DJC22 | 0,14539 | 0,867236 |
| DJC25 | 0,096914 | -1,013 |
| DKC1 | 0,262006 | 0,192192 |
| DLDH | 0,112936 | -0,184819 |
| DLG1 | 0,08384 | -0,226876 |
| DLRB1 | 0,160568 | 0,308742 |
| DMD | 0,057987 | 0,955757 |
| DNJA1 | 0,504804 | -0,0904519 |
| DNJA2 | 0,100904 | -0,437005 |
| DNJA3 | 0,11563 | -0,18706 |
| DNJB1 | 0,841641 | 0,0506897 |
| DNJB4 | 0,00635728 | 0,378103 |
| DNJC1 | 0,401807 | 0,1959 |
| DNJC2 | 0,189231 | -1,08942 |
| DNJC3 | 0,931281 | -0,022198 |
| DNJC5 | 0,260624 | -0,538993 |
| DNJC7 | 0,000663024 | 0,962915 |
| DNLI3 | 0,167408 | -0,734201 |
| DNLZ | 0,0333964 | 1,14345 |
| DNM1L | 0,42349 | -0,144012 |
| DNPEP | 7,41E-05 | 0,45424 |
| DNPH1 | 0,796183 | 0,385776 |
| DNS2A | 0,207501 | 0,712366 |
| DOCK7 | 0,988785 | 0,00861549 |
| DOPD | 0,370502 | 0,175535 |
| DP13A | 0,898638 | 0,0316505 |
| DPEP1 | 0,00187381 | 1,67938 |
| DPM1 | 0,190276 | 0,645239 |
| DPOD1 | 0,195485 | -0,655884 |
| DPP2 | 0,292983 | -0,149475 |
| DPP3 | 0,0317376 | 0,338153 |
| DPP4 | 0,66365 | -0,0373627 |
| DPP9 | 0,120759 | -0,728497 |
| DPTOR | 0,00895645 | -0,549691 |
| DPYD | 7,48E-05 | -0,780238 |
| DPYL2 | 0,0270115 | -0,315343 |
| DPYL3 | 0,58408 | -0,133002 |
| DPYS | 0,0432068 | -0,269032 |
| DRG1 | 0,800363 | 0,0596759 |
| DRG2 | 0,288958 | -0,481054 |
| DRS7B | 0,275134 | 0,23919 |
| DSC2 | 0,000825787 | -0,649498 |
| DSCR3 | 0,365966 | 0,649847 |
| DSG2 | 0,441497 | -0,120299 |
| DSG3 | 0,237109 | -0,916317 |
| DTD2 | 0,621218 | 0,219933 |
| DTX3L | 0,0594676 | 0,396115 |
| DUS23 | 0,95493 | -0,037864 |
| DUS3 | 0,00472118 | -0,550371 |
| DX39A | 0,895403 | 0,113371 |
| DX39B | 0,990242 | 0,000908534 |
| DYH12 | 0,486432 | -0,708309 |
| DYH17 | 0,279823 | 1,68164 |
| DYHC1 | 0,0228361 | 0,177155 |
| DYL1 | 0,451164 | 0,738635 |
| DYL2 | 0,925174 | 0,0299168 |
| DYM | 0,19602 | 0,952592 |
| DYN2 | 0,362719 | -0,102784 |
| DYN3 | 0,570871 | -0,317687 |
| DYR | 0,0400566 | -0,414788 |
| DYSF | 0,0162742 | -1,34379 |
| DYST | 0,801031 | 0,166141 |
| E2AK2 | 0,0343501 | 1,0779 |
| E41L2 | 0,598773 | -0,106307 |
| E41L5 | 0,200095 | 0,391557 |
| EAA2 | 2,10E-10 | 8,19148 |
| EBP | 0,413983 | -0,607469 |
| ECE1 | 0,000366826 | -0,681756 |
| ECH1 | 0,768129 | -0,046353 |
| ECHA | 0,000394455 | -0,48903 |
| ECHB | 0,00864023 | -0,471555 |
| ECHD1 | 0,143771 | 0,344021 |
| ECHD2 | 0,166753 | 0,141099 |
| ECHD2 | 0,62921 | -0,318814 |
| ECHD3 | 0,212657 | -0,325049 |
| ECHM | 0,0130188 | -0,630362 |
| ECHP | 0,000360958 | 0,815145 |
| ECI1 | 0,00190027 | -0,371098 |
| ECI2 | 0,285732 | 0,148295 |
| ECM1 | 0,0062143 | -0,923819 |
| ECM29 | 0,361624 | 0,2453 |
| ECSIT | 0,0841219 | 0,473677 |
| EDC4 | 0,484849 | -0,225532 |
| EDF1 | 0,242098 | -0,72785 |
| EEA1 | 0,281489 | -0,190843 |
| EEPD1 | 0,220748 | -1,13891 |
| EF1A1 | 0,69124 | 0,0842508 |
| EF1A2 | 0,584823 | -0,140196 |
| EF1B | 0,592979 | -0,163622 |
| EF1D | 0,736904 | -0,0830212 |
| EF1D | 0,204252 | -0,138997 |
| EF1G | 0,701576 | 0,0700957 |
| EF2 | 0,548317 | 0,0657244 |
| EFGM | 0,213657 | 0,137671 |
| EFHD2 | 0,000242242 | 0,976391 |
| EFR3A | 0,323679 | -0,471418 |
| EFTS | 0,671357 | -0,116766 |
| EFTU | 0,0012462 | -0,342992 |
| EGFR | 0,156203 | -0,429152 |
| EGLN | 0,0385947 | 0,374091 |
| EHD1 | 0,690561 | 0,0722281 |
| EHD2 | 0,620384 | -0,377569 |
| EHD3 | 1,06E-06 | -0,79092 |
| EHD4 | 0,00241529 | 0,412822 |
| EI2BA | 0,201338 | 0,407985 |
| EI2BB | 0,554691 | 0,181982 |
| EI2BD | 0,251613 | 0,452545 |
| EI2BE | 0,380783 | 0,179859 |
| EI3JA | 0,0520549 | 0,303738 |
| EIF1 | 0,175203 | 0,522122 |
| EIF2A | 0,459594 | 0,0787903 |
| EIF2D | 0,0144709 | 0,734927 |
| EIF3A | 0,60129 | 0,0423317 |
| EIF3B | 0,353831 | 0,146766 |
| EIF3C | 0,982596 | 0,00226752 |
| EIF3D | 0,276938 | 0,19389 |
| EIF3E | 0,684053 | 0,0630922 |
| EIF3F | 0,35854 | 0,105557 |
| EIF3G | 0,260327 | 0,204104 |
| EIF3H | 0,662701 | -0,201054 |
| EIF3I | 0,280021 | 0,199371 |
| EIF3K | 0,42034 | 0,0995607 |
| EIF3L | 0,133379 | 0,181376 |
| EIF3M | 0,749238 | -0,0713256 |
| EKI2 | 0,00469919 | 0,636741 |
| ELAV1 | 0,52463 | 0,102967 |
| ELMO1 | 0,156077 | -0,210384 |
| ELMO3 | 0,670688 | 0,264529 |
| ELN | 0,279883 | -0,664452 |
| ELOB | 0,258718 | 1,02455 |
| ELOC | 0,560169 | -0,111101 |
| ELOV2 | 0,378477 | 0,76176 |
| ELOV5 | 0,0588544 | 1,62112 |
| ELOV7 | 1,95E-06 | 3,61929 |
| ELP1 | 0,30576 | -0,364959 |
| ELP2 | 0,207019 | 0,142211 |
| ELP3 | 0,571942 | 0,517457 |
| ELYS | 0,15764 | 0,504102 |
| EM55 | 0,0051308 | -0,259221 |
| EMAL2 | 0,00512173 | 1,20195 |
| EMAL3 | 0,52614 | -0,214898 |
| EMAL4 | 0,114813 | -0,495925 |
| EMC1 | 0,0726865 | 0,303282 |
| EMC2 | 0,0382676 | 0,343085 |
| EMC3 | 0,648467 | 0,0987597 |
| EMC4 | 0,11784 | 0,345529 |
| EMC7 | 0,944818 | -0,0163447 |
| EMC8 | 0,88735 | -0,0666978 |
| EMD | 0,475373 | 0,793779 |
| EMIL1 | 0,966365 | -0,00878302 |
| ENDOU | 0,665035 | 0,264673 |
| ENOA | 0,522138 | 0,0857903 |
| ENOPH | 0,498641 | 0,152173 |
| ENPL | 0,00936585 | -0,1404 |
| ENPP1 | 0,176156 | -0,480582 |
| ENTP5 | 1,57E-05 | 1,78138 |
| ENY2 | 0,918816 | -0,132025 |
| EP15R | 0,00980984 | -0,535149 |
| EPB41 | 0,7189 | -0,0458953 |
| EPDR1 | 0,000203064 | -2,5152 |
| EPIPL | 7,53E-05 | 0,965231 |
| EPMIP | 0,0175748 | 0,577306 |
| EPN1 | 0,978818 | 0,0104068 |
| EPN4 | 0,659936 | -0,0807521 |
| EPS15 | 0,010786 | -0,59391 |
| EPT1 | 0,0367792 | 0,970013 |
| ERAP1 | 0,762695 | 0,0393744 |
| ERC2 | 0,81234 | -0,131314 |
| ERF1 | 0,0241238 | 0,307923 |
| ERF3A | 0,740963 | -0,0506776 |
| ERG1 | 2,22E-06 | 2,99447 |
| ERG24 | 0,00400512 | 2,44425 |
| ERG28 | 0,864979 | 0,123109 |
| ERG7 | 4,03E-06 | 2,91501 |
| ERGI1 | 0,96839 | -0,0502249 |
| ERGI3 | 0,840078 | 0,0900052 |
| ERH | 0,366394 | -0,203698 |
| ERLEC | 0,515637 | -0,569377 |
| ERLN1 | 0,728877 | 0,0775674 |
| ERLN2 | 0,664974 | -0,0757109 |
| ERMP1 | 0,58534 | -0,114486 |
| ERO1A | 0,641524 | -0,0632995 |
| ERP29 | 0,658397 | -0,11475 |
| ERP44 | 0,0562812 | -0,249795 |
| ES1 | 0,156179 | -0,305693 |
| ES8L2 | 0,0110881 | 0,464291 |
| EST1 | 0,00398729 | 1,10745 |
| EST1C | 3,10E-05 | 0,827634 |
| EST1E | 0,542919 | 0,482378 |
| EST2A | 0,000145915 | -1,04094 |
| EST2C | 0,000275858 | 2,07965 |
| EST2E | 0,0302455 | -0,286273 |
| EST3A | 0,000151117 | 1,70456 |
| ESTD | 0,0495481 | -0,191233 |
| ESYT1 | 0,00360084 | 0,376614 |
| ESYT2 | 0,280286 | 0,425846 |
| ETFA | 0,00178205 | -0,538297 |
| ETFB | 6,19E-05 | -0,665123 |
| ETFD | 0,0599013 | -0,327679 |
| ETHE1 | 5,53E-06 | -1,13022 |
| ETUD1 | 0,163027 | -0,269495 |
| EVI5 | 0,828247 | -0,302505 |
| EWS | 0,677308 | -0,0638927 |
| EXOC1 | 0,092411 | 0,91557 |
| EXOC2 | 0,259657 | 0,786141 |
| EXOC3 | 0,64007 | 0,15535 |
| EXOC4 | 0,693557 | 0,240222 |
| EXOC5 | 0,0396389 | -0,208134 |
| EXOC7 | 0,781064 | 0,115772 |
| EXOC8 | 0,578975 | 0,118757 |
| EXOS2 | 0,866578 | 0,146187 |
| EXOS3 | 0,845723 | 0,140228 |
| EXOS4 | 0,369012 | -0,379238 |
| EXOS5 | 0,0884349 | -0,659706 |
| EXOS7 | 0,649961 | -0,387528 |
| EXOS8 | 0,329648 | -0,489628 |
| EXOS9 | 0,308138 | 0,254808 |
| EXOSX | 0,348265 | 1,17491 |
| EZRI | 0,506745 | -0,396859 |
| F107B | 0,308068 | 0,773774 |
| F10A1 | 0,331766 | 0,170431 |
| F1142 | 0,482253 | 0,472431 |
| F120A | 0,460135 | 0,112349 |
| F134C | 0,186046 | 1,13807 |
| F136A | 0,753261 | 0,0882769 |
| F162A | 0,0636814 | -0,481974 |
| F16B1 | 0,541658 | 0,145303 |
| F16P1 | 0,000851524 | -0,485545 |
| F175B | 0,180179 | 1,31961 |
| F210A | 0,690664 | -0,122912 |
| F213A | 0,000140928 | -0,734546 |
| F261 | 2,36E-10 | 1,78669 |
| F91A1 | 0,976763 | 0,00500139 |
| FA10 | 0,00302631 | -1,08616 |
| FA12 | 0,741756 | 0,261769 |
| FA45A | 0,216223 | 0,959761 |
| FA49B | 0,998237 | -0,00158278 |
| FA5 | 0,279312 | 0,947712 |
| FA63A | 0,393558 | 0,229184 |
| FA73B | 0,221747 | 0,338626 |
| FA83H | 0,451886 | -0,525504 |
| FA96A | 0,112218 | -0,363785 |
| FA98A | 0,806476 | 0,0816396 |
| FA98B | 0,67522 | 0,16378 |
| FAAA | 0,0020348 | -0,600484 |
| FAAH1 | 0,965395 | 0,00787735 |
| FABD | 0,121511 | -0,510798 |
| FABP4 | 0,00282655 | -0,599043 |
| FABP5 | 7,47E-07 | -2,09476 |
| FABP7 | 0,533818 | -0,563792 |
| FABPI | 0,936791 | 0,0445372 |
| FABP1 | 0,0247784 | 0,281487 |
| FACE1 | 0,478495 | -0,116042 |
| FAD1 | 0,537072 | 0,0832456 |
| FADS1 | 0,00955322 | 1,23161 |
| FADS2 | 1,50E-05 | 1,91446 |
| FAF1 | 0,392521 | 0,301327 |
| FAF2 | 0,99363 | 0,00102838 |
| FAHD1 | 5,90E-05 | -1,89998 |
| FAHD2 | 8,44E-05 | -0,725999 |
| FAK1 | 0,835333 | 0,11541 |
| FAKD2 | 0,202231 | -0,880024 |
| FAM21 | 0,0928777 | 0,978396 |
| FARP1 | 0,710592 | 0,0870841 |
| FARP2 | 0,792438 | -0,160743 |
| FAS | 1,99E-08 | 1,96125 |
| FBLN3 | 0,998488 | 0,00095431 |
| FBLN5 | 0,194984 | 0,877719 |
| FBN1 | 0,650059 | -0,179185 |
| FBRL | 0,102732 | -0,30233 |
| FBX22 | 0,167664 | -0,738986 |
| FBX3 | 0,78888 | -0,0706771 |
| FBX6 | 0,410603 | 0,588442 |
| FCGR2 | 0,0244774 | -0,59323 |
| FCGRN | 0,300795 | -0,159349 |
| FCHO2 | 0,742284 | 0,186816 |
| FCL | 0,0599897 | -0,252181 |
| FDFT | 1,20E-09 | 7,17367 |
| FERM2 | 0,427166 | 0,117323 |
| FETA | 9,79E-07 | 5,03888 |
| FETUA | 0,0362283 | 0,325144 |
| FETUB | 0,118618 | 0,256279 |
| FGGY | 0,193056 | -0,144895 |
| FGL1 | 0,706793 | 0,253738 |
| FHIT | 0,00640867 | -1,09068 |
| FHL1 | 0,039002 | -0,772191 |
| FHOD1 | 0,773887 | 0,116529 |
| FIBB | 0,0207688 | -0,356822 |
| FIBG | 0,000209632 | -0,445848 |
| FINC | 0,0125563 | 0,230893 |
| FIP1 | 0,132938 | -0,590071 |
| FIS1 | 0,631557 | 0,668727 |
| FITM2 | 0,00625746 | 1,92581 |
| FKB11 | 0,19661 | -0,255612 |
| FKB15 | 0,809961 | 0,116467 |
| FKB1A | 0,315528 | -0,200094 |
| FKBP2 | 0,869349 | 0,0247965 |
| FKBP3 | 0,0590901 | -0,375412 |
| FKBP4 | 0,723168 | -0,0524956 |
| FKBP5 | 0,250282 | -0,359092 |
| FKBP8 | 0,0911058 | 0,401003 |
| FLII | 0,989118 | -0,0020237 |
| FLNA | 0,371891 | 0,0824858 |
| FLNB | 1,61E-05 | 0,700358 |
| FLOT1 | 0,101053 | 0,375813 |
| FLOT2 | 0,419147 | 0,0713444 |
| FMO1 | 0,00760716 | 0,372282 |
| FMO2 | 0,244877 | 0,43042 |
| FMO3 | 4,01E-07 | -7,34271 |
| FMO4 | 0,0258193 | -0,651587 |
| FMO5 | 2,55E-07 | 1,81143 |
| FMR1 | 0,629417 | 0,28776 |
| FND3A | 0,0825342 | -0,637269 |
| FND3B | 0,141737 | -0,728035 |
| FNTA | 0,333973 | 0,407833 |
| FPPS | 6,02E-08 | 4,54264 |
| FRDA | 0,555128 | -0,509643 |
| FRIH | 0,439595 | -0,850624 |
| FRIL1 | 0,562512 | -0,687653 |
| FRM4A | 0,318133 | -0,445788 |
| FRRS1 | 0,285168 | 0,655115 |
| FSCN1 | 0,0855219 | -0,439533 |
| FTCD | 1,55E-09 | -2,13083 |
| FUBP1 | 0,673266 | -0,0463899 |
| FUBP2 | 0,0437082 | -0,364747 |
| FUCM | 0,513948 | -0,430099 |
| FUMH | 0,0270719 | -0,27585 |
| FUND2 | 0,666079 | -0,283679 |
| FUS | 0,289805 | 0,244797 |
| FXR1 | 0,166844 | -0,26014 |
| FXR2 | 0,75508 | -0,135601 |
| FYCO1 | 0,026604 | 0,632569 |
| G3BP1 | 0,182143 | 0,25833 |
| G3BP2 | 0,019608 | 0,980928 |
| G3P | 0,271818 | -0,331822 |
| G45IP | 0,249528 | -0,744924 |
| G6PD1 | 0,098975 | -0,667605 |
| G6PE | 0,400283 | -0,0919984 |
| G6PI | 0,00133421 | -0,315771 |
| GABPA | 0,173947 | -0,309692 |
| GABT | 0,139036 | 0,174537 |
| GAK | 0,696596 | 0,0948776 |
| GALE | 0,0258791 | 0,437764 |
| GALK1 | 0,000121023 | -0,991771 |
| GALK2 | 0,178448 | -0,242427 |
| GALM | 0,552406 | -0,198794 |
| GALT | 0,00206448 | -1,03631 |
| GALT2 | 0,690436 | 0,0976229 |
| GAMT | 0,0589152 | -0,518919 |
| GANAB | 0,100616 | -0,146039 |
| GAPD1 | 0,252685 | 0,310106 |
| GAR1 | 0,888524 | -0,0575733 |
| GATM | 0,0432377 | -1,51497 |
| GBA2 | 0,026936 | 1,324 |
| GBB1 | 0,0125973 | 0,569186 |
| GBB2 | 0,73686 | 0,0470578 |
| GBG12 | 0,329968 | 0,107484 |
| GBLP | 0,652033 | -0,044356 |
| GBP2 | 0,0152248 | 0,306646 |
| GBP4 | 0,812814 | 0,122176 |
| GBRAP | 0,90861 | 0,128257 |
| GBRL1 | 0,551114 | 0,114909 |
| GCAA | 0,343387 | -0,195052 |
| GCC2 | 0,0709638 | -1,75072 |
| GCDH | 0,00817295 | -0,591647 |
| GCH1 | 0,217504 | 0,455353 |
| GCKR | 0,104502 | -0,240047 |
| GCP2 | 0,0536119 | 0,952816 |
| GCP60 | 0,87908 | -0,0413879 |
| GCR | 0,0717552 | -0,486807 |
| GCSH | 0,0176947 | -0,715918 |
| GCSP | 2,23E-09 | -9,10681 |
| GCST | 0,00193464 | -1,30221 |
| GCYA3 | 0,775577 | 0,222905 |
| GCYB1 | 0,199103 | -0,431943 |
| GDC | 0,300524 | -0,261529 |
| GDE1 | 0,571129 | 0,346526 |
| GDIA | 0,218615 | -0,119423 |
| GDIB | 0,886808 | -0,0122503 |
| GDIR1 | 0,585621 | 0,113312 |
| GDIR2 | 0,485561 | -0,565814 |
| GELS | 0,42346 | -0,136579 |
| GEPH | 0,000981165 | 0,466002 |
| GET4 | 0,210649 | 0,744802 |
| GFPT1 | 0,333436 | 0,193462 |
| GFRP | 8,78E-06 | -1,01294 |
| GGA1 | 0,886109 | 0,0747894 |
| GGACT | 0,865469 | -0,0513932 |
| GGCT | 0,376943 | 0,219882 |
| GGLO | 6,44E-07 | 1,22905 |
| GHC1 | 9,17E-05 | 1,1006 |
| GHDC | 0,444487 | 0,423384 |
| GID8 | 0,259185 | 0,546014 |
| GILT | 0,0591574 | -1,04987 |
| GIMA4 | 0,503364 | -0,132322 |
| GIT1 | 0,0475803 | 0,562877 |
| GIT2 | 0,553866 | 0,445864 |
| GLCM | 0,684738 | -0,140899 |
| GLCNE | 4,48E-05 | 0,794224 |
| GLCTK | 0,0700241 | -0,674946 |
| GLGB | 0,0240409 | -0,326667 |
| GLNA | 1,09E-11 | 3,44241 |
| GLO2 | 0,24962 | -0,27913 |
| GLOD4 | 0,658047 | -0,0683114 |
| GLPK | 0,538377 | -0,0901559 |
| GLRX1 | 0,082696 | 0,564342 |
| GLRX3 | 0,527931 | 0,135117 |
| GLRX5 | 0,25083 | -0,557436 |
| GLSL | 5,70E-09 | -8,59054 |
| GLTD2 | 0,645214 | -0,105825 |
| GLU2B | 0,0497899 | -0,372267 |
| GLYAL | 7,25E-07 | -1,68762 |
| GLYAT | 0,355275 | -0,188569 |
| GLYC | 0,0298988 | -0,355056 |
| GLYG | 0,493179 | 0,41603 |
| GLYR1 | 0,26932 | 0,205733 |
| GMPPA | 0,576532 | -0,227864 |
| GMPPB | 0,666944 | -0,0994015 |
| GMPR1 | 0,655637 | -0,146211 |
| GMPR2 | 0,406828 | -0,436735 |
| GNA1 | 0,0393325 | 0,221202 |
| GNA11 | 0,349809 | 0,19239 |
| GNA13 | 0,765535 | -0,0573966 |
| GNA14 | 0,000396214 | 2,57067 |
| GNAI2 | 0,20487 | 0,191966 |
| GNAI3 | 0,015693 | 0,596191 |
| GNAQ | 0,144698 | 0,425706 |
| GNAS2 | 0,18776 | 0,272432 |
| GNAT3 | 0,974573 | 0,0266231 |
| GNL1 | 0,367704 | 0,783486 |
| GNMT | 0,00557294 | -0,477556 |
| GNPI1 | 0,720054 | 0,0922702 |
| GNS | 0,73402 | 0,0735321 |
| GNTK | 0,578543 | -0,207834 |
| GOGA2 | 0,298132 | 0,0945241 |
| GOGA3 | 0,693433 | -0,100381 |
| GOGA5 | 0,808569 | 0,0603224 |
| GOLI4 | 0,0635957 | -0,406755 |
| GOLP3 | 0,254164 | -0,623527 |
| GOPC | 0,0553644 | -1,04795 |
| GORS1 | 0,535098 | 0,117798 |
| GORS2 | 0,50932 | -0,110133 |
| GOSR2 | 0,25233 | 0,911012 |
| GPAA1 | 0,793247 | 0,169137 |
| GPAT1 | 0,199429 | 0,443336 |
| GPC5C | 0,341622 | 0,609355 |
| GPCP1 | 2,96E-06 | 4,64596 |
| GPD1L | 0,0665783 | -0,313061 |
| GPDA | 0,138089 | 0,154833 |
| GPDM | 0,0382032 | 0,235175 |
| GPI8 | 0,108093 | 0,30109 |
| GPX1 | 0,00143681 | -0,562598 |
| GPX3 | 0,00789428 | -0,733283 |
| GPX41 | 0,174796 | 0,354423 |
| GPX7 | 0,991572 | 0,00744883 |
| GRAP1 | 0,453144 | -0,230718 |
| GRB10 | 0,00147328 | 2,17149 |
| GRB14 | 0,282927 | -0,658264 |
| GRB2 | 0,206324 | 0,480395 |
| GRB7 | 0,929692 | -0,0555658 |
| GRHPR | 1,28E-05 | -0,753416 |
| GRIN3 | 0,0288514 | -1,41696 |
| GRN | 0,109252 | 0,370879 |
| GRP75 | 0,922168 | -0,01102 |
| GRP78 | 0,352348 | -0,0880458 |
| GRPE1 | 0,0552877 | 0,335479 |
| GSDMD | 0,0205377 | -0,921178 |
| GSH0 | 0,0072185 | 0,460917 |
| GSH1 | 0,149734 | -0,15563 |
| GSHB | 0,012967 | 0,357359 |
| GSHR | 0,0147695 | 0,524962 |
| GSK3A | 0,141935 | 0,308697 |
| GSK3B | 0,326117 | 0,930005 |
| GSLG1 | 0,880016 | -0,0268393 |
| GSTA2 | 0,00310728 | 2,48588 |
| GSTA3 | 0,0049374 | 1,02227 |
| GSTA4 | 6,79E-05 | 0,749232 |
| GSTK1 | 0,81342 | 0,054464 |
| GSTM1 | 1,64E-05 | 1,29158 |
| GSTM2 | 1,73E-12 | 4,07349 |
| GSTM4 | 2,67E-12 | 4,97041 |
| GSTM5 | 0,0229841 | -0,223284 |
| GSTM6 | 3,82E-05 | 3,08288 |
| GSTM7 | 0,0267326 | 0,534687 |
| GSTO1 | 0,00102899 | -0,913789 |
| GSTP1 | 0,0107458 | 0,659997 |
| GSTT1 | 0,70951 | 0,0840689 |
| GSTT2 | 1,65E-05 | 1,45667 |
| GT251 | 0,538037 | -0,208349 |
| GTF2I | 0,876723 | 0,0262874 |
| GTPB1 | 0,727866 | -0,138128 |
| GTPC1 | 0,132799 | -0,963295 |
| GTR2 | 0,00685085 | -0,524032 |
| GUAA | 0,0628853 | -0,220593 |
| GUAD | 0,00315166 | 0,732843 |
| GVIN1 | 0,835658 | -0,072176 |
| GYS1 | 0,189638 | -0,863462 |
| GYS2 | 0,0418358 | 0,292142 |
| H11 | 0,412382 | -0,617291 |
| H12 | 0,00415863 | -1,1671 |
| H13 | 0,0636709 | -1,33736 |
| H14 | 2,93E-05 | -1,57412 |
| H15 | 0,00695344 | -0,974939 |
| H17B6 | 3,42E-09 | -5,43621 |
| H1BP3 | 0,342308 | -0,173277 |
| H2A1H | 0,188561 | -0,237818 |
| H2A2C | 0,0908696 | -1,16355 |
| H2AV | 0,423432 | 0,260438 |
| H2AY | 0,134467 | -0,244948 |
| H2B1A | 0,561073 | -0,523607 |
| H2B1P | 0,874617 | -0,0738732 |
| H33 | 0,519957 | 0,251163 |
| H4 | 0,0143365 | -0,748357 |
| HA10 | 0,00104009 | 1,28858 |
| HA11 | 0,00167694 | 0,502077 |
| HA15 | 0,993745 | 0,00425434 |
| HA1B | 0,0160527 | 0,424328 |
| HABP4 | 0,790753 | -0,239166 |
| HACD3 | 0,000920579 | 0,911568 |
| HACL1 | 0,538675 | 0,086628 |
| HAOX1 | 1,18E-05 | -1,17723 |
| HAOX2 | 0,000684322 | -0,722097 |
| HAP28 | 0,165636 | -0,232576 |
| HAT1 | 0,307453 | 0,815735 |
| HAX1 | 0,365798 | -1,05567 |
| HBA | 0,0876804 | -0,753676 |
| HBB1 | 0,00567404 | -0,363216 |
| HBS1L | 0,0922703 | -0,429694 |
| HCD2 | 3,37E-05 | -1,08674 |
| HCDH | 0,001183 | -0,437163 |
| HCFC1 | 0,353414 | -0,22314 |
| HDAC1 | 0,157063 | -0,281014 |
| HDAC2 | 0,347541 | 0,7278 |
| HDAC6 | 0,000316792 | -1,00022 |
| HDGF | 0,434536 | 0,186331 |
| HDGR2 | 0,900803 | -0,0715392 |
| HDHD2 | 0,0288776 | -0,443098 |
| HDHD3 | 0,646481 | 0,0677331 |
| HEAT3 | 0,384079 | 0,41293 |
| HEBP1 | 0,322201 | -0,177766 |
| HECD1 | 0,899896 | -0,0397428 |
| HECD3 | 0,00164056 | -1,73123 |
| HELZ2 | 0,66271 | 0,103084 |
| HEM1 | 0,00599205 | 1,66778 |
| HEM2 | 0,000178039 | 0,674046 |
| HEM3 | 0,0167745 | 0,681327 |
| HEM4 | 0,222765 | 0,806925 |
| HEM6 | 0,0172368 | 0,342131 |
| HEMH | 0,000344338 | 0,512041 |
| HEMO | 0,218698 | 0,0879628 |
| HEP2 | 0,982871 | -0,00544294 |
| HEPS | 0,0621112 | -0,623188 |
| HERC2 | 0,325155 | -0,83667 |
| HERC4 | 0,148876 | -0,27067 |
| HERP1 | 0,924997 | -0,0616055 |
| HEXA | 0,104138 | -1,21573 |
| HEXB | 0,000552217 | -0,819729 |
| HEXI1 | 0,835807 | 0,280386 |
| HGD | 1,74E-05 | 0,970313 |
| HGNAT | 0,622273 | -0,24158 |
| HGS | 0,563454 | 0,0907332 |
| HIBCH | 0,184573 | -0,209197 |
| HINT1 | 0,464803 | 0,260484 |
| HINT2 | 0,776482 | 0,0705016 |
| HINT3 | 0,879742 | -0,0449677 |
| HIP1R | 2,19E-05 | 1,39752 |
| HIUH | 0,387851 | -0,20272 |
| HLTF | 0,511151 | 0,5076 |
| HM13 | 0,721666 | 0,107247 |
| HMCS1 | 8,78E-09 | 3,6474 |
| HMCS2 | 0,00334507 | -0,541077 |
| HMDH | 0,24002 | -0,627161 |
| HMGA1 | 0,278788 | -0,996422 |
| HMGB1 | 0,0381657 | -0,433185 |
| HMGB2 | 0,526174 | 0,213324 |
| HMGB3 | 0,85112 | -0,126315 |
| HMGCL | 0,000336524 | -0,726673 |
| HMGN5 | 0,0810852 | -0,343957 |
| HMOX1 | 0,371727 | -0,299413 |
| HMOX2 | 0,0366421 | -0,324025 |
| HN1L | 0,126293 | 0,312201 |
| HNF4A | 0,752501 | 0,0696195 |
| HNMT | 0,241549 | -0,875748 |
| HNRDL | 0,135761 | 0,690261 |
| HNRH1 | 0,129264 | 0,165285 |
| HNRH2 | 0,0162647 | 0,427323 |
| HNRL1 | 0,560048 | -0,0754871 |
| HNRL2 | 0,635532 | 0,0503229 |
| HNRLL | 0,318514 | -0,136301 |
| HNRPC | 0,878428 | -0,0231212 |
| HNRPD | 0,503559 | -0,193693 |
| HNRPF | 0,00153492 | -0,43422 |
| HNRPK | 0,884666 | 0,0322666 |
| HNRPL | 0,365472 | 0,0816822 |
| HNRPM | 0,246246 | -0,128875 |
| HNRPQ | 0,600649 | -0,0620597 |
| HNRPU | 0,0110506 | -0,238867 |
| HOGA1 | 5,01E-06 | -1,08174 |
| HOOK2 | 0,60952 | -0,216023 |
| HOOK3 | 0,553791 | 0,14912 |
| HOT | 0,00209154 | -0,352217 |
| HP1B3 | 0,517457 | 0,241813 |
| HPBP1 | 0,361375 | -0,405239 |
| HPCL1 | 0,937937 | -0,0260391 |
| HPPD | 0,201476 | 0,137801 |
| HPRT | 0,585462 | 0,0714715 |
| HPT | 0,704386 | 0,0823243 |
| HRG | 0,659244 | -0,128305 |
| HS105 | 0,170683 | 0,211465 |
| HS71A | 0,000540089 | 1,43476 |
| HS74L | 0,293966 | 0,629774 |
| HS90A | 0,0031231 | 0,374798 |
| HS90B | 0,00419019 | 0,382123 |
| HSDL1 | 0,299675 | 0,755762 |
| HSDL2 | 0,157128 | 0,190083 |
| HSP13 | 0,217212 | -0,327212 |
| HSP72 | 0,0972609 | 0,934749 |
| HSP74 | 0,00821189 | 0,268364 |
| HSP7C | 0,10491 | 0,166927 |
| HSP7E | 0,0467687 | 0,913085 |
| HSPB8 | 0,821345 | -0,100577 |
| HTAI2 | 0,0427601 | 0,370159 |
| HTRA2 | 0,00268836 | -0,620038 |
| HUTH | 1,04E-07 | -8,08334 |
| HUTI | 1,29E-07 | -1,89872 |
| HUTU | 4,91E-10 | -2,35809 |
| HUWE1 | 0,417076 | 0,315929 |
| HVM56 | 0,194451 | 0,31512 |
| HXK1 | 0,0215976 | -0,640313 |
| HXK2 | 0,00137136 | 1,19387 |
| HXK3 | 0,720548 | -0,37948 |
| HXK4 | 4,12E-06 | -3,89407 |
| HYEP | 0,000108638 | 1,61783 |
| HYES | 0,00957731 | 0,494626 |
| HYKK | 0,0235649 | -0,424967 |
| HYOU1 | 0,0644443 | 0,190725 |
| HYPK | 0,161389 | -1,03321 |
| I23O2 | 5,55E-05 | -4,3626 |
| I2BP2 | 0,000371572 | -2,49606 |
| I2BPL | 0,634915 | -0,254261 |
| IAH1 | 0,0107758 | -0,683741 |
| IBP2 | 0,0696172 | -1,53854 |
| IBP4 | 0,579583 | 0,439004 |
| IBP7 | 0,544348 | -0,422221 |
| IBTK | 0,976866 | -0,0152051 |
| IC1 | 0,591084 | 0,150799 |
| ICA | 0,626546 | 0,0812209 |
| ICAL | 0,841176 | 0,0888195 |
| ICAM1 | 0,00328899 | 0,520731 |
| ICLN | 0,283055 | 0,742749 |
| ICT1 | 0,197295 | -0,453205 |
| IDE | 0,00555567 | 0,283006 |
| IDH3A | 0,000370917 | 0,721848 |
| IDHC | 0,116699 | 0,153624 |
| IDHG1 | 0,00963413 | 0,70585 |
| IDHP | 0,128325 | 0,170334 |
| IDI1 | 3,82E-07 | 4,87344 |
| IF1A | 0,976708 | 0,00834179 |
| IF1AX | 0,770326 | -0,0842339 |
| IF2A | 0,0229297 | 0,199396 |
| IF2B | 0,919717 | 0,0292231 |
| IF2B3 | 0,61607 | -0,0552451 |
| IF2G | 0,805298 | 0,0459267 |
| IF2H | 0,982606 | -0,00292842 |
| IF2M | 0,22153 | -0,828398 |
| IF2P | 0,876064 | 0,0397364 |
| IF4A1 | 0,274454 | 0,12409 |
| IF4A2 | 0,987753 | 0,00294367 |
| IF4A3 | 0,728306 | -0,0474707 |
| IF4B | 0,909542 | 0,0227753 |
| IF4E | 0,281775 | 0,112474 |
| IF4E2 | 0,0817268 | -1,4461 |
| IF4G1 | 0,279357 | 0,0998427 |
| IF4G2 | 0,165572 | 0,27992 |
| IF4H | 0,926699 | -0,0204792 |
| IF5 | 0,00335073 | -0,700447 |
| IF5A1 | 0,51047 | 0,0910072 |
| IF6 | 0,296959 | 0,329744 |
| IFI4 | 0,0333447 | 1,26001 |
| IFIH1 | 0,00880706 | 1,95175 |
| IFIT1 | 5,01E-05 | 3,91642 |
| IFIT2 | 0,254143 | 0,931978 |
| IFIT3 | 0,195419 | 0,841134 |
| IFM2 | 0,614616 | -0,289272 |
| IFM3 | 0,0109822 | 0,800557 |
| IGBP1 | 0,874206 | -0,0699991 |
| IGF1R | 0,459719 | -0,696177 |
| IGG2B | 0,118691 | -0,324871 |
| IGHG1 | 4,59E-05 | -0,844583 |
| IGHG3 | 0,00197886 | -0,962891 |
| IGHM | 0,811842 | 0,164938 |
| IGKC | 0,0166966 | -0,435709 |
| IIGP1 | 0,146645 | 0,334513 |
| IKKB | 0,177422 | 0,946831 |
| IL16 | 0,786598 | -0,0884256 |
| ILEUA | 0,711959 | 0,186459 |
| ILF2 | 0,705553 | -0,0614729 |
| ILF3 | 0,814671 | -0,054493 |
| ILK | 0,0226645 | 0,268187 |
| ILKAP | 0,0604272 | -1,17929 |
| ILVBL | 0,00766121 | -0,996107 |
| IMA1 | 0,0641423 | 0,446877 |
| IMA3 | 0,133578 | 0,453955 |
| IMA4 | 0,329885 | -0,967359 |
| IMA5 | 0,805675 | 0,0881939 |
| IMA7 | 0,352047 | 0,114857 |
| IMB1 | 0,15412 | 0,155416 |
| IMDH2 | 0,118059 | 0,18397 |
| IMMT | 0,236048 | -0,144913 |
| IMPA1 | 0,933787 | 0,0114791 |
| IMPA3 | 0,656431 | -0,35143 |
| IN35 | 0,217622 | 0,258601 |
| INADL | 0,164069 | 0,786921 |
| INF2 | 0,473677 | 0,543419 |
| INMT | 4,37E-05 | -1,01888 |
| INO1 | 1,82E-06 | 7,8255 |
| INPP | 0,0305023 | -0,8292 |
| INSR | 0,692159 | -0,101367 |
| INT6 | 0,291361 | 0,81276 |
| IP6K1 | 0,448414 | 0,277364 |
| IPO4 | 0,00661731 | 0,564898 |
| IPO5 | 7,68E-05 | 0,498819 |
| IPO7 | 0,36035 | 0,165921 |
| IPO9 | 0,0813806 | -0,645706 |
| IPYR | 0,379094 | 0,119968 |
| IPYR2 | 0,015795 | -0,711448 |
| IQGA1 | 0,216571 | 0,114573 |
| IQGA2 | 0,000109758 | 0,733695 |
| IR3IP | 0,282733 | 0,901461 |
| IREB2 | 0,0497939 | 1,13253 |
| IRGM1 | 0,00701853 | 0,401266 |
| IRGQ | 0,799519 | -0,048838 |
| IRS1 | 0,154302 | 1,14863 |
| ISC2A | 0,980033 | 0,0224991 |
| ISCA2 | 0,101934 | 0,640852 |
| ISCU | 0,30885 | -0,20122 |
| ISG15 | 0,000712018 | 2,30558 |
| ISOC1 | 0,037513 | -0,458146 |
| IST1 | 0,327256 | -0,48045 |
| ITA1 | 0,315189 | -0,212242 |
| ITA2B | 0,166437 | -0,807121 |
| ITA5 | 0,430841 | 0,396783 |
| ITAV | 0,288119 | -0,541597 |
| ITB1 | 0,725513 | 0,0434869 |
| ITB2 | 0,571332 | 0,339861 |
| ITB3 | 0,04733 | -1,31931 |
| ITFG3 | 0,185963 | -0,316961 |
| ITIH1 | 0,318037 | 0,159487 |
| ITIH2 | 0,000890871 | 0,471613 |
| ITIH3 | 0,292853 | 0,319279 |
| ITIH4 | 0,000713351 | -0,68941 |
| ITM2B | 0,700201 | 0,478244 |
| ITPA | 0,471663 | 0,250097 |
| ITPR1 | 0,136696 | -0,979493 |
| ITPR2 | 0,00785884 | -0,803842 |
| ITSN1 | 0,793998 | 0,0480426 |
| IVD | 0,0112194 | -0,469681 |
| IWS1 | 0,762133 | 0,0958389 |
| IYD1 | 0,109015 | -0,352102 |
| JAK1 | 0,589688 | -0,178226 |
| JAM1 | 0,314072 | -0,18818 |
| JIP4 | 0,789048 | -0,0685097 |
| K1161 | 0,562378 | 0,26989 |
| K1522 | 0,740105 | 0,158835 |
| K1671 | 0,999728 | -0,000355085 |
| K6PF | 0,0876589 | -0,68832 |
| K6PL | 0,00189727 | -0,480489 |
| KAD2 | 0,262436 | -0,110294 |
| KAD3 | 3,01E-07 | -1,13939 |
| KAD4 | 2,24E-06 | -5,14668 |
| KANK2 | 0,66792 | -0,128082 |
| KAP0 | 0,569258 | 0,0769288 |
| KAP2 | 0,209122 | 0,18294 |
| KAPCA | 0,155316 | -0,515195 |
| KAT1 | 4,44E-06 | -1,7475 |
| KAT3 | 7,97E-05 | -0,606264 |
| KBL | 0,13065 | -0,121204 |
| KC1A | 0,983358 | 0,00967471 |
| KC1D | 0,441193 | 0,332471 |
| KCC1A | 0,883001 | -0,083457 |
| KCC2G | 0,911515 | 0,0414998 |
| KCD12 | 0,000375745 | -0,465944 |
| KCRB | 0,958771 | 0,0141408 |
| KCY | 0,274444 | -0,531711 |
| KDSR | 0,00220792 | 0,871583 |
| KEG1 | 0,2592 | -0,183924 |
| KFA | 0,000558194 | -1,29747 |
| KGUA | 0,620129 | 0,356033 |
| KHDR1 | 0,924054 | -0,0155468 |
| KHK | 0,000100886 | -0,775563 |
| KI21A | 0,176042 | 0,965884 |
| KIBRA | 0,712164 | 0,198582 |
| KIF1C | 0,729937 | -0,307647 |
| KIF2A | 0,122882 | 0,810768 |
| KIF5A | 0,974393 | 0,032424 |
| KIME | 2,85E-06 | 4,80807 |
| KINH | 0,0551087 | 0,256868 |
| KLC1 | 0,872665 | -0,117759 |
| KLC4 | 0,0343008 | 0,42725 |
| KLDC4 | 0,0438293 | 1,1363 |
| KLKB1 | 0,190499 | -0,896331 |
| KMO | 0,000344714 | -0,870601 |
| KNG1 | 0,00781533 | -0,261548 |
| KPB2 | 0,449857 | -0,494228 |
| KPBB | 0,836254 | -0,105607 |
| KPCD2 | 0,294063 | -0,616414 |
| KPRA | 0,140674 | -0,528546 |
| KPRB | 0,795935 | 0,138161 |
| KPYM | 0,00117635 | -0,346284 |
| KPYM | 0,00164719 | -0,677969 |
| KPYR | 0,00202169 | 0,376449 |
| KS6A3 | 0,390105 | -0,390534 |
| KT3K | 0,0172682 | -1,43735 |
| KTHY | 0,125149 | -0,416258 |
| KTN1 | 0,348312 | -0,156834 |
| KYNU | 4,04E-05 | -0,998339 |
| L2GL2 | 0,764374 | -0,244283 |
| L2HDH | 0,102046 | -0,262308 |
| LA | 0,174299 | 0,348051 |
| LACB2 | 0,00125517 | 0,666852 |
| LACE1 | 0,928068 | -0,0474634 |
| LACTB | 0,00237831 | -0,809533 |
| LAGE3 | 0,39131 | -0,425874 |
| LAMA3 | 0,550167 | -0,595768 |
| LAMA5 | 0,289073 | -0,506568 |
| LAMB1 | 0,333184 | -0,589135 |
| LAMB2 | 0,643952 | -0,0987094 |
| LAMC1 | 0,0210194 | -0,804066 |
| LAMP1 | 0,752193 | -0,30546 |
| LAMP2 | 0,411671 | 0,138705 |
| LANC1 | 0,998 | 0,000568708 |
| LANC2 | 0,457601 | 0,226204 |
| LAP2A | 0,000631859 | 0,467841 |
| LAP2B | 0,13252 | 0,269383 |
| LAR4B | 0,45344 | -0,19878 |
| LARP1 | 0,170451 | -0,286274 |
| LARP4 | 0,0687585 | 0,586488 |
| LASP1 | 0,14555 | 1,00063 |
| LAT3 | 0,5104 | -1,25026 |
| LBN | 0,761257 | -0,356574 |
| LBR | 0,758301 | -0,097785 |
| LC7L2 | 0,0319515 | -0,406257 |
| LC7L3 | 0,328905 | -0,711637 |
| LCAP | 0,208087 | -0,307327 |
| LCAT | 0,821843 | -0,0778481 |
| LCLT1 | 0,113731 | 0,352852 |
| LDHA | 0,410104 | 0,105682 |
| LDHB | 5,99E-07 | 2,72259 |
| LDHD | 2,63E-06 | 1,09511 |
| LDLR | 0,0336966 | 0,461366 |
| LEG1 | 0,00297601 | 1,08806 |
| LEG3 | 0,836329 | 0,128049 |
| LEG8 | 0,0179065 | 0,73641 |
| LEG9 | 0,241296 | 0,497795 |
| LEGL | 0,000136541 | -0,664663 |
| LEMD2 | 0,231611 | 0,596165 |
| LETM1 | 0,0561115 | 0,282326 |
| LG3BP | 0,1987 | 0,237515 |
| LGMN | 0,320561 | 0,432589 |
| LGUL | 0,00834781 | -0,586172 |
| LHPP | 1,69E-06 | 3,39832 |
| LIAS | 0,418912 | 0,454844 |
| LICH | 0,04166 | 2,31537 |
| LIFR | 0,287267 | 0,645017 |
| LIMA1 | 0,734078 | 0,0778039 |
| LIMS1 | 0,0684315 | 0,275413 |
| LIMS2 | 0,153138 | -1,00744 |
| LIN7A | 5,27E-05 | -1,10189 |
| LIN7C | 0,379136 | 0,223845 |
| LIPB1 | 0,291364 | 0,881348 |
| LIPB2 | 0,0126282 | 0,672001 |
| LIPC | 0,867417 | 0,0889556 |
| LIPR1 | 0,0138235 | 2,18392 |
| LIPT2 | 0,58363 | -0,321736 |
| LIS1 | 0,0170192 | 0,483034 |
| LKHA4 | 0,182054 | -0,144656 |
| LMA2L | 0,619179 | -0,426654 |
| LMAN1 | 0,0296597 | -0,313142 |
| LMAN2 | 0,576064 | -0,0543264 |
| LMF1 | 0,0138879 | -1,69215 |
| LMNA | 0,0650606 | 0,19425 |
| LMNB1 | 0,0815666 | -0,297971 |
| LMNB2 | 0,00489609 | -0,702418 |
| LONM | 0,00358631 | -0,323004 |
| LONP2 | 0,00112073 | 1,03008 |
| LPHN2 | 0,604465 | 0,455553 |
| LPIN2 | 0,437742 | 0,544489 |
| LPP | 0,943495 | 0,00687726 |
| LPP3 | 0,0241037 | 0,404052 |
| LPP60 | 2,96E-06 | -2,9116 |
| LPPRC | 0,0528839 | -0,309817 |
| LRBA | 0,0101025 | 1,21491 |
| LRC40 | 0,512617 | 0,383127 |
| LRC47 | 0,0786102 | 0,302836 |
| LRC57 | 0,269283 | -0,641345 |
| LRC58 | 0,042628 | 1,12274 |
| LRC59 | 0,571322 | 0,101924 |
| LRC8D | 0,116209 | 0,389056 |
| LRP1 | 0,403137 | -0,0724344 |
| LRRF1 | 0,952677 | -0,0463721 |
| LRRF2 | 0,196049 | 0,797866 |
| LSM12 | 0,36147 | 0,229281 |
| LSM2 | 0,294676 | 0,461206 |
| LSM3 | 0,165638 | -1,24915 |
| LSM4 | 0,0343207 | -0,920784 |
| LSM8 | 0,106983 | -0,262785 |
| LSP1 | 0,0395518 | 2,28724 |
| LSR | 0,093956 | -0,37726 |
| LST8 | 0,986424 | -0,0038166 |
| LTMD1 | 0,123524 | -0,365393 |
| LTN1 | 0,376669 | 0,43339 |
| LTOR1 | 0,0214802 | 0,889891 |
| LTOR2 | 0,416343 | -0,218259 |
| LTOR3 | 0,499119 | 0,427803 |
| LUC7L | 0,853394 | -0,197415 |
| LXN | 0,375762 | -0,531468 |
| LYAG | 0,703836 | -0,0803706 |
| LYN | 0,0199485 | -0,639616 |
| LYPA1 | 0,985163 | -0,00373205 |
| LYPA2 | 0,811023 | -0,0582167 |
| LYPL1 | 0,656798 | -0,240782 |
| LYRIC | 0,44008 | -0,144582 |
| LYRM4 | 0,0169364 | -0,802976 |
| LYRM5 | 0,334422 | -0,904468 |
| LYZ1 | 0,0204688 | -1,2713 |
| LYZ2 | 0,920003 | -0,0515661 |
| LZIC | 0,692158 | 0,472044 |
| M2GD | 0,0134598 | -0,271876 |
| M2OM | 0,00332625 | -0,709444 |
| M4K5 | 0,198888 | -0,678894 |
| MA1A1 | 0,308967 | 0,348279 |
| MA1B1 | 0,0751688 | -0,244707 |
| MA2A1 | 0,648535 | -0,0582231 |
| MA2B1 | 5,76E-05 | -1,32178 |
| MA2B2 | 0,135581 | -0,758862 |
| MA2C1 | 0,41826 | -0,200517 |
| MA7D1 | 0,726702 | -0,0892928 |
| MAAI | 0,052993 | -0,16954 |
| MACD1 | 0,000965598 | -0,536194 |
| MACF1 | 0,600988 | 0,079484 |
| MAEA | 0,301498 | -0,655178 |
| MAGI1 | 0,389955 | -0,956172 |
| MAGI3 | 0,435854 | 0,459706 |
| MAGT1 | 0,193098 | 0,934731 |
| MANBA | 0,0414026 | 1,42304 |
| MANF | 0,889717 | 0,0161492 |
| MAOM | 0,478838 | 0,423237 |
| MAOX | 3,76E-05 | 2,25782 |
| MAP11 | 0,137133 | 0,305904 |
| MAP1S | 0,793331 | 0,103011 |
| MAP2 | 0,0730603 | 0,733825 |
| MAP4 | 0,405875 | -0,0728048 |
| MAPK2 | 0,0067476 | 0,599676 |
| MARE1 | 0,605943 | 0,164431 |
| MARE3 | 0,395397 | 0,485848 |
| MARF1 | 0,870788 | 0,0628093 |
| MARH5 | 0,895566 | 0,0747795 |
| MARH6 | 0,581113 | -0,400181 |
| MARK2 | 0,995397 | 0,00157038 |
| MARK3 | 0,332327 | 0,199833 |
| MASP1 | 0,125696 | -0,720596 |
| MAT2B | 0,314296 | 0,17787 |
| MATR3 | 0,77334 | -0,0263596 |
| MAVS | 0,776458 | -0,101896 |
| MB12A | 0,0513612 | -0,830487 |
| MBB1A | 0,00997128 | 0,404476 |
| MBD2 | 0,0323553 | 1,00527 |
| MBL1 | 0,520898 | -0,176267 |
| MBL2 | 0,324384 | -0,235372 |
| MBNL1 | 0,0572558 | -1,13127 |
| MBNL2 | 0,28731 | -0,623158 |
| MBOA5 | 0,717793 | 0,219965 |
| MBOA7 | 0,860801 | -0,121833 |
| MCAT | 0,506685 | -0,145267 |
| MCCA | 5,47E-06 | -0,752528 |
| MCCB | 1,18E-06 | -0,749512 |
| MCEE | 0,364553 | -0,570335 |
| MCES | 0,969461 | -0,0211372 |
| MCM2 | 0,156205 | 0,334975 |
| MCM3 | 0,270583 | 0,396268 |
| MCM4 | 0,0993892 | 0,500024 |
| MCM5 | 0,126041 | 0,485199 |
| MCM6 | 0,00690622 | 0,591605 |
| MCM7 | 0,0222578 | 0,703386 |
| MCMBP | 0,118286 | 0,581687 |
| MCTS1 | 0,475596 | -0,15104 |
| MCU | 0,294359 | 1,15365 |
| MD1L1 | 0,0165366 | -3,06373 |
| MD2L1 | 0,258437 | -0,625751 |
| MDHC | 0,00775653 | -0,396663 |
| MDHM | 0,304383 | 0,160203 |
| MDR1A | 0,433627 | 0,434587 |
| MDR3 | 0,0143127 | 0,829536 |
| MECP2 | 0,139857 | -0,688578 |
| MECR | 0,00535655 | -0,616928 |
| MEMO1 | 0,0196257 | -0,447494 |
| MEP50 | 0,28191 | 0,383494 |
| MESD | 0,134651 | -0,399704 |
| MESH1 | 0,037662 | -0,690701 |
| MET7B | 0,422675 | 0,253222 |
| METK1 | 0,00833091 | -0,385347 |
| METK2 | 2,80E-06 | 1,80238 |
| MFAP1 | 0,301077 | -0,182093 |
| MFN1 | 0,243968 | -0,111771 |
| MFN2 | 0,406069 | -0,164521 |
| MFR1L | 0,0632216 | -1,6099 |
| MGAT2 | 0,63175 | -0,489601 |
| MGDP1 | 0,484736 | 0,14598 |
| MGLL | 0,00083689 | -0,579641 |
| MGN | 0,96026 | 0,00506147 |
| MGST1 | 0,191593 | 0,307212 |
| MI4GD | 0,160818 | 1,4944 |
| MIA3 | 0,0623576 | 0,32398 |
| MIB1 | 0,7683 | 0,220353 |
| MIEN1 | 0,0802517 | 1,30425 |
| MIF | 0,483681 | -0,235978 |
| MIMIT | 0,921977 | -0,0184367 |
| MIO | 0,0557157 | -0,424393 |
| MIP18 | 0,0487089 | -1,29897 |
| MIPEP | 0,713228 | -0,0784483 |
| MIRO1 | 0,115023 | -0,279472 |
| MIRO2 | 0,479911 | -0,692549 |
| MK01 | 0,324694 | 0,122822 |
| MK03 | 0,273375 | 0,338809 |
| MK09 | 0,644534 | -0,0938196 |
| MK14 | 0,610125 | 0,0695254 |
| ML12B | 0,361758 | 0,314312 |
| MLEC | 0,0662445 | -0,238845 |
| MLKL | 0,693001 | 0,191969 |
| MLP3B | 0,262301 | -0,55413 |
| MMAA | 0,0194023 | 0,387955 |
| MMAB | 0,0424465 | 0,655615 |
| MMS19 | 0,601401 | 0,3785 |
| MMSA | 0,000434751 | -0,433562 |
| MNDAL | 0,209797 | -0,36876 |
| MOB1B | 0,183653 | 0,855862 |
| MOB2 | 0,68641 | -0,19154 |
| MOC2A | 0,772618 | -0,075607 |
| MOC2B | 0,486271 | -0,298296 |
| MOCOS | 3,14E-08 | 3,32497 |
| MOCS1 | 0,930027 | -0,0155986 |
| MOCS3 | 0,08955 | 1,39149 |
| MOES | 0,751874 | -0,0361083 |
| MOGS | 0,00639154 | -0,563275 |
| MON2 | 0,538605 | 0,151277 |
| MOSC1 | 0,191364 | -0,214125 |
| MOSC2 | 8,69E-07 | 0,616647 |
| MOT1 | 0,29186 | 0,314972 |
| MOV10 | 0,000210858 | -0,776741 |
| MP2K1 | 0,424894 | 0,533278 |
| MP2K2 | 0,860785 | -0,0283429 |
| MP2K3 | 0,641521 | -0,111088 |
| MP2K4 | 0,420486 | -0,424648 |
| MP2K6 | 0,00341666 | -1,30966 |
| MPC1 | 0,477689 | -0,630002 |
| MPC2 | 0,0227447 | -0,980706 |
| MPCP | 0,756986 | -0,0778726 |
| MPI | 0,0285482 | 2,42658 |
| MPP6 | 0,270686 | -0,469866 |
| MPPA | 0,166742 | -0,228889 |
| MPPB | 0,00393723 | -0,449715 |
| MPRD | 0,327262 | 0,462794 |
| MPRI | 0,00999557 | 0,438558 |
| MPRIP | 0,994566 | 0,00221666 |
| MPU1 | 0,585422 | -0,336987 |
| MRC1 | 0,0278175 | -0,41497 |
| MRCKB | 0,219697 | 0,133109 |
| MRE11 | 0,14483 | -0,442778 |
| MRP2 | 0,00034712 | 1,03861 |
| MRP3 | 0,686392 | -0,0908499 |
| MRP6 | 0,0968456 | -0,278319 |
| MRRP1 | 0,705419 | 0,172908 |
| MSH2 | 0,0290596 | -1,0208 |
| MSI2H | 0,540909 | -0,197053 |
| MSMO1 | 1,38E-07 | 4,79432 |
| MSPD2 | 0,995809 | 0,0026261 |
| MSRA | 0,198923 | -0,32713 |
| MSRB2 | 0,0596802 | -0,719963 |
| MTA1 | 0,663256 | 0,246505 |
| MTA2 | 0,272097 | 0,163746 |
| MTA3 | 0,733597 | -0,181257 |
| MTAP | 0,838 | -0,0527465 |
| MTCH1 | 0,719323 | -0,130765 |
| MTCH2 | 0,587353 | -0,146006 |
| MTFP1 | 0,421873 | -0,422023 |
| MTHFS | 0,00904315 | -0,958833 |
| MTM1 | 0,412529 | -0,24702 |
| MTMR5 | 0,665508 | -0,270504 |
| MTNA | 0,0198557 | 0,268977 |
| MTNB | 0,598567 | -0,0986404 |
| MTND | 0,110415 | -0,231779 |
| MTOR | 0,159664 | 0,394751 |
| MTP | 0,906411 | -0,00870577 |
| MTPN | 0,707768 | 0,0576824 |
| MTSS1 | 0,173353 | -0,664459 |
| MTU1 | 0,0948216 | -1,40225 |
| MTUS1 | 0,370387 | 0,212708 |
| MTX1 | 0,493191 | 0,150945 |
| MTX2 | 0,883944 | -0,0437469 |
| MUC18 | 0,0308213 | 0,341004 |
| MUG1 | 0,0167631 | -1,43552 |
| MUG2 | 0,772299 | -0,286806 |
| MUTA | 0,255281 | 0,132526 |
| MVD1 | 0,000861764 | 3,85789 |
| MVP | 2,31E-05 | 0,80834 |
| MY18A | 0,390834 | 0,117676 |
| MYCBP | 0,329474 | -0,231105 |
| MYEF2 | 0,131491 | -0,434467 |
| MYG1 | 0,301015 | 0,185344 |
| MYH10 | 0,0105002 | 0,559196 |
| MYH11 | 0,879055 | 0,08616 |
| MYH14 | 0,0307645 | 0,723579 |
| MYH6 | 0,00210382 | 2,89431 |
| MYH9 | 0,945218 | -0,0105508 |
| MYL6 | 0,704206 | 0,0410706 |
| MYL9 | 0,909264 | -0,0599995 |
| MYLK | 0,906479 | 0,012653 |
| MYO16 | 0,0220583 | -1,68799 |
| MYO1B | 0,324368 | 0,187636 |
| MYO1C | 0,0935838 | -0,19575 |
| MYO1C | 0,312014 | -0,461182 |
| MYO1D | 0,349157 | 0,381675 |
| MYO1E | 0,695618 | 0,0674375 |
| MYO5B | 0,0501676 | 1,70298 |
| MYO6 | 0,0154279 | 0,499324 |
| MYPT1 | 0,521887 | 0,237112 |
| NAA10 | 0,717082 | -0,118352 |
| NAA15 | 0,651783 | 0,0589317 |
| NAA16 | 0,734711 | 0,215231 |
| NAA20 | 0,117266 | 0,912387 |
| NAA25 | 0,111777 | 0,975145 |
| NAA30 | 0,535971 | -0,134268 |
| NAA35 | 0,857997 | -0,0829509 |
| NAA50 | 0,698666 | 0,0728124 |
| NACA | 0,210163 | 0,216105 |
| NACAD | 0,952611 | -0,0612208 |
| NADC | 0,0419369 | -0,258952 |
| NADE | 0,272443 | -0,243634 |
| NADK | 0,0387559 | 0,526324 |
| NAGA | 0,146376 | 0,339577 |
| NAGAB | 0,08421 | -0,35052 |
| NAGK | 0,00990946 | 0,438163 |
| NAGPA | 0,801318 | 0,143352 |
| NAGS | 3,98E-05 | -1,15531 |
| NAKD2 | 7,58E-05 | -0,434329 |
| NAKD2 | 0,148947 | -0,887492 |
| NAMPT | 0,38042 | 0,136153 |
| NANP | 0,655343 | -0,189081 |
| NARFL | 0,238657 | -0,688496 |
| NASP | 0,876884 | -0,0638606 |
| NB5R3 | 0,0580744 | 0,218252 |
| NCALD | 0,0940647 | 1,71378 |
| NCBP1 | 0,550265 | -0,342618 |
| NCBP2 | 0,278964 | 0,744327 |
| NCEH1 | 0,0186051 | 0,496769 |
| NCK1 | 0,78231 | -0,0764093 |
| NCKP1 | 0,0240541 | 0,445936 |
| NCLN | 0,58525 | 0,139655 |
| NCOA5 | 0,117635 | 0,709852 |
| NCOAT | 0,0257002 | 1,7589 |
| NCPR | 1,90E-05 | 0,885153 |
| NDK3 | 0,0035296 | -0,795344 |
| NDKA | 0,07543 | -0,29682 |
| NDKB | 0,850593 | 0,0910165 |
| NDRG1 | 5,10E-06 | 1,82675 |
| NDRG2 | 0,00346941 | -0,442458 |
| NDST1 | 0,64004 | 0,518931 |
| NDUA2 | 0,254998 | -0,517048 |
| NDUA4 | 0,0306684 | -0,646565 |
| NDUA5 | 0,00844211 | -0,793216 |
| NDUA6 | 0,00495622 | -0,780703 |
| NDUA7 | 0,307343 | -0,357942 |
| NDUA8 | 0,426932 | -0,228888 |
| NDUA9 | 0,0163564 | -0,768014 |
| NDUAA | 0,0404913 | -0,460054 |
| NDUAB | 0,0736134 | -0,611667 |
| NDUAC | 0,0143555 | -0,435662 |
| NDUAD | 0,0106612 | -0,486049 |
| NDUB2 | 0,0405871 | -1,13231 |
| NDUB3 | 0,00673635 | -0,50607 |
| NDUB4 | 0,0184023 | -0,648329 |
| NDUB5 | 0,147905 | -0,390126 |
| NDUB6 | 0,156567 | -1,12543 |
| NDUB7 | 0,0192794 | -0,581081 |
| NDUB8 | 0,0187193 | -0,530734 |
| NDUB9 | 0,0124664 | -0,632797 |
| NDUBA | 0,286569 | -0,190833 |
| NDUBB | 0,000179934 | -1,03021 |
| NDUC2 | 0,167705 | -0,626317 |
| NDUF4 | 0,386932 | -0,504019 |
| NDUF6 | 0,187074 | -1,1449 |
| NDUS1 | 0,0174925 | -0,392203 |
| NDUS2 | 0,0194203 | -0,478463 |
| NDUS3 | 0,007815 | -0,490325 |
| NDUS4 | 0,675036 | -0,124761 |
| NDUS5 | 0,354009 | -0,336041 |
| NDUS6 | 0,221035 | -0,39196 |
| NDUS7 | 0,00646841 | -0,465369 |
| NDUS8 | 0,194878 | -0,271959 |
| NDUV1 | 0,0627031 | -0,40327 |
| NDUV2 | 0,00799484 | -0,519557 |
| NECP1 | 0,756531 | -0,331717 |
| NEDD4 | 0,0289595 | 0,405581 |
| NEDD8 | 0,885377 | 0,110317 |
| NEK7 | 0,403725 | 0,325819 |
| NEK9 | 0,00428379 | 1,33285 |
| NELFB | 0,396912 | -0,621463 |
| NELFD | 0,807452 | 0,112036 |
| NEMF | 0,979578 | 0,00727081 |
| NEUA | 0,244836 | 0,243265 |
| NEUL | 0,0291653 | 0,249358 |
| NFIA | 0,037399 | -0,799099 |
| NFIB | 0,0376802 | -0,539102 |
| NFIC | 0,662791 | 0,296034 |
| NFKB1 | 0,940416 | -0,0316289 |
| NFS1 | 0,728169 | 0,0662187 |
| NFU1 | 0,784964 | -0,0482391 |
| NGLY1 | 0,521418 | 0,370598 |
| NH2L1 | 0,444166 | -0,14951 |
| NHLC2 | 0,0330736 | 1,38616 |
| NHP2 | 0,264242 | 0,611272 |
| NHRF1 | 0,840233 | 0,0375532 |
| NHRF2 | 0,0137755 | -0,796149 |
| NHRF3 | 3,41E-05 | 0,726887 |
| NIBL1 | 0,24493 | 0,681843 |
| NICA | 0,6123 | -0,177474 |
| NID1 | 0,520025 | -0,231475 |
| NID2 | 0,681886 | 0,133403 |
| NIPS1 | 0,049646 | -0,348335 |
| NIPS2 | 0,0184815 | -2,13467 |
| NISCH | 0,662392 | 0,291387 |
| NIT1 | 0,5887 | 0,0706584 |
| NIT2 | 0,0109681 | -0,393577 |
| NJMU | 0,0454724 | 1,09136 |
| NLRX1 | 0,00259124 | -1,502 |
| NLTP | 1,50E-05 | 0,943616 |
| NMI | 0,00556246 | 0,436802 |
| NMNA1 | 0,0953314 | -0,649017 |
| NMNA3 | 0,326354 | -0,65419 |
| NMRL1 | 0,761306 | -0,0806338 |
| NMT1 | 0,00206852 | 0,487281 |
| NMT2 | 0,0155243 | 0,51836 |
| NNRD | 0,81732 | -0,138771 |
| NNRD | 0,00618012 | -0,651309 |
| NNRE | 0,012103 | -0,55768 |
| NNTM | 0,42664 | 2,1749 |
| NOG1 | 0,111325 | 0,71757 |
| NOMO1 | 0,108324 | 0,15622 |
| NONO | 0,663962 | -0,048206 |
| NOP2 | 0,225613 | 1,00669 |
| NOP56 | 0,405883 | -0,176963 |
| NOP58 | 0,475938 | 0,173274 |
| NP1L1 | 0,272374 | 0,551389 |
| NP1L4 | 0,319649 | 0,203093 |
| NPC1 | 0,223917 | 0,875603 |
| NPC2 | 0,0720128 | -0,422626 |
| NPL | 0,279293 | -0,260459 |
| NPL4 | 0,0234801 | 0,42228 |
| NPM | 0,559627 | 0,543506 |
| NPS3B | 0,171952 | -0,411581 |
| NPTN | 0,257219 | 0,556276 |
| NQO1 | 0,000404832 | 2,52176 |
| NQO2 | 0,562887 | 0,263001 |
| NRBP | 0,312017 | 0,367632 |
| NRDC | 0,545674 | 0,11762 |
| NSDHL | 7,73E-07 | 4,64586 |
| NSF | 0,634166 | -0,0501293 |
| NSF1C | 0,000890791 | 0,607735 |
| NSMA | 1,70E-05 | 1,3829 |
| NSUN2 | 0,0195468 | 0,308409 |
| NT5C | 0,54148 | 0,19536 |
| NT5D1 | 0,00638088 | 1,29799 |
| NTF2 | 0,827966 | 0,0816727 |
| NU107 | 0,296521 | 0,87326 |
| NU133 | 0,190993 | 0,981813 |
| NU155 | 0,568927 | -0,107615 |
| NU160 | 0,0574966 | 1,62566 |
| NU214 | 0,0376441 | -0,440434 |
| NU4M | 0,697623 | -0,344689 |
| NUB1 | 0,068868 | 0,906024 |
| NUBP1 | 0,231858 | 0,644859 |
| NUBP2 | 0,84326 | 0,0913617 |
| NUBPL | 0,167954 | 1,04801 |
| NUCB1 | 0,877975 | -0,0225093 |
| NUCB2 | 0,132159 | -0,308745 |
| NUCG | 1,72E-07 | -1,51155 |
| NUCL | 0,309209 | 0,163075 |
| NUD12 | 0,000232103 | 0,780938 |
| NUD14 | 0,759982 | 0,197168 |
| NUD16 | 0,00777482 | -1,33899 |
| NUD19 | 0,0104285 | -0,856424 |
| NUDC | 0,176449 | 0,211761 |
| NUDC1 | 0,982963 | 0,014568 |
| NUDC2 | 0,222502 | 0,345722 |
| NUDT5 | 0,0880644 | -0,541216 |
| NUDT7 | 0,000802393 | 0,699394 |
| NUDT9 | 0,637113 | 0,178745 |
| NUFP2 | 0,981058 | 0,00560951 |
| NUMB | 0,11214 | -0,260194 |
| NUP37 | 0,153249 | -0,908282 |
| NUP43 | 0,210809 | 0,695137 |
| NUP50 | 0,45678 | 0,459133 |
| NUP53 | 0,524398 | -0,225182 |
| NUP54 | 0,667712 | 0,10911 |
| NUP62 | 0,648297 | 0,183165 |
| NUP88 | 0,699743 | 0,112548 |
| NUP93 | 0,979793 | -0,00685056 |
| NUP98 | 0,394782 | 0,560649 |
| NUPL1 | 0,803003 | 0,11666 |
| NXF1 | 0,684843 | -0,139505 |
| NXP20 | 0,120179 | 0,353317 |
| OARD1 | 0,522885 | -0,31021 |
| OAS1A | 0,0932519 | 0,668167 |
| OASL1 | 0,00920423 | 1,7995 |
| OAT | 2,51E-13 | 5,8653 |
| OCAD1 | 0,395119 | -0,175517 |
| OCAD2 | 0,00391398 | -2,9616 |
| OCTC | 2,18E-07 | 0,945162 |
| ODB2 | 0,0196152 | -0,590082 |
| ODBA | 0,000758151 | -0,422664 |
| ODBB | 0,0201138 | -0,323415 |
| ODC | 5,33E-07 | 1,24232 |
| ODO1 | 0,0135483 | -0,245131 |
| ODO2 | 0,00042491 | -0,506465 |
| ODP2 | 0,50158 | 0,1034 |
| ODPA | 0,0557196 | 0,364866 |
| ODPB | 0,000135049 | 0,409761 |
| ODPX | 0,919284 | 0,0281677 |
| ODR4 | 0,742101 | -0,0751553 |
| OFUT1 | 0,322451 | 0,469713 |
| OGFD3 | 0,0855032 | 1,20535 |
| OGFR | 0,685909 | 0,18953 |
| OGT1 | 0,382487 | 0,236329 |
| OLA1 | 0,316261 | -0,120961 |
| OMA1 | 0,0565294 | 0,875983 |
| OPA1 | 0,00110714 | -0,429237 |
| OPLA | 0,000343625 | 0,646349 |
| OPTN | 0,964766 | -0,058596 |
| ORC1 | 0,530657 | -0,605637 |
| ORN | 0,0372899 | 2,0404 |
| ORNT1 | 0,0598027 | -0,47324 |
| OSB11 | 0,621571 | -0,265822 |
| OSBL1 | 0,959663 | -0,0286919 |
| OSBL9 | 0,990181 | -0,00256125 |
| OSBP1 | 0,0574866 | 0,393628 |
| OSGEP | 0,231247 | -0,312106 |
| OST48 | 0,00100511 | -0,533402 |
| OSTC | 0,40412 | -1,14721 |
| OSTF1 | 0,494369 | -0,314135 |
| OTC | 1,47E-07 | -1,58479 |
| OTU6B | 0,827473 | -0,133388 |
| OTUB1 | 0,480664 | 0,0898174 |
| OTUD4 | 0,340714 | -0,597189 |
| OXA1L | 0,448366 | 0,505447 |
| OXND1 | 0,511283 | -0,212421 |
| OXR1 | 0,254166 | 0,239174 |
| OXSM | 0,00261324 | -0,487861 |
| OXSR1 | 0,708573 | -0,0573219 |
| P20D1 | 0,942304 | -0,0187511 |
| P2RX4 | 0,81438 | 0,13426 |
| P4HA1 | 0,00406018 | 1,50816 |
| P4K2A | 0,934514 | -0,0480502 |
| P4R3A | 0,891036 | 0,038249 |
| P4R3B | 0,30113 | 1,0065 |
| P5CR3 | 0,341692 | 0,17182 |
| P5CS | 0,0234834 | 1,44024 |
| P66A | 0,64186 | 0,324387 |
| P66B | 0,967077 | -0,0384576 |
| PA1B2 | 0,462287 | 0,611357 |
| PA2G4 | 0,241345 | -0,14496 |
| PABP1 | 0,657586 | 0,0706743 |
| PABP2 | 0,480109 | -0,104905 |
| PACN2 | 0,370586 | -0,230482 |
| PACN3 | 0,000862928 | 0,728409 |
| PAFA2 | 0,988951 | -0,00234445 |
| PAG15 | 0,263119 | -0,552967 |
| PAHX | 1,11E-05 | 1,28124 |
| PAIP1 | 0,825598 | 0,0526311 |
| PAIRB | 0,776816 | -0,0293973 |
| PAK2 | 0,246519 | -0,323088 |
| PAK4 | 0,642771 | 0,207973 |
| PALD | 0,0620991 | -1,02541 |
| PALLD | 0,0763436 | -1,68691 |
| PALMD | 3,69E-05 | 1,64489 |
| PANK1 | 0,00171831 | 1,08637 |
| PANK4 | 0,0670089 | 0,72131 |
| PAPD1 | 0,128718 | -0,36857 |
| PAPS1 | 0,121295 | -0,60145 |
| PAPS2 | 0,108572 | 0,173491 |
| PAQR9 | 0,332581 | -0,871168 |
| PAR12 | 0,0436407 | 0,392391 |
| PAR14 | 0,433094 | -0,258772 |
| PARD3 | 0,106831 | 0,49691 |
| PARK7 | 0,212334 | 0,2193 |
| PARP1 | 0,222226 | -0,167639 |
| PARP9 | 0,00209237 | 0,608968 |
| PARVA | 0,821309 | 0,0819756 |
| PAWR | 0,17428 | 0,606392 |
| PAXB1 | 0,124187 | 0,429123 |
| PAXI | 0,332848 | -0,25086 |
| PBDC1 | 0,566713 | -0,390456 |
| PBLD1 | 0,000115663 | -0,886933 |
| PBLD2 | 2,92E-05 | -1,33293 |
| PCBP1 | 0,0434323 | -0,396961 |
| PCBP2 | 0,252392 | 0,344285 |
| PCCA | 8,96E-09 | -1,0939 |
| PCCB | 1,73E-09 | -1,1192 |
| PCID2 | 0,442581 | -0,519431 |
| PCKGC | 0,000108783 | -0,888425 |
| PCKGM | 0,0494865 | 1,12757 |
| PCNA | 0,387128 | 0,221573 |
| PCNP | 0,518334 | 0,285055 |
| PCP | 0,039444 | -1,23005 |
| PCTL | 0,0747189 | 0,317878 |
| PCY1A | 0,00236489 | 0,638104 |
| PCY2 | 0,00126452 | 0,759832 |
| PCYOX | 0,145234 | -0,163433 |
| PDC10 | 0,243023 | -0,301455 |
| PDC6I | 0,305686 | -0,14492 |
| PDCD4 | 0,000710454 | -2,46691 |
| PDCD5 | 0,322476 | -0,222151 |
| PDCD6 | 0,841594 | 0,0367908 |
| PDCL3 | 0,733015 | -0,272529 |
| PDDC1 | 0,28128 | -0,350957 |
| PDE12 | 0,200779 | -0,311133 |
| PDE2A | 0,00111373 | -1,68735 |
| PDE3B | 0,54567 | -0,410211 |
| PDIA1 | 0,535138 | 0,0657549 |
| PDIA3 | 0,00418011 | -0,264414 |
| PDIA4 | 0,190061 | 0,126416 |
| PDIA5 | 0,00111477 | -0,532048 |
| PDIA6 | 0,933131 | -0,0164897 |
| PDIP2 | 0,0935982 | 0,152968 |
| PDIP3 | 0,832802 | -0,0743732 |
| PDK1 | 0,127508 | -0,371665 |
| PDK2 | 0,000688129 | -0,483006 |
| PDK4 | 0,000570402 | 2,95881 |
| PDLI1 | 0,169557 | -0,214124 |
| PDLI5 | 0,801998 | 0,0292568 |
| PDLI7 | 0,386812 | 0,600978 |
| PDS5A | 0,746985 | -0,0571515 |
| PDS5B | 0,475171 | -0,237503 |
| PDXD1 | 0,748536 | -0,0470209 |
| PDXK | 0,00402268 | 0,608652 |
| PEA15 | 0,423889 | -0,908704 |
| PEBP1 | 0,0151008 | 0,621567 |
| PECR | 4,62E-05 | -1,10703 |
| PEDF | 0,0076227 | -1,11739 |
| PEF1 | 0,905314 | -0,0568377 |
| PELO | 0,146058 | -0,477357 |
| PEPD | 0,0241267 | -0,626355 |
| PEPL | 0,00261007 | 2,61847 |
| PEPL1 | 0,216271 | -0,1813 |
| PERQ2 | 0,959427 | 0,0117998 |
| PEX1 | 0,135073 | 0,216114 |
| PEX14 | 0,117192 | 0,421558 |
| PEX16 | 0,434452 | 0,295061 |
| PEX19 | 0,00286976 | 0,804658 |
| PEX26 | 0,129095 | 0,543974 |
| PEX3 | 0,0148333 | 0,370649 |
| PEX5 | 0,0525971 | 0,358116 |
| PEX6 | 0,435933 | 0,137179 |
| PEX7 | 0,635448 | -0,272213 |
| PFD1 | 0,28481 | 1,03416 |
| PFD2 | 0,417245 | -0,486924 |
| PFD3 | 0,99347 | 0,00518068 |
| PFD5 | 0,264786 | -0,352235 |
| PFD6 | 0,471323 | 0,129927 |
| PG12B | 0,0459182 | -0,882367 |
| PGAM1 | 0,178905 | -0,227511 |
| PGAM5 | 0,968626 | 0,0212186 |
| PGAP1 | 0,01355 | -0,693986 |
| PGBM | 0,152736 | 0,166773 |
| PGDH | 0,02142 | 0,662804 |
| PGES2 | 0,562095 | -0,146516 |
| PGH1 | 0,00941835 | -0,628919 |
| PGK1 | 0,116555 | -0,239004 |
| PGM1 | 0,100039 | -0,178934 |
| PGM2 | 0,00268492 | -0,700911 |
| PGP | 0,190377 | 0,244785 |
| PGRC1 | 0,00297814 | 0,469987 |
| PGRC2 | 0,994629 | 0,00107924 |
| PGS1 | 0,596074 | 0,241829 |
| PGS2 | 0,0180742 | -0,554811 |
| PGTA | 0,690576 | -0,0862201 |
| PGTB2 | 0,0751597 | 0,717867 |
| PH4H | 0,000400301 | 0,645815 |
| PHAR4 | 0,797537 | 0,134343 |
| PHB | 0,00117028 | -0,556785 |
| PHB2 | 0,00188779 | -0,497107 |
| PHF5A | 0,00120244 | 0,605947 |
| PHKG2 | 0,536351 | -0,483458 |
| PHLB2 | 0,427502 | 0,431372 |
| PHLD | 0,0547225 | 1,86001 |
| PHLP | 0,279342 | 0,456592 |
| PHOCN | 0,639016 | 0,347721 |
| PHS | 0,788314 | 0,0395759 |
| PHS2 | 0,458709 | 0,371056 |
| PHYD1 | 0,0128365 | -0,59188 |
| PI3R4 | 0,189772 | 0,827723 |
| PI42C | 0,026304 | 0,839119 |
| PICAL | 0,0883115 | -0,474597 |
| PIGS | 0,240298 | 0,806416 |
| PIGU | 0,988638 | -0,0111192 |
| PIMT | 0,0532311 | -0,365687 |
| PIN1 | 0,21942 | 0,77215 |
| PIN4 | 0,855231 | 0,0354379 |
| PININ | 0,774952 | -0,175046 |
| PIPNA | 0,0615381 | -0,308768 |
| PIPNB | 0,466939 | -0,111145 |
| PIR | 0,00483498 | 1,09753 |
| PISD | 0,0969295 | -0,370255 |
| PITC1 | 0,0250536 | 0,74837 |
| PJA1 | 0,850019 | 0,0689338 |
| PK1L2 | 0,00609206 | 1,71768 |
| PK3CA | 0,0905397 | 0,751576 |
| PKHA6 | 0,372087 | -0,5051 |
| PKHA7 | 0,509373 | 0,534851 |
| PKHO2 | 0,159076 | -0,773801 |
| PKN2 | 0,549784 | -0,298263 |
| PLAK | 0,000221453 | -0,888487 |
| PLAP | 0,0310502 | 0,224742 |
| PLBL1 | 0,0135469 | 0,668927 |
| PLBL2 | 0,335722 | -0,362088 |
| PLCB | 0,0755932 | -0,408331 |
| PLCB1 | 0,368662 | 0,436821 |
| PLCB3 | 0,65811 | -0,18919 |
| PLCC | 0,207827 | 0,458716 |
| PLCG1 | 0,0759786 | 1,05247 |
| PLD3 | 0,108526 | 0,837941 |
| PLD4 | 0,00162103 | -1,78268 |
| PLEC | 0,861367 | 0,019901 |
| PLEK | 0,561522 | 0,32462 |
| PLF4 | 0,744155 | -0,125871 |
| PLIN2 | 0,134423 | -0,284538 |
| PLIN3 | 0,0203003 | 0,269287 |
| PLIN5 | 2,96E-06 | 2,67418 |
| PLMN | 0,0128903 | -0,401925 |
| PLOD1 | 0,669136 | 0,330278 |
| PLOD3 | 0,84849 | 0,182595 |
| PLPL8 | 0,818357 | 0,0573432 |
| PLPL9 | 8,80E-06 | 2,2246 |
| PLRG1 | 0,437941 | -0,25253 |
| PLS1 | 0,0705156 | 0,533626 |
| PLS3 | 0,110717 | -0,435997 |
| PLSI | 0,453798 | 0,431055 |
| PLSL | 0,00754707 | 0,236797 |
| PLST | 5,44E-08 | -1,87555 |
| PLVAP | 0,0501104 | -1,62651 |
| PLXB1 | 0,288347 | 0,543676 |
| PLXB2 | 0,894921 | -0,0285288 |
| PM14 | 0,999579 | 0,000289917 |
| PMGE | 0,362832 | 0,988555 |
| PML | 0,805922 | -0,0412575 |
| PMM2 | 0,0536687 | -0,394273 |
| PMVK | 0,000787212 | 2,61775 |
| PNCB | 0,542044 | -0,0695079 |
| PNKP | 0,0590094 | -0,568983 |
| PNPH | 0,394412 | 0,152808 |
| PNPO | 0,0162524 | 0,495691 |
| PNPT1 | 0,568401 | -0,0802088 |
| PO210 | 0,330531 | -0,795588 |
| PON1 | 0,0153429 | 0,37033 |
| PON2 | 0,129766 | 0,892901 |
| PON3 | 0,241022 | 0,142744 |
| PP1A | 0,112487 | 0,146177 |
| PP1B | 0,574162 | -0,130777 |
| PP1G | 0,478059 | 0,270353 |
| PP1R7 | 0,218096 | 0,155593 |
| PP1R8 | 0,571868 | -0,324519 |
| PP1RA | 0,299903 | -0,60262 |
| PP1RB | 0,597519 | -0,129068 |
| PP2AA | 0,299071 | 0,128542 |
| PP2AB | 0,468793 | 0,423908 |
| PP2BA | 0,0205787 | -0,62739 |
| PP4C | 0,0638554 | 0,875594 |
| PP4R1 | 0,195335 | 0,557973 |
| PP4R2 | 0,0406202 | -1,06852 |
| PP6R1 | 0,0110774 | -0,6777 |
| PP6R3 | 0,971785 | 0,00711219 |
| PPA5 | 0,00935677 | -1,53289 |
| PPA6 | 0,0473957 | -0,405541 |
| PPAC | 0,309306 | 0,273154 |
| PPAL | 0,903374 | 0,0759331 |
| PPCE | 0,00224344 | 0,234632 |
| PPCS | 0,705719 | 0,044467 |
| PPCT | 0,00156291 | 0,898449 |
| PPGB | 0,173762 | 0,278695 |
| PPIA | 0,710253 | 0,0555563 |
| PPIB | 0,00316605 | -0,523628 |
| PPIC | 0,504829 | 0,182712 |
| PPID | 0,181782 | -0,129407 |
| PPIF | 0,0223891 | -1,42216 |
| PPIG | 0,181525 | -0,906161 |
| PPIH | 0,124984 | -0,903474 |
| PPIL1 | 0,302184 | -0,246514 |
| PPIL2 | 0,743694 | 0,185108 |
| PPIP2 | 0,309679 | 0,695756 |
| PPM1A | 0,681732 | -0,0544688 |
| PPM1B | 0,367519 | 0,147568 |
| PPM1F | 0,139101 | -0,455547 |
| PPM1G | 0,845067 | 0,0405404 |
| PPM1K | 0,824355 | 0,0513344 |
| PPME1 | 0,453262 | 0,279428 |
| PPOX | 0,0269817 | -0,70706 |
| PPP5 | 0,707217 | 0,0597474 |
| PPP6 | 0,137977 | 0,227696 |
| PPR21 | 0,199868 | 0,806889 |
| PPT1 | 0,000575021 | -0,804881 |
| PPT2 | 0,888119 | -0,103291 |
| PPWD1 | 0,295741 | -0,64723 |
| PR38A | 0,955304 | -0,0122328 |
| PR40A | 0,0790258 | -0,509546 |
| PRAF3 | 0,0512105 | 0,480013 |
| PRC2A | 0,0476413 | -0,513655 |
| PRC2C | 0,656559 | -0,130867 |
| PRDX1 | 0,351111 | 0,164776 |
| PRDX2 | 0,847634 | -0,0593837 |
| PRDX3 | 0,000625057 | 1,5128 |
| PRDX4 | 0,00108902 | -0,562158 |
| PRDX5 | 0,0655443 | -0,384239 |
| PRDX6 | 0,00024903 | 0,735726 |
| PREB | 0,276944 | 0,185821 |
| PRELP | 0,0479984 | -0,379841 |
| PREP | 0,0107726 | -0,28798 |
| PRKRA | 0,0264809 | -1,0559 |
| PROD | 6,44E-10 | 2,46772 |
| PROD2 | 9,86E-10 | -1,41897 |
| PROP | 0,719096 | -0,168328 |
| PROSC | 0,185669 | -0,157732 |
| PROX1 | 0,383088 | -0,461111 |
| PRP19 | 0,110212 | 0,698874 |
| PRP31 | 0,0113406 | -1,3316 |
| PRP39 | 0,595299 | -0,276589 |
| PRP4 | 0,0687734 | 0,895986 |
| PRP4B | 0,0642579 | 1,49547 |
| PRP6 | 0,141357 | -0,338078 |
| PRP8 | 0,844314 | 0,0293376 |
| PRPF3 | 0,512777 | 0,296137 |
| PRPK | 0,417434 | 0,202264 |
| PRPS1 | 0,378863 | -0,225133 |
| PRPS2 | 0,307615 | -0,636581 |
| PRRC1 | 0,153068 | -1,07062 |
| PRS10 | 0,0207828 | 0,294707 |
| PRS4 | 0,0165825 | 0,350973 |
| PRS6A | 0,00302345 | 0,353648 |
| PRS6B | 0,00126264 | 0,382202 |
| PRS7 | 0,000120501 | 0,516329 |
| PRS8 | 7,62E-05 | 0,498637 |
| PRUNE | 0,0212343 | 0,579035 |
| PRXD1 | 0,114764 | -0,433442 |
| PSA | 0,917826 | -0,0135326 |
| PSA1 | 0,00209918 | 0,392565 |
| PSA2 | 0,46985 | 0,48724 |
| PSA3 | 0,0446374 | 0,330629 |
| PSA4 | 0,0718715 | 0,363545 |
| PSA5 | 0,00177054 | 0,468335 |
| PSA6 | 0,124367 | 0,280475 |
| PSA7 | 0,0248538 | 0,350109 |
| PSB1 | 1,83E-05 | 0,59133 |
| PSB10 | 0,307904 | 0,3517 |
| PSB2 | 0,0349526 | 0,299109 |
| PSB3 | 0,222722 | 0,280963 |
| PSB4 | 0,977963 | -0,00570202 |
| PSB5 | 0,0878395 | 0,544665 |
| PSB6 | 0,54244 | 0,108062 |
| PSB7 | 0,19143 | 1,51046 |
| PSB8 | 0,3085 | 0,229739 |
| PSB9 | 0,466166 | -0,968072 |
| PSD11 | 0,062322 | 0,336196 |
| PSD12 | 0,00150958 | 0,486458 |
| PSD13 | 0,0257687 | 0,306332 |
| PSDE | 0,00348176 | 0,485772 |
| PSF3 | 0,263203 | -1,69229 |
| PSIP1 | 0,260215 | -0,512653 |
| PSMD1 | 0,000541143 | 0,520019 |
| PSMD2 | 0,0101859 | 0,43525 |
| PSMD3 | 0,000117233 | 0,524546 |
| PSMD4 | 0,0742639 | 0,419827 |
| PSMD5 | 0,222215 | 0,276182 |
| PSMD6 | 0,000746302 | 0,458678 |
| PSMD7 | 0,142773 | 0,323274 |
| PSMD8 | 0,0122403 | 0,359927 |
| PSMD9 | 0,974622 | -0,00536791 |
| PSME1 | 0,40184 | 0,121168 |
| PSME2 | 0,756567 | 0,0498091 |
| PSME3 | 0,244585 | 0,160032 |
| PSME4 | 0,173572 | 0,695364 |
| PSMF1 | 0,0100072 | 0,418051 |
| PSMG1 | 0,81435 | 0,0443643 |
| PSMG2 | 0,313898 | 0,398898 |
| PSMG3 | 0,256882 | 0,694467 |
| PSN2 | 0,384631 | -0,258868 |
| PSPC1 | 0,720164 | 0,129959 |
| PTBP1 | 0,206491 | -0,261078 |
| PTBP3 | 0,326518 | 0,226884 |
| PTCD1 | 0,680891 | -0,123926 |
| PTCD3 | 0,139361 | -0,259377 |
| PTER | 0,0359812 | -0,395317 |
| PTGR1 | 0,00384218 | -0,91896 |
| PTGR2 | 0,0947667 | -0,486659 |
| PTH2 | 0,14003 | -1,3146 |
| PTMA | 0,307854 | 2,12247 |
| PTN1 | 0,552541 | 0,15955 |
| PTN11 | 0,5837 | 0,0740913 |
| PTN12 | 0,812716 | 0,100037 |
| PTN23 | 0,637779 | 0,207812 |
| PTN5 | 0,389128 | 0,709968 |
| PTN6 | 0,855811 | 0,0277348 |
| PTN9 | 0,0273723 | 0,71865 |
| PTPA | 0,331888 | 0,212581 |
| PTPM1 | 0,183099 | 0,477276 |
| PTPRC | 0,177743 | -0,759401 |
| PTPRD | 0,0424441 | -0,760559 |
| PTPRF | 0,370492 | -0,459818 |
| PTPRJ | 0,193871 | -0,634511 |
| PTPRK | 0,0122078 | 0,983706 |
| PTRF | 0,0281867 | -1,63107 |
| PUF60 | 0,869127 | 0,0129887 |
| PUM1 | 0,244038 | 1,01421 |
| PUM2 | 0,402274 | -0,48722 |
| PUR2 | 0,0665338 | -0,270384 |
| PUR4 | 0,240322 | -0,367329 |
| PUR6 | 0,0164542 | 0,293112 |
| PUR8 | 0,00122995 | 0,448184 |
| PUR9 | 0,641093 | -0,0523074 |
| PURA | 0,555191 | -0,13336 |
| PURA1 | 0,7677 | -0,112745 |
| PURA2 | 0,00300106 | 1,00567 |
| PURB | 0,616807 | -0,327607 |
| PUSL1 | 0,114219 | -0,959992 |
| PVRL2 | 0,952352 | 0,0330327 |
| PX11A | 0,930725 | -0,0505431 |
| PX11B | 0,113012 | 0,383914 |
| PX11C | 0,00135949 | 1,3028 |
| PXMP2 | 0,994768 | -0,00199572 |
| PXMP4 | 0,132711 | 0,822857 |
| PYC | 8,40E-05 | -0,417076 |
| PYGB | 0,255386 | -0,154753 |
| PYGL | 7,39E-05 | -0,6634 |
| PYR1 | 0,881318 | 0,030708 |
| PYRD | 0,00775523 | -0,580996 |
| PYRG1 | 0,221314 | 0,780843 |
| PYRG2 | 0,0413717 | 0,488124 |
| QCR1 | 1,69E-06 | -0,482435 |
| QCR2 | 0,00162391 | -0,575072 |
| QCR6 | 0,662247 | -0,388339 |
| QCR7 | 0,000530518 | -0,838333 |
| QCR8 | 0,183111 | -0,576462 |
| QCR9 | 0,367333 | -0,176391 |
| QIL1 | 0,376262 | 0,251324 |
| QKI | 0,29414 | 0,429332 |
| QOR | 0,520689 | 0,110572 |
| QORL1 | 0,663687 | 0,122117 |
| QORL2 | 0,0042033 | -1,03993 |
| QSOX1 | 0,35193 | 0,506569 |
| RAB10 | 0,383708 | 0,455926 |
| RAB12 | 0,711908 | -0,167907 |
| RAB13 | 0,189988 | -0,528916 |
| RAB14 | 0,0309292 | -0,184758 |
| RAB17 | 0,0935838 | -1,02038 |
| RAB18 | 0,0306515 | 0,179194 |
| RAB1A | 0,544598 | -0,345633 |
| RAB1B | 0,258381 | -0,0841198 |
| RAB21 | 0,774744 | 0,0617085 |
| RAB2A | 0,341259 | -0,0808283 |
| RAB31 | 0,215109 | -0,559838 |
| RAB32 | 0,0136362 | -0,512042 |
| RAB35 | 0,961036 | 0,0341756 |
| RAB43 | 0,51346 | -0,16523 |
| RAB4A | 0,320735 | -0,512194 |
| RAB4B | 0,50312 | -0,564276 |
| RAB5A | 0,161785 | 0,524001 |
| RAB5B | 0,29801 | 0,490829 |
| RAB5C | 0,32534 | -0,174218 |
| RAB6A | 0,0295408 | 0,303409 |
| RAB7A | 0,163273 | -0,205 |
| RAB8A | 0,998254 | 0,00072066 |
| RAB8B | 0,362824 | -0,227927 |
| RAB9A | 0,318002 | -0,266403 |
| RABE1 | 0,974302 | 0,0274334 |
| RABE2 | 0,0321818 | 2,36285 |
| RABL3 | 0,577753 | -0,279714 |
| RABX5 | 0,286051 | 0,569462 |
| RAC1 | 0,881563 | -0,0311718 |
| RAC2 | 0,679172 | -0,170191 |
| RAD21 | 0,364421 | 0,55871 |
| RAD50 | 0,914779 | -0,0305363 |
| RADI | 0,0592013 | -0,164762 |
| RAE1L | 0,27087 | -0,256065 |
| RAF1 | 0,878364 | 0,0473868 |
| RAGP1 | 0,1928 | 0,341595 |
| RAI14 | 0,286595 | -0,306789 |
| RAIN | 0,0627563 | -0,968422 |
| RALA | 0,849098 | 0,0336669 |
| RALB | 0,411777 | -0,217933 |
| RALY | 0,337558 | 0,268969 |
| RAN | 0,547358 | 0,0974808 |
| RANB3 | 0,546644 | -0,103594 |
| RANG | 0,763902 | 0,0773799 |
| RAP1A | 0,253254 | 0,319716 |
| RAP1B | 0,244371 | -0,175988 |
| RAP2C | 0,0109654 | 0,590799 |
| RARR2 | 0,0594857 | 0,429589 |
| RASH | 0,692354 | 0,218786 |
| RASK | 0,640761 | -0,133997 |
| RASN | 0,94954 | 0,0217797 |
| RB11B | 0,318472 | -0,110598 |
| RB3GP | 0,0267949 | 0,58334 |
| RB6I2 | 0,680996 | 0,0942494 |
| RBBP4 | 0,723738 | 0,0714022 |
| RBBP6 | 0,371486 | -0,239492 |
| RBBP7 | 0,268156 | -0,199054 |
| RBBP9 | 0,44946 | 0,220712 |
| RBGP1 | 0,978405 | -0,0203797 |
| RBGPR | 0,189979 | 0,382774 |
| RBM10 | 0,274589 | -0,222714 |
| RBM12 | 0,296198 | -0,564913 |
| RBM14 | 0,925728 | 0,0207135 |
| RBM22 | 0,0734439 | 0,525402 |
| RBM25 | 0,809981 | -0,0444485 |
| RBM26 | 0,601169 | 0,118784 |
| RBM3 | 0,266113 | 1,04726 |
| RBM39 | 0,135484 | -0,237466 |
| RBM4 | 0,294715 | -0,243025 |
| RBM46 | 0,704977 | 0,472389 |
| RBM47 | 0,0204475 | 1,00857 |
| RBM8A | 0,324194 | -0,706283 |
| RBMS1 | 0,285914 | 0,719759 |
| RBMX | 0,676863 | 0,0975622 |
| RBP10 | 0,327268 | 0,401652 |
| RBP2 | 0,158846 | -0,157761 |
| RBPMS | 0,756358 | -0,0877682 |
| RBPS2 | 0,0212797 | -1,48094 |
| RBSK | 0,503597 | -0,429518 |
| RBX1 | 0,641975 | -0,142756 |
| RCC1 | 0,801537 | 0,0357389 |
| RCC2 | 0,123373 | 0,29095 |
| RCD1 | 0,185935 | -0,851784 |
| RCN1 | 0,787649 | -0,148977 |
| RD23A | 0,417454 | 0,648405 |
| RD23B | 0,449531 | 0,133113 |
| RDH1 | 0,0264103 | -2,46149 |
| RDH10 | 0,274698 | -0,242259 |
| RDH11 | 2,99E-05 | 1,95114 |
| RDH14 | 0,861411 | -0,0453774 |
| RDH7 | 0,36071 | 0,121675 |
| RED | 0,265307 | -0,283838 |
| REEP3 | 0,883075 | 0,089454 |
| REEP6 | 0,148318 | 0,28741 |
| RENT1 | 0,681264 | 0,0499942 |
| REQU | 0,101955 | 0,451137 |
| RER1 | 0,45976 | 0,158358 |
| RET1 | 0,000110797 | -0,775126 |
| RET4 | 0,038453 | 2,15063 |
| RETST | 0,0706336 | -1,07441 |
| RFA1 | 0,584729 | 0,0770556 |
| RFA2 | 0,0403894 | 0,923643 |
| RFA3 | 0,559291 | -0,51583 |
| RFC2 | 0,557298 | 0,454781 |
| RFC3 | 0,352799 | 0,36853 |
| RFC4 | 0,689062 | -0,124782 |
| RGL3 | 0,0944799 | -0,517158 |
| RGN | 0,00390565 | 0,463195 |
| RHEB | 0,521015 | 0,504844 |
| RHG01 | 0,957178 | -0,00810083 |
| RHG05 | 0,264712 | 0,39936 |
| RHG17 | 0,111386 | 0,919806 |
| RHOA | 0,539903 | 0,0803951 |
| RHOB | 0,0156133 | 1,44207 |
| RHOC | 0,55865 | 0,555277 |
| RHOD | 0,128485 | -0,794016 |
| RHOG | 0,146972 | -0,387808 |
| RIFK | 0,973738 | 0,01979 |
| RIN3 | 0,456729 | -0,415768 |
| RING2 | 0,798077 | -0,112984 |
| RINI | 0,942119 | 0,0146999 |
| RIPK1 | 0,212722 | 0,255047 |
| RIR1 | 0,224295 | 0,674698 |
| RISC | 0,671376 | 0,20057 |
| RL10 | 0,986672 | -0,00573858 |
| RL10A | 0,134814 | -0,155368 |
| RL11 | 0,202567 | -0,268284 |
| RL12 | 0,147606 | -0,18667 |
| RL13 | 0,372094 | -0,21947 |
| RL13A | 0,0890569 | -0,631296 |
| RL14 | 0,0308705 | -0,719399 |
| RL15 | 0,0101878 | -1,42362 |
| RL17 | 0,862703 | -0,0570008 |
| RL18 | 0,00227455 | -2,39012 |
| RL18A | 0,160592 | -0,512259 |
| RL21 | 0,754013 | -0,109971 |
| RL22 | 0,0637191 | -0,380367 |
| RL22L | 0,864188 | 0,0966574 |
| RL23 | 0,0140371 | -0,458254 |
| RL23A | 0,716513 | 0,0731233 |
| RL24 | 0,029166 | -0,898672 |
| RL26 | 0,378383 | -0,834094 |
| RL27 | 0,639064 | -0,107677 |
| RL27A | 0,160609 | -0,406416 |
| RL3 | 0,838124 | -0,0766452 |
| RL30 | 0,120639 | -0,309869 |
| RL31 | 0,0143521 | 2,78988 |
| RL32 | 0,42823 | 0,335823 |
| RL34 | 0,171611 | -0,831096 |
| RL35A | 0,624656 | -0,17435 |
| RL36 | 0,589053 | -0,69013 |
| RL37A | 0,895167 | 0,0908292 |
| RL38 | 0,997106 | 0,000581106 |
| RL4 | 0,0162653 | -0,643878 |
| RL5 | 0,789553 | -0,0427078 |
| RL6 | 0,0911412 | -0,887988 |
| RL7 | 0,0339039 | -1,25701 |
| RL7A | 0,0483127 | -0,739054 |
| RL8 | 0,419264 | -0,269282 |
| RL9 | 0,465272 | -0,158998 |
| RLA0 | 0,342882 | -0,090917 |
| RLA1 | 0,990622 | 0,0138639 |
| RLA2 | 0,743593 | -0,087278 |
| RM01 | 0,239347 | -0,388777 |
| RM02 | 0,17872 | -1,1618 |
| RM03 | 0,257133 | -1,2029 |
| RM04 | 0,455712 | -0,132627 |
| RM09 | 0,273228 | -0,289602 |
| RM11 | 0,450055 | -0,301462 |
| RM12 | 0,0799827 | -0,546434 |
| RM13 | 0,32366 | -0,404378 |
| RM14 | 0,146981 | -0,71128 |
| RM15 | 0,936752 | -0,0195147 |
| RM16 | 0,333065 | -0,649594 |
| RM17 | 0,0985777 | -0,611293 |
| RM18 | 0,0623459 | -1,73027 |
| RM19 | 0,292235 | -0,28457 |
| RM21 | 0,746903 | -0,156305 |
| RM22 | 0,814677 | -0,0741348 |
| RM23 | 0,901981 | -0,100467 |
| RM24 | 0,0322994 | -0,81016 |
| RM27 | 0,282629 | -0,618397 |
| RM28 | 0,385018 | -0,294279 |
| RM32 | 0,198906 | -0,828132 |
| RM37 | 0,25212 | -0,170918 |
| RM38 | 0,367293 | -0,906982 |
| RM39 | 0,506811 | 0,136213 |
| RM40 | 0,289454 | 0,196039 |
| RM41 | 0,826944 | -0,212777 |
| RM44 | 0,219878 | -0,380905 |
| RM45 | 0,134951 | 0,209411 |
| RM46 | 0,332388 | 0,421476 |
| RM47 | 0,578115 | -0,161027 |
| RM48 | 0,477295 | 0,495052 |
| RM49 | 0,593694 | 0,11151 |
| RM50 | 0,974312 | 0,0192588 |
| RM53 | 0,970262 | 0,0246601 |
| RM55 | 0,276438 | -0,610472 |
| RMD1 | 9,70E-05 | -0,788187 |
| RMD2 | 0,00523694 | 0,954004 |
| RMD3 | 0,0827829 | -0,37709 |
| RMD5A | 0,129307 | 1,26115 |
| RMND1 | 0,0393015 | -0,593448 |
| RMXL1 | 0,0196426 | -0,405197 |
| RN114 | 0,194608 | 0,248755 |
| RN181 | 0,642189 | -0,385281 |
| RN213 | 0,000273323 | 1,22017 |
| RNAS4 | 9,20E-05 | 1,32759 |
| RNF14 | 0,0253264 | 0,713106 |
| RNPS1 | 0,255968 | -0,169102 |
| RNZ2 | 0,85084 | -0,038317 |
| ROA0 | 0,416172 | 1,03926 |
| ROA1 | 0,00219943 | 0,359251 |
| ROA2 | 0,355833 | 0,0886574 |
| ROA3 | 0,469046 | -0,0683215 |
| ROA3 | 0,736094 | -0,0709842 |
| ROAA | 0,226488 | 0,202696 |
| ROBO1 | 0,476554 | 0,409178 |
| ROCK1 | 0,293593 | 0,731907 |
| ROCK2 | 0,0304054 | 1,03087 |
| RPAB3 | 0,274258 | -0,621322 |
| RPAC1 | 0,466857 | 0,171076 |
| RPAP3 | 0,276709 | 0,508211 |
| RPB1 | 0,956148 | 0,0173848 |
| RPB2 | 0,107649 | 0,365441 |
| RPGF4 | 1,34E-05 | 2,87378 |
| RPN1 | 0,0176594 | -0,278173 |
| RPN2 | 0,0181455 | -0,246567 |
| RPP30 | 0,280013 | -0,91341 |
| RPR1B | 0,00210855 | -0,584354 |
| RPTOR | 0,00667031 | 2,77507 |
| RRAGA | 0,944262 | -0,031696 |
| RRAGC | 0,962963 | 0,011632 |
| RRAS | 0,0147619 | 0,565103 |
| RRAS2 | 0,516497 | -0,1575 |
| RRBP1 | 0,0103996 | -0,364205 |
| RRF2M | 0,29567 | -0,238709 |
| RRFM | 0,566714 | -0,181568 |
| RRP44 | 0,324291 | -0,786937 |
| RRP5 | 0,24568 | 0,441257 |
| RS10 | 0,138457 | -0,235844 |
| RS11 | 0,852218 | -0,0291376 |
| RS12 | 0,98633 | 0,00384108 |
| RS13 | 0,377653 | -0,202779 |
| RS14 | 0,70338 | 0,0566492 |
| RS15 | 0,601256 | 0,68073 |
| RS15A | 0,283168 | -0,228365 |
| RS16 | 0,789058 | -0,0414025 |
| RS17 | 0,0608319 | -0,361893 |
| RS18 | 0,0823391 | -0,282238 |
| RS19 | 0,634467 | 0,0913181 |
| RS2 | 0,0701803 | 0,199817 |
| RS20 | 0,28166 | -0,147927 |
| RS21 | 0,407276 | -0,193101 |
| RS24 | 0,113689 | 0,319409 |
| RS25 | 0,744593 | -0,0760978 |
| RS26 | 0,347871 | -0,770102 |
| RS27 | 0,635458 | -0,318709 |
| RS27A | 0,562442 | -0,0730413 |
| RS27L | 0,583553 | -0,299248 |
| RS28 | 0,721028 | -0,131453 |
| RS3 | 0,0424999 | -0,246859 |
| RS30 | 0,301407 | -0,868628 |
| RS3A | 0,714594 | -0,0420252 |
| RS4X | 0,902242 | -0,0303548 |
| RS5 | 0,763715 | 0,268111 |
| RS6 | 0,346502 | -0,262473 |
| RS7 | 0,969704 | -0,0265226 |
| RS8 | 0,0493079 | -0,397004 |
| RS9 | 0,179905 | -0,477825 |
| RSMB | 0,831825 | 0,050471 |
| RSSA | 0,0979014 | -0,198645 |
| RSU1 | 0,4884 | -0,254297 |
| RT05 | 0,794634 | -0,0892827 |
| RT06 | 0,531878 | -0,462565 |
| RT07 | 0,994585 | 0,000638326 |
| RT09 | 0,129863 | -0,381986 |
| RT10 | 0,091802 | 0,427441 |
| RT11 | 0,255572 | -0,34816 |
| RT14 | 0,384689 | -0,599126 |
| RT15 | 0,390568 | -0,216328 |
| RT16 | 0,90838 | -0,0348291 |
| RT17 | 0,512339 | -0,142147 |
| RT18A | 0,764011 | 0,0676877 |
| RT21 | 0,512664 | 0,541838 |
| RT22 | 0,348755 | 0,215498 |
| RT23 | 0,154891 | -0,350379 |
| RT24 | 0,176743 | 1,01815 |
| RT25 | 0,202877 | -0,555698 |
| RT26 | 0,680455 | 0,0933965 |
| RT27 | 0,8022 | -0,0833054 |
| RT28 | 0,591297 | 0,398645 |
| RT29 | 0,731298 | 0,0561352 |
| RT30 | 0,519753 | 0,19384 |
| RT31 | 0,851091 | 0,0432173 |
| RT34 | 0,532783 | 0,446879 |
| RT35 | 0,353187 | -0,26004 |
| RT36 | 0,037577 | -0,839132 |
| RT4I1 | 0,00535903 | -0,542293 |
| RTCB | 0,0374874 | 0,381511 |
| RTN4 | 0,0160509 | 0,987548 |
| RTN4 | 0,126527 | 0,865563 |
| RU17 | 0,792136 | -0,0368935 |
| RU1C | 0,435125 | 0,325849 |
| RU2A | 0,464838 | -0,149408 |
| RU2B | 0,191182 | -0,481413 |
| RUFY1 | 0,299874 | -0,387441 |
| RUFY2 | 0,0869949 | -1,0683 |
| RUFY3 | 0,51599 | 0,187817 |
| RUSD3 | 0,554799 | -0,307917 |
| RUVB1 | 0,801117 | -0,0412518 |
| RUVB2 | 0,876259 | -0,0206356 |
| RUXE | 0,483972 | 0,643765 |
| RUXF | 0,992775 | -0,00775973 |
| RUXG | 0,408578 | -0,48343 |
| RWDD1 | 0,446778 | -0,541279 |
| RWDD4 | 0,268134 | 0,48192 |
| RXRA | 0,924077 | -0,0688438 |
| S10A9 | 0,915307 | 0,039615 |
| S10AA | 0,696117 | -0,317753 |
| S10AB | 0,304966 | -0,8052 |
| S10AD | 0,913497 | 0,0860475 |
| S12A4 | 0,00607915 | 2,77676 |
| S12A7 | 0,809096 | 0,217816 |
| S14L2 | 0,0147435 | 0,370822 |
| S14L4 | 0,10534 | -0,107305 |
| S22A1 | 3,29E-05 | 3,261 |
| S22A3 | 2,57E-05 | 3,09652 |
| S22AI | 0,147639 | 0,90353 |
| S23IP | 0,0116906 | 0,412103 |
| S2542 | 0,117715 | -0,462333 |
| S2545 | 0,052276 | 1,6226 |
| S2546 | 0,258617 | -0,608323 |
| S26A1 | 0,0182208 | 0,578881 |
| S27A2 | 0,0931884 | -0,216334 |
| S27A4 | 0,434904 | 0,612502 |
| S27A5 | 0,075273 | -0,202195 |
| S29A1 | 0,832581 | -0,1364 |
| S35B2 | 0,0640845 | 0,768603 |
| S38A3 | 0,00345334 | 1,20848 |
| S38A4 | 0,004259 | 1,60202 |
| S38AA | 0,844109 | 0,097161 |
| S40A1 | 0,0629944 | 1,16714 |
| S4A4 | 4,75E-05 | 1,27423 |
| S61A1 | 0,879778 | -0,173728 |
| S6A12 | 0,00518778 | -0,525918 |
| S6A13 | 6,57E-05 | -0,682438 |
| SAC1 | 0,0304103 | 0,447972 |
| SAC2 | 0,137857 | 1,49333 |
| SAE1 | 0,725648 | -0,0834195 |
| SAE2 | 0,0357147 | -0,529064 |
| SAFB1 | 0,970569 | 0,0227493 |
| SAFB2 | 0,312407 | -0,155291 |
| SAHH | 0,00392445 | -0,327744 |
| SAHH2 | 0,140623 | 0,229102 |
| SAM50 | 0,0212582 | -0,328519 |
| SAM9L | 0,000666154 | 1,29074 |
| SAMH1 | 0,0169025 | -0,282647 |
| SAMP | 0,000185242 | -2,5096 |
| SAP | 0,121955 | -0,304579 |
| SAP3 | 0,00368538 | -1,81491 |
| SAR1A | 0,452617 | 0,776928 |
| SAR1B | 0,759555 | -0,115131 |
| SARAF | 0,0945888 | 0,868569 |
| SARDH | 6,74E-07 | -0,973858 |
| SARNP | 0,0880645 | -0,340988 |
| SART3 | 0,217283 | -0,358678 |
| SATT | 0,00181901 | 1,17824 |
| SBDS | 0,0991658 | -0,421447 |
| SBP1 | 1,17E-06 | -1,00919 |
| SBP2 | 3,76E-06 | -5,7761 |
| SC11A | 0,0057926 | -0,452435 |
| SC16B | 0,695517 | 0,0925989 |
| SC22B | 0,701177 | 0,0698045 |
| SC23A | 0,247239 | 0,0896053 |
| SC23B | 0,0895882 | 0,21851 |
| SC24A | 0,854972 | -0,0193787 |
| SC31A | 0,472645 | -0,09224 |
| SC61G | 0,254117 | -1,35068 |
| SCAM1 | 0,537833 | 0,163548 |
| SCAM3 | 0,0167346 | 1,46906 |
| SCFD1 | 0,0298796 | 0,294808 |
| SCFD2 | 0,129173 | 0,493799 |
| SCLY | 0,367377 | -0,178258 |
| SCMC2 | 0,227826 | 0,587494 |
| SCMC3 | 0,00678185 | -2,57105 |
| SCO1 | 0,0107013 | -2,19186 |
| SCO2 | 0,026027 | 0,742426 |
| SCRB1 | 0,00339758 | 0,832044 |
| SCRB2 | 0,209829 | -0,809376 |
| SCRIB | 0,558919 | 0,491758 |
| SCRN2 | 0,0117112 | -0,649028 |
| SCRN3 | 0,779857 | -0,0937815 |
| SCYL2 | 0,209474 | 0,376821 |
| SDF2L | 0,580047 | -0,636574 |
| SDHA | 0,0103416 | -0,347287 |
| SDHB | 0,0219906 | -0,569507 |
| SDHL | 6,21E-09 | -8,4759 |
| SDPR | 0,960536 | -0,0641677 |
| SDSL | 1,95E-05 | -2,8208 |
| SE1L1 | 0,181792 | -0,271658 |
| SEC13 | 0,194902 | -0,269986 |
| SEC20 | 0,498908 | 0,147987 |
| SEC63 | 0,836876 | -0,0307693 |
| SEH1 | 0,430014 | 0,463537 |
| SELB | 0,0498216 | 0,259697 |
| SELO | 0,0372841 | -0,770974 |
| SELS | 0,318989 | -0,315678 |
| SELT | 0,981366 | -0,0119756 |
| SENP7 | 0,0742406 | -1,42221 |
| SEPP1 | 0,0139935 | -0,889224 |
| SEPT10 | 0,146915 | -1,00714 |
| SEPT11 | 0,82214 | -0,0446494 |
| SEPT15 | 0,28171 | -0,946139 |
| SEPT2 | 0,535628 | -0,0714916 |
| SEPT4 | 0,213531 | -0,79618 |
| SEPT7 | 0,318377 | 0,213957 |
| SEPT8 | 0,901922 | 0,0500752 |
| SEPT9 | 0,290333 | -0,219993 |
| SERC | 0,054051 | 1,0754 |
| SERHL | 0,0274032 | -0,443787 |
| SERPH | 0,11067 | -0,161528 |
| SET | 0,616736 | 0,575333 |
| SETD3 | 0,00332945 | 0,526669 |
| SF01 | 0,834804 | -0,0590655 |
| SF3A1 | 0,23431 | -0,118521 |
| SF3A2 | 0,899768 | 0,105146 |
| SF3A3 | 0,44472 | -0,151157 |
| SF3B1 | 0,557528 | -0,0526352 |
| SF3B3 | 0,797878 | -0,0380847 |
| SF3B5 | 0,793406 | -0,0525455 |
| SFPQ | 0,319436 | 0,231963 |
| SFXN1 | 2,11E-08 | -3,4912 |
| SFXN2 | 0,000310123 | -0,899011 |
| SGK2 | 4,65E-07 | 2,83162 |
| SGPL1 | 0,57614 | -0,0926301 |
| SGTA | 0,351683 | -0,188023 |
| SH3G1 | 0,769288 | 0,0580638 |
| SH3L1 | 0,467928 | -0,0903692 |
| SH3L3 | 0,146715 | -1,10828 |
| SHAN3 | 0,147169 | 1,09294 |
| SHC1 | 0,000948017 | 0,698878 |
| SHLB1 | 0,0440827 | -1,4685 |
| SHLB2 | 0,353758 | -0,604118 |
| SHOC2 | 0,035811 | -1,75319 |
| SHOT1 | 0,114003 | -0,390278 |
| SHPK | 0,460949 | -0,125667 |
| SHRM1 | 0,830765 | 0,228982 |
| SHRM2 | 0,34556 | 0,303739 |
| SIA4A | 0,345886 | -0,495961 |
| SIA4C | 0,355634 | -0,545053 |
| SIAE | 0,272263 | 0,23633 |
| SIAT9 | 0,0939678 | -0,941188 |
| SIGIR | 0,0184447 | -1,27778 |
| SIK3 | 0,866696 | 0,0961472 |
| SIL1 | 0,788631 | -0,192049 |
| SIN3A | 0,818432 | 0,148332 |
| SIR2 | 0,0274921 | 0,306321 |
| SIR3 | 0,0801233 | -0,579595 |
| SIR4 | 0,38631 | 0,632545 |
| SIR5 | 0,137576 | -0,339549 |
| SK2L2 | 0,59881 | -0,0831324 |
| SKP1 | 0,974331 | -0,0061938 |
| SLIRP | 0,0549919 | -1,07443 |
| SLK | 0,592843 | 0,285976 |
| SLMAP | 0,282221 | -0,289449 |
| SLMAP | 0,123213 | -0,724556 |
| SLTM | 0,0801696 | -0,543684 |
| SMAD2 | 0,503256 | 0,206769 |
| SMAP | 0,983818 | 0,0165882 |
| SMAP1 | 0,592924 | 0,093269 |
| SMAP2 | 0,00158629 | -0,903231 |
| SMC1A | 0,095264 | -0,279575 |
| SMC2 | 0,0620677 | 1,74233 |
| SMC3 | 0,459222 | -0,309381 |
| SMCA2 | 0,793394 | 0,148428 |
| SMCA5 | 0,0254804 | 0,489995 |
| SMD1 | 0,314135 | 0,446328 |
| SMD2 | 0,818302 | 0,0551456 |
| SMD3 | 0,738751 | 0,0866782 |
| SMDC1 | 0,132598 | -0,433114 |
| SMHD1 | 0,324241 | 0,443419 |
| SMRC2 | 0,149834 | -0,194031 |
| SMRD2 | 0,8231 | 0,196075 |
| SMU1 | 0,466602 | -0,177429 |
| SMYD5 | 0,476874 | -0,21554 |
| SN | 0,0913004 | 0,926352 |
| SNAA | 0,00150396 | 0,453567 |
| SNAG | 0,0140901 | 3,83051 |
| SND1 | 0,103644 | -0,163821 |
| SNF8 | 0,146095 | -0,437224 |
| SNP23 | 0,83979 | 0,0542612 |
| SNP29 | 0,495703 | -0,237936 |
| SNP47 | 0,498112 | -0,295878 |
| SNR40 | 0,859847 | 0,155674 |
| SNRPA | 0,000573016 | -1,74499 |
| SNTA1 | 0,688505 | 0,164451 |
| SNTB1 | 0,00256668 | 0,856964 |
| SNTB2 | 0,181327 | -0,428564 |
| SNUT1 | 0,517565 | -0,206616 |
| SNUT2 | 0,286541 | -0,619346 |
| SNW1 | 0,44024 | -0,147897 |
| SNX1 | 0,396412 | 0,335335 |
| SNX12 | 0,0209848 | -0,630529 |
| SNX2 | 0,899937 | -0,012441 |
| SNX27 | 0,162311 | 1,10558 |
| SNX3 | 0,0613436 | -0,431308 |
| SNX4 | 0,732537 | 0,0908461 |
| SNX5 | 0,800014 | -0,0421063 |
| SNX6 | 0,41457 | -0,135633 |
| SNX9 | 0,696603 | -0,0599082 |
| SO1A1 | 0,0584808 | 1,78466 |
| SO1A4 | 0,00925499 | 1,29414 |
| SO1B2 | 0,806648 | 0,189715 |
| SOAT2 | 0,000772969 | -0,84074 |
| SODC | 0,0761033 | 0,363916 |
| SODE | 0,000120974 | -1,05937 |
| SODM | 0,028564 | -0,61513 |
| SON | 0,415384 | -0,1397 |
| SORCN | 0,276144 | -0,233044 |
| SOX | 0,927816 | -0,00755914 |
| SP16H | 0,700662 | -0,0905902 |
| SPA3K | 0,00628927 | -1,86393 |
| SPA3M | 0,190022 | 0,187671 |
| SPA3N | 0,14491 | -0,655747 |
| SPB6 | 0,940831 | -0,0110006 |
| SPCS | 0,118307 | -0,277045 |
| SPCS1 | 0,0750877 | -1,83749 |
| SPCS2 | 0,572258 | -0,338967 |
| SPEB | 0,629859 | 0,214266 |
| SPEE | 0,0177214 | 0,377286 |
| SPF27 | 0,327946 | -0,718579 |
| SPF30 | 0,630118 | 0,225446 |
| SPF45 | 0,26141 | 0,297893 |
| SPG20 | 0,323246 | -0,347528 |
| SPG7 | 0,0141749 | -0,48314 |
| SPHK2 | 0,219331 | 0,345316 |
| SPRC | 0,267916 | -1,05546 |
| SPRE | 0,0633601 | -0,302903 |
| SPRY4 | 0,000235526 | -0,907094 |
| SPS1 | 0,0581914 | -0,489767 |
| SPS2 | 4,88E-05 | -0,801305 |
| SPT5H | 0,396541 | 0,169322 |
| SPT6H | 0,0131065 | 0,328708 |
| SPTA1 | 0,529655 | -0,230653 |
| SPTB1 | 0,0807741 | -0,499359 |
| SPTB2 | 0,0179362 | 0,204343 |
| SPTC2 | 0,811834 | -0,0765978 |
| SPTN1 | 0,646699 | 0,0376466 |
| SPYA | 5,16E-05 | -1,24268 |
| SQRD | 0,116943 | -0,324064 |
| SQSTM | 2,85E-07 | 1,65601 |
| SR140 | 0,616689 | -0,139795 |
| SRBD1 | 0,682254 | 0,136213 |
| SRBS1 | 0,85634 | 0,0744435 |
| SRBS2 | 0,00738907 | -0,995016 |
| SRC | 0,239544 | -0,503136 |
| SRC8 | 0,80795 | -0,0356846 |
| SREK1 | 0,981695 | 0,0143814 |
| SRP09 | 0,715936 | 0,136671 |
| SRP19 | 0,368289 | -0,56273 |
| SRP54 | 0,161027 | -0,167364 |
| SRP68 | 0,259039 | -0,1728 |
| SRPR | 0,0037603 | 0,482404 |
| SRPRB | 0,00988019 | 0,219836 |
| SRR | 0,352316 | 0,271924 |
| SRRM1 | 0,550064 | 0,276189 |
| SRRM2 | 0,146385 | 0,207605 |
| SRRT | 0,2243 | -0,255152 |
| SRS10 | 0,50317 | 0,141363 |
| SRSF1 | 0,535516 | 0,0769698 |
| SRSF2 | 0,965279 | -0,00716877 |
| SRSF3 | 0,964202 | 0,0256662 |
| SRSF4 | 0,955129 | -0,0185194 |
| SRSF5 | 0,217262 | 0,452534 |
| SRSF7 | 0,160771 | -0,368624 |
| SRSF9 | 0,170099 | -0,466211 |
| SRXN1 | 0,0184692 | 1,89097 |
| SSA27 | 0,960108 | -0,0387249 |
| SSBP | 0,170453 | -0,206212 |
| SSDH | 0,00104345 | -0,35963 |
| SSFA2 | 0,158697 | -0,907307 |
| SSRA | 0,788895 | -0,132149 |
| SSRD | 0,00383355 | -0,517799 |
| SSRP1 | 0,0166412 | -0,411341 |
| ST1A1 | 0,122577 | 0,40009 |
| ST1B1 | 9,42E-07 | 1,00714 |
| ST1C2 | 4,64E-05 | -1,21164 |
| ST1D1 | 0,00744351 | -0,481186 |
| ST2A1 | 0,00228815 | -0,961441 |
| ST2A2 | 0,487096 | -1,10469 |
| STA13 | 0,791565 | -0,0911954 |
| STA5A | 0,616744 | -0,274701 |
| STA5B | 0,0228452 | -0,507195 |
| STAB1 | 0,117568 | -0,787251 |
| STAB2 | 0,000179995 | -0,833611 |
| STAG2 | 0,0906201 | -0,367766 |
| STAM1 | 0,423117 | -0,466659 |
| STAR5 | 0,563876 | 0,156597 |
| STAR7 | 0,344252 | -0,72465 |
| STAT1 | 0,0134319 | 0,541384 |
| STAT2 | 0,605562 | -0,235312 |
| STAT3 | 0,00971061 | -0,445614 |
| STAU1 | 0,0586339 | 0,267203 |
| STBD1 | 0,814453 | 0,102575 |
| STEA3 | 0,308679 | 0,840952 |
| STEA4 | 0,653951 | 0,0875009 |
| STIM1 | 0,0321052 | -0,363683 |
| STIP1 | 0,665935 | -0,0554212 |
| STK19 | 0,738962 | -0,660958 |
| STK24 | 0,920151 | -0,0155408 |
| STK38 | 0,12636 | 0,417643 |
| STK39 | 0,00510953 | 1,69155 |
| STK4 | 0,0770535 | 0,813217 |
| STML2 | 0,279932 | -0,302352 |
| STMN1 | 0,192683 | 0,645336 |
| STOM | 3,34E-05 | -1,79179 |
| STRAP | 0,202955 | 0,176905 |
| STRN | 0,485665 | -0,253493 |
| STRN3 | 0,260588 | 0,426775 |
| STRN4 | 0,885262 | -0,084329 |
| STRP1 | 0,978244 | -0,0132624 |
| STRUM | 0,806829 | 0,100801 |
| STS | 0,00844135 | 1,22779 |
| STT3A | 0,0177062 | -0,440587 |
| STT3B | 0,0176034 | 0,237446 |
| STX12 | 0,821654 | 0,0621297 |
| STX18 | 0,0693443 | 1,04129 |
| STX4 | 0,0947543 | 0,553718 |
| STX5 | 0,325824 | 0,32235 |
| STX7 | 0,631676 | -0,153697 |
| STX8 | 0,315154 | -0,143871 |
| STXB1 | 0,00297684 | -1,00338 |
| STXB2 | 0,486803 | 0,619504 |
| STXB3 | 0,643934 | 0,0418173 |
| SUCA | 0,0364795 | -0,804042 |
| SUCB1 | 4,39E-06 | -0,657733 |
| SUCB2 | 6,25E-05 | -0,940124 |
| SUCHY | 0,0414365 | -1,30715 |
| SUGT1 | 0,0404837 | 0,357273 |
| SUMF1 | 0,378404 | 0,189885 |
| SUMO1 | 0,42312 | 0,797693 |
| SUN2 | 0,106074 | 0,547346 |
| SUOX | 0,164241 | -0,2705 |
| SURF1 | 0,785693 | -0,128353 |
| SURF4 | 0,454342 | 0,483558 |
| SUV3 | 0,34231 | -0,376637 |
| SVIL | 0,145696 | -0,637184 |
| SYAC | 2,19E-08 | 1,60709 |
| SYAM | 0,557058 | -0,103185 |
| SYAP1 | 0,708467 | 0,123981 |
| SYCC | 0,464235 | -0,162942 |
| SYCM | 0,509508 | -0,437733 |
| SYDC | 0,945936 | -0,00820255 |
| SYDM | 0,848645 | 0,0684595 |
| SYEM | 0,0598612 | 0,376334 |
| SYEP | 0,308136 | 0,134292 |
| SYFA | 0,729584 | -0,0272099 |
| SYFB | 0,419196 | -0,0730101 |
| SYFM | 0,00199218 | -0,621051 |
| SYG | 0,000452619 | 0,355381 |
| SYHC | 0,83961 | -0,0249748 |
| SYHM | 0,220598 | -0,358397 |
| SYIC | 0,0179024 | 0,265475 |
| SYIM | 0,692724 | -0,0520054 |
| SYJ2B | 0,103706 | -0,370526 |
| SYK | 0,528368 | 0,0804949 |
| SYLC | 0,0102478 | 0,269754 |
| SYLM | 0,15053 | -0,652943 |
| SYMC | 0,797155 | -0,0457751 |
| SYMPK | 0,787123 | 0,0507154 |
| SYNC | 0,00234962 | 0,416761 |
| SYNE1 | 0,363324 | -0,764626 |
| SYNRG | 0,872951 | -0,140958 |
| SYPM | 0,220138 | -0,500328 |
| SYRC | 0,840594 | 0,0260846 |
| SYRM | 0,137864 | 0,532689 |
| SYSC | 0,0149911 | 0,293957 |
| SYSM | 0,962906 | 0,00858243 |
| SYTC | 0,000360498 | -0,520888 |
| SYTC2 | 0,028704 | -1,16259 |
| SYTM | 0,786656 | 0,0909182 |
| SYUA | 0,0662378 | -0,917037 |
| SYVC | 0,998407 | -0,000225067 |
| SYVM | 0,657722 | 0,289135 |
| SYWC | 0,30144 | 0,094346 |
| SYWM | 0,516621 | -0,32699 |
| SYYC | 0,952877 | -0,00750415 |
| SYYM | 0,957614 | 0,0295312 |
| SZRD1 | 0,282577 | -0,563586 |
| T10B | 0,150409 | -0,487601 |
| T120A | 0,242213 | 0,669943 |
| T126A | 0,00376247 | -0,621414 |
| T126B | 0,0761734 | -1,91754 |
| T22D1 | 0,295228 | 0,616458 |
| T23O | 1,91E-06 | -1,08028 |
| T2FA | 0,918303 | 0,0724376 |
| T2FB | 0,389436 | 0,255857 |
| T3HPD | 0,0329535 | -0,549176 |
| TACO1 | 0,60308 | -0,0697228 |
| TADBP | 0,827314 | 0,0621036 |
| TAGL | 0,478655 | 0,170059 |
| TAGL2 | 0,716489 | -0,0598647 |
| TALDO | 0,283743 | -0,149683 |
| TAM41 | 0,0209398 | -1,90532 |
| TAOK3 | 0,0380737 | 0,430286 |
| TAP1 | 0,382152 | 0,492501 |
| TAP2 | 0,0200799 | 0,499197 |
| TAPT1 | 0,666298 | -0,117415 |
| TARA | 0,756543 | -0,175906 |
| TATD1 | 0,779787 | 0,251884 |
| TB182 | 0,969022 | -0,00523408 |
| TBA1C | 0,974791 | -0,00297705 |
| TBA4A | 0,543441 | -0,227632 |
| TBA8 | 0,000765924 | 2,80503 |
| TBB2A | 0,628057 | -0,0931009 |
| TBB4B | 0,640808 | -0,065719 |
| TBB5 | 0,0306901 | -0,560753 |
| TBB6 | 0,459733 | 0,178494 |
| TBC15 | 0,651056 | 0,0952826 |
| TBC17 | 0,8383 | -0,0542431 |
| TBC24 | 0,408778 | -0,563025 |
| TBC8B | 0,0199438 | -1,58351 |
| TBC9B | 0,51318 | -0,350298 |
| TBCA | 0,212669 | 1,47156 |
| TBCB | 8,54E-05 | 1,50929 |
| TBCC | 0,0970313 | 0,981028 |
| TBCD | 0,0673348 | 1,09618 |
| TBCE | 8,56E-05 | 1,15955 |
| TBCEL | 1,00E-09 | 2,12265 |
| TBG2 | 0,477168 | -0,255721 |
| TBL1R | 0,343287 | 0,228944 |
| TBL1X | 0,985489 | -0,00811036 |
| TBL2 | 0,649415 | 0,0797195 |
| TBL3 | 0,073778 | 0,370627 |
| TCEA1 | 0,0347088 | -0,239347 |
| TCOF | 0,157495 | -0,452145 |
| TCP4 | 0,172204 | 0,242181 |
| TCPA | 0,0088679 | 0,369372 |
| TCPB | 0,0024141 | 0,381792 |
| TCPD | 0,0134187 | 0,362152 |
| TCPE | 0,0172422 | 0,405332 |
| TCPG | 0,000215985 | 0,337055 |
| TCPH | 0,00478461 | 0,34458 |
| TCPQ | 0,00593644 | 0,283304 |
| TCPZ | 0,0742989 | 0,211661 |
| TCRG1 | 0,561563 | -0,11845 |
| TCTP | 0,263942 | 0,215576 |
| TEBP | 0,496625 | 0,104538 |
| TECR | 0,843475 | -0,152437 |
| TEFM | 0,233767 | 0,861988 |
| TENA | 0,00854452 | -1,18189 |
| TENC1 | 0,111774 | -0,46473 |
| TENS3 | 0,233277 | -0,809261 |
| TERA | 0,000642432 | 0,520837 |
| TETN | 0,00068829 | -2,3804 |
| TEX2 | 0,0208637 | 1,6071 |
| TF2B | 0,867435 | -0,077007 |
| TF3C4 | 0,907977 | -0,0457548 |
| TF65 | 0,378237 | 0,285314 |
| TFAM | 0,403519 | -0,273575 |
| TFP11 | 0,315196 | -0,583536 |
| TFR1 | 0,148194 | -0,350271 |
| TFR2 | 0,000772896 | -1,1923 |
| TGM1 | 0,0208613 | -0,526319 |
| TGM2 | 0,000495609 | 0,405824 |
| THAS | 0,310087 | 0,799349 |
| THEM4 | 0,0484867 | -0,694348 |
| THIC | 0,854606 | 0,0576582 |
| THIKA | 3,45E-09 | 1,65322 |
| THIKB | 5,72E-07 | 1,94708 |
| THIL | 4,80E-08 | -1,42126 |
| THIM | 0,000351084 | -0,752967 |
| THIO | 0,348041 | 0,34881 |
| THIOM | 0,927968 | 0,0415284 |
| THNS2 | 6,63E-05 | 0,71845 |
| THOC1 | 0,545692 | 0,40339 |
| THOC2 | 0,865122 | 0,0332677 |
| THOC3 | 0,880252 | -0,0917981 |
| THOC4 | 0,15575 | 0,190612 |
| THOC5 | 0,358855 | -0,595438 |
| THOC6 | 0,727156 | -0,0945594 |
| THOP1 | 0,643957 | 0,0794071 |
| THRB | 0,309591 | 0,15539 |
| THTM | 0,144937 | -0,165414 |
| THTPA | 0,152844 | -1,14214 |
| THTR | 0,57371 | 0,084252 |
| THUM1 | 0,187666 | 0,432916 |
| THUM3 | 0,0698632 | 1,21266 |
| TI17B | 0,241163 | 0,165873 |
| TIAR | 0,56545 | -0,361628 |
| TIDC1 | 0,128586 | 0,708047 |
| TIF1B | 0,457382 | -0,119419 |
| TIM10 | 0,0192588 | -1,4939 |
| TIM13 | 0,0366135 | -0,544601 |
| TIM14 | 0,0579756 | -1,39738 |
| TIM16 | 0,143013 | -0,39529 |
| TIM21 | 0,00294459 | -0,496963 |
| TIM23 | 0,587459 | -0,0546551 |
| TIM44 | 0,0206447 | -0,31484 |
| TIM50 | 0,0423197 | -0,71051 |
| TIM8A | 0,206237 | -0,604315 |
| TIM8B | 0,00775745 | -2,15551 |
| TIM9 | 0,123316 | -0,757402 |
| TINAL | 0,428637 | 0,252857 |
| TIPRL | 0,436048 | -0,371612 |
| TKT | 0,000298785 | 0,449518 |
| TLDC1 | 0,887283 | -0,100919 |
| TLE1 | 0,802476 | 0,133096 |
| TLN1 | 0,132991 | 0,116078 |
| TLN2 | 0,00503801 | -0,729948 |
| TLR13 | 0,483554 | 0,466746 |
| TM109 | 0,938741 | -0,0169764 |
| TM135 | 0,0488803 | 0,741516 |
| TM205 | 0,977735 | -0,00569375 |
| TM214 | 0,214085 | 0,187538 |
| TM260 | 0,135924 | -1,02697 |
| TM41B | 0,15446 | 0,660603 |
| TM6S2 | 0,385351 | 0,163852 |
| TM9S1 | 0,394458 | -0,453598 |
| TM9S2 | 0,659189 | -0,0748879 |
| TM9S3 | 0,416714 | 0,201575 |
| TM9S4 | 0,290251 | 0,215875 |
| TMCO1 | 0,84049 | 0,0506671 |
| TMED1 | 0,0984736 | -0,372653 |
| TMED2 | 0,240527 | -0,73817 |
| TMED4 | 0,895875 | 0,131196 |
| TMED5 | 0,00166086 | 0,985937 |
| TMED9 | 0,219871 | -0,494402 |
| TMEDA | 0,783869 | -0,0708526 |
| TMLH | 0,567763 | -0,144295 |
| TMM11 | 0,0161754 | -0,660869 |
| TMM19 | 0,499634 | -0,514414 |
| TMM33 | 0,225401 | 0,421573 |
| TMM43 | 0,112339 | -1,1 |
| TMOD3 | 0,58525 | 0,147805 |
| TMX1 | 0,147032 | 0,378074 |
| TMX2 | 0,0644406 | 1,30936 |
| TMX3 | 0,208767 | -0,368148 |
| TNG2 | 0,242752 | 0,775324 |
| TNPO1 | 0,561805 | -0,132683 |
| TNPO2 | 0,246889 | 0,713909 |
| TNPO3 | 0,664119 | -0,107275 |
| TNR6 | 0,173931 | 0,820307 |
| TOIP1 | 0,141718 | 0,300601 |
| TOIP2 | 0,0947817 | 0,318668 |
| TOLIP | 0,223246 | -0,715677 |
| TOM1 | 0,543312 | 0,440813 |
| TOM22 | 0,579623 | -0,312655 |
| TOM40 | 0,0677997 | -0,439366 |
| TOM70 | 0,00527941 | -0,401837 |
| TOP1 | 0,101748 | 0,152388 |
| TOP2A | 0,359125 | -0,478808 |
| TOP2B | 0,00958429 | -0,323133 |
| TOP3B | 0,400592 | -0,231531 |
| TOR1A | 0,694363 | 0,174918 |
| TOR1B | 0,336446 | 0,541225 |
| TP4A1 | 0,515651 | 0,302907 |
| TPC11 | 0,216082 | -0,3944 |
| TPC12 | 0,258516 | -0,540232 |
| TPD52 | 0,530554 | 0,208009 |
| TPD54 | 0,985893 | 0,00625006 |
| TPIS | 0,127749 | -0,346626 |
| TPK1 | 0,418355 | -0,192933 |
| TPM1 | 0,0269511 | 0,847375 |
| TPM3 | 0,103164 | 0,296969 |
| TPM4 | 0,258694 | -0,202191 |
| TPMT | 0,603335 | 0,140351 |
| TPP2 | 0,528633 | -0,109068 |
| TPPC1 | 0,542678 | 0,311801 |
| TPPC3 | 0,340212 | -0,410886 |
| TPPC5 | 0,106001 | 1,16379 |
| TPR | 0,238386 | -0,133537 |
| TPRKB | 0,214097 | -1,06245 |
| TPSN | 0,00565115 | 0,792221 |
| TPSNR | 0,676522 | 0,248069 |
| TR150 | 0,560412 | 0,146981 |
| TRA2A | 0,143132 | 0,248384 |
| TRA2B | 0,119443 | -0,482951 |
| TRABD | 0,0422705 | -0,889179 |
| TRADD | 0,597215 | 0,499682 |
| TRAP1 | 0,0912162 | -0,163493 |
| TRFE | 0,531357 | 0,0667604 |
| TRFL | 0,630841 | -0,219141 |
| TRI14 | 0,0272305 | 0,501367 |
| TRI25 | 0,738588 | 0,0835654 |
| TRI32 | 0,261211 | 0,631224 |
| TRI56 | 0,812623 | 0,121797 |
| TRIPC | 0,450207 | -0,473496 |
| TRM1 | 0,468163 | -0,271475 |
| TRM61 | 0,0417478 | 0,498091 |
| TRNT1 | 0,715578 | -0,0813939 |
| TRXR1 | 0,0202308 | 0,55931 |
| TRXR2 | 0,000143095 | -1,26015 |
| TS101 | 0,81487 | 0,038888 |
| TSN | 0,109637 | 0,361852 |
| TSN9 | 0,103501 | 0,360212 |
| TSNAX | 0,913157 | -0,0239757 |
| TSP1 | 0,636485 | -0,131555 |
| TSR1 | 0,6244 | 0,252227 |
| TSR2 | 0,593133 | -0,288254 |
| TSSC1 | 0,161611 | 0,759875 |
| TSSC4 | 0,0931938 | -0,71484 |
| TSTD3 | 0,00419864 | -1,229 |
| TT39B | 3,74E-06 | 1,67605 |
| TT39C | 0,0857728 | 0,231455 |
| TTC36 | 0,317154 | -0,249975 |
| TTC38 | 7,53E-06 | 0,988128 |
| TTHY | 0,022141 | 0,445864 |
| TTL12 | 0,436557 | -0,0931644 |
| TTPA | 0,165922 | 0,19677 |
| TUT4 | 0,205419 | 1,35733 |
| TUT7 | 0,0673514 | 0,550039 |
| TWF1 | 0,212175 | 0,239803 |
| TX1B3 | 0,323877 | 0,617232 |
| TXD12 | 0,456628 | -0,236823 |
| TXD15 | 0,599275 | 0,251192 |
| TXD17 | 0,870569 | -0,0329622 |
| TXLNA | 0,663773 | 0,163095 |
| TXND5 | 0,401147 | 0,107963 |
| TXND9 | 0,26921 | 0,669207 |
| TXNL1 | 0,216447 | 0,285708 |
| TYB10 | 0,180492 | 1,32702 |
| TYB4 | 0,773339 | 0,0700483 |
| TYPH | 0,00741459 | -2,14516 |
| TYSD1 | 0,307302 | 0,880053 |
| TYY1 | 0,0361196 | 0,490684 |
| U119B | 9,83E-05 | 1,89586 |
| U2AF2 | 0,243842 | 0,205117 |
| U2AF4 | 0,683767 | 0,28822 |
| U520 | 0,414231 | -0,0568412 |
| U5S1 | 0,554482 | 0,0697533 |
| UAP1 | 0,00176483 | 0,557238 |
| UAP1L | 0,558684 | 0,0879828 |
| UB2J1 | 0,0251903 | 0,587687 |
| UB2L3 | 0,616035 | 0,392617 |
| UB2R1 | 0,10952 | -0,553833 |
| UB2R2 | 0,143185 | -0,530902 |
| UB2V1 | 0,726933 | 0,117971 |
| UB2V2 | 0,815062 | -0,0478233 |
| UBA1 | 0,373183 | -0,119595 |
| UBA3 | 0,164737 | 0,969938 |
| UBA5 | 0,590431 | 0,34333 |
| UBA6 | 0,990429 | 0,00309213 |
| UBC12 | 0,790545 | -0,155274 |
| UBC9 | 0,537923 | -0,175824 |
| UBCP1 | 0,879798 | -0,0802704 |
| UBE2H | 0,158541 | 0,7896 |
| UBE2K | 0,0994771 | 0,201449 |
| UBE2N | 0,525528 | -0,176407 |
| UBE2O | 0,321356 | 0,322716 |
| UBE2Z | 0,749198 | -0,109686 |
| UBE3A | 0,285622 | 0,30108 |
| UBE3C | 0,41006 | 0,273191 |
| UBE4B | 0,0224683 | 1,48051 |
| UBF1 | 0,44403 | 0,322786 |
| UBFD1 | 0,997307 | 0,00251929 |
| UBL4A | 0,159522 | 0,931272 |
| UBL7 | 0,292715 | -0,550782 |
| UBP10 | 0,0625901 | 0,418765 |
| UBP14 | 0,000981646 | 0,530173 |
| UBP15 | 0,462629 | 0,203627 |
| UBP19 | 0,600381 | -0,19245 |
| UBP24 | 0,370821 | 0,191326 |
| UBP2L | 0,692609 | 0,0672156 |
| UBP34 | 5,04E-07 | -3,11769 |
| UBP4 | 0,886768 | -0,0142275 |
| UBP47 | 0,94955 | -0,039883 |
| UBP5 | 0,33836 | 0,159665 |
| UBP7 | 0,288267 | -0,279211 |
| UBP8 | 0,757124 | 0,149193 |
| UBQL1 | 0,143049 | 0,376531 |
| UBQL2 | 0,0431102 | 0,692133 |
| UBQL4 | 0,605106 | -0,139505 |
| UBR4 | 0,0019416 | 0,422731 |
| UBXN1 | 0,539409 | 0,196173 |
| UBXN4 | 0,00986107 | 1,1373 |
| UCHL3 | 0,930194 | 0,0300887 |
| UCHL5 | 0,0237669 | 0,397741 |
| UCK1 | 0,00547969 | -1,7554 |
| UCKL1 | 0,437658 | 0,352337 |
| UCRI | 0,40865 | -0,0983089 |
| UD11 | 0,600585 | 0,132921 |
| UD16 | 0,801036 | -0,100236 |
| UD19 | 6,46E-05 | 4,29239 |
| UD2A3 | 0,0250181 | -0,474574 |
| UD3A1 | 0,00531977 | 1,16264 |
| UD3A2 | 0,00398074 | 0,720558 |
| UDB17 | 0,203843 | 0,363171 |
| UFC1 | 0,842762 | -0,0611451 |
| UFD1 | 0,0427105 | 0,319143 |
| UFL1 | 0,0916994 | -0,201496 |
| UFSP2 | 0,431248 | 0,167588 |
| UGDH | 5,87E-07 | 2,33968 |
| UGGG1 | 0,3505 | 0,0780799 |
| UGPA | 3,54E-06 | -1,01718 |
| UH1BL | 0,542119 | -0,455562 |
| UK114 | 6,83E-08 | -2,30306 |
| ULA1 | 0,675569 | -0,0676886 |
| UMPS | 0,374929 | -0,146186 |
| UN45A | 0,962049 | -0,0159327 |
| UPP2 | 0,178091 | -1,19678 |
| UQCC1 | 5,79E-05 | -1,21474 |
| URAD | 0,000206499 | -3,00859 |
| URIC | 0,00261686 | -0,484097 |
| URP2 | 0,0289107 | -0,355425 |
| USE1 | 0,150356 | 0,452689 |
| USMG5 | 0,047981 | -0,639732 |
| USO1 | 0,110557 | 0,181506 |
| USP9X | 0,943491 | 0,00963434 |
| VA0D1 | 0,634218 | 0,0853058 |
| VAC14 | 0,718297 | -0,0720749 |
| VAMP3 | 0,410323 | -0,315865 |
| VAMP7 | 0,780632 | -0,177992 |
| VAMP8 | 0,894231 | -0,136991 |
| VAPA | 0,121477 | 0,359727 |
| VAPB | 0,324046 | 0,14082 |
| VASP | 0,0954078 | 0,72386 |
| VAT1 | 0,799127 | -0,038867 |
| VATA | 0,0373186 | 0,279121 |
| VATB2 | 0,029021 | 0,376895 |
| VATC1 | 0,914669 | -0,025177 |
| VATD | 0,0935967 | 0,416855 |
| VATE1 | 0,373373 | 0,308156 |
| VATF | 0,510581 | 0,124744 |
| VATG1 | 0,578798 | 0,138867 |
| VATH | 0,109465 | 0,510506 |
| VAV2 | 0,290794 | 0,410189 |
| VCAM1 | 0,0452355 | -1,8364 |
| VCIP1 | 0,865052 | -0,0752805 |
| VDAC1 | 0,0372773 | -0,38115 |
| VDAC2 | 0,13559 | -0,279824 |
| VDAC3 | 0,101477 | -0,350271 |
| VIGLN | 0,000549983 | -0,381953 |
| VIME | 0,387577 | -0,136453 |
| VINC | 0,0187351 | -0,176806 |
| VINEX | 0,905041 | 0,02799 |
| VIP2 | 0,792273 | 0,0697489 |
| VKGC | 0,00775901 | -0,920894 |
| VKORL | 0,675135 | -0,394948 |
| VMA5A | 0,254372 | 0,118414 |
| VNN1 | 0,00124685 | 1,96648 |
| VNN3 | 0,345651 | 0,411455 |
| VP13A | 0,0321865 | 1,63531 |
| VP13C | 0,3198 | -0,200001 |
| VP26A | 0,371625 | -0,115251 |
| VP26B | 0,702734 | -0,171036 |
| VP37B | 0,5749 | -0,358276 |
| VPP1 | 0,837657 | 0,148898 |
| VPS16 | 0,920753 | -0,0311394 |
| VPS25 | 0,801754 | -0,368373 |
| VPS28 | 0,940532 | -0,0305265 |
| VPS29 | 0,956462 | -0,0139586 |
| VPS35 | 0,431829 | 0,124328 |
| VPS36 | 0,982437 | -0,00405693 |
| VPS45 | 0,612346 | -0,149877 |
| VPS4A | 0,731594 | -0,243874 |
| VPS4B | 0,521838 | -0,156132 |
| VPS52 | 0,594351 | -0,573133 |
| VRK1 | 0,285384 | 0,688406 |
| VTA1 | 0,933629 | 0,0689246 |
| VTDB | 0,95798 | 0,00436052 |
| VTI1B | 0,415535 | 0,243712 |
| VTNC | 0,0287132 | 0,330939 |
| VWA8 | 0,761418 | -0,0345758 |
| VWF | 0,547315 | -0,308614 |
| WASF2 | 0,786621 | -0,0570024 |
| WASH1 | 0,599297 | 0,21599 |
| WASH7 | 0,528295 | 0,107756 |
| WASL | 0,00568559 | 0,516323 |
| WBP11 | 0,633482 | -0,128941 |
| WBP2 | 0,065253 | 1,47849 |
| WDR1 | 0,790352 | 0,0298882 |
| WDR11 | 0,832285 | -0,137157 |
| WDR12 | 0,597462 | 0,221457 |
| WDR13 | 0,973848 | 0,0189018 |
| WDR18 | 0,28426 | 0,681545 |
| WDR26 | 0,0524828 | -1,00243 |
| WDR33 | 0,372295 | -0,408315 |
| WDR44 | 0,566757 | 0,425969 |
| WDR48 | 0,748854 | 0,103953 |
| WDR5 | 0,108184 | 0,390375 |
| WDR61 | 0,034896 | -1,53946 |
| WDR7 | 0,741116 | -0,147619 |
| WDR91 | 0,957967 | -0,0822035 |
| WIBG | 0,45577 | -0,210775 |
| WIPI2 | 0,938279 | 0,0600348 |
| WNK1 | 0,942363 | -0,0480493 |
| WRIP1 | 0,208278 | -0,456774 |
| XDH | 2,77E-06 | 1,13615 |
| XPO1 | 0,553999 | 0,118723 |
| XPO2 | 0,00284455 | 0,303307 |
| XPO4 | 0,902951 | -0,0659145 |
| XPO5 | 0,67558 | 0,420716 |
| XPO7 | 0,297899 | -0,191912 |
| XPOT | 0,948499 | -0,0110919 |
| XPP1 | 0,0208788 | 0,383506 |
| XPP3 | 0,202667 | -0,325246 |
| XRCC5 | 0,694555 | -0,126803 |
| XRCC6 | 0,571002 | 0,0917794 |
| XRN2 | 0,214927 | 0,448976 |
| XYLB | 0,266 | -0,107258 |
| YAP1 | 0,105942 | 0,31915 |
| YBOX1 | 0,000154661 | 0,531389 |
| YBOX2 | 0,0761776 | 1,05357 |
| YBOX3 | 0,0164091 | 1,22106 |
| YES | 0,644672 | 0,0862783 |
| YIF1B | 0,0345246 | 1,48777 |
| YIPF3 | 0,118828 | -0,45838 |
| YKT6 | 0,0504435 | 0,468649 |
| YMEL1 | 0,054448 | 0,608069 |
| YTHD2 | 0,580883 | 0,567258 |
| YTHD3 | 0,102229 | 0,266692 |
| ZA2G | 0,0522498 | 0,353732 |
| ZADH2 | 0,00488858 | -0,587523 |
| ZBP1 | 0,353047 | 1,13068 |
| ZC11A | 0,15052 | -1,2368 |
| ZC3H4 | 0,946766 | -0,0479539 |
| ZC3HE | 0,284931 | -0,345105 |
| ZC3HF | 0,473435 | 0,247971 |
| ZCCHV | 0,111584 | 0,239074 |
| ZCH18 | 0,340987 | -0,91322 |
| ZER1 | 0,212282 | -0,467878 |
| ZFAN1 | 0,236169 | -0,94882 |
| ZFAN6 | 0,0456945 | 0,872 |
| ZFN2B | 0,0760597 | 0,861509 |
| ZFPL1 | 0,21012 | 0,679285 |
| ZFR | 0,350684 | 0,215914 |
| ZFYV1 | 0,0268459 | 0,53077 |
| ZHX3 | 0,133656 | -0,771638 |
| ZN326 | 0,308536 | 0,206617 |
| ZN598 | 0,987808 | 0,00865237 |
| ZN638 | 0,700902 | -0,220796 |
| ZNFX1 | 0,691494 | -0,0726703 |
| ZNT7 | 0,269771 | 0,840028 |
| ZNT9 | 0,952771 | -0,029925 |
| ZO1 | 0,724992 | -0,0465918 |
| ZO2 | 0,0609107 | 0,285525 |
| ZO3 | 0,000522883 | -1,56149 |
| ZPI | 0,0160731 | 1,08867 |
| ZPR1 | 0,501809 | 0,198185 |
| ZRAB2 | 0,528089 | 0,273222 |
| ZW10 | 0,415094 | -0,534459 |
| ZYX | 0,00252862 | 0,708417 |
| ZZEF1 | 0,0631256 | 0,694208 |
| 1433B | 0,325488 | -0,187278 |
| 1433E | 0,81554 | -0,0369425 |
| 1433F | 0,469638 | 0,23596 |
| 1433G | 0,470505 | 0,213537 |
| 1433T | 0,548573 | 0,130645 |
| 1433Z | 0,000515482 | 0,607007 |
| 2A5A | 0,0865276 | 0,447949 |
| 2A5E | 0,106575 | -0,462191 |
| 2A5G | 0,604581 | 0,280789 |
| 2AAA | 0,12784 | 0,188118 |
| 2AAB | 0,0595042 | 0,95866 |
| 2ABA | 0,197982 | 0,216086 |
| 2ABD | 0,190391 | 0,750376 |
| 3BHS1 | 0,0280923 | 1,27663 |
| 3BHS3 | 0,0581148 | 0,357279 |
| 3BHS7 | 4,78E-05 | 0,562595 |
| 3HAO | 0,0032775 | -0,755524 |
| 3HIDH | 0,0111006 | -0,470665 |
| 4EBP2 | 0,300046 | 0,74931 |
| 4F2 | 0,480037 | -0,214978 |
| 5NT3A | 0,00856068 | 0,501521 |
| 5NTC | 0,124829 | 1,06071 |
| 5NTD | 0,116415 | -0,484504 |
| 6PGD | 0,000166471 | 0,670087 |
| 6PGL | 0,326688 | -0,208317 |
