# Supplementary material for: Hepatocyte specific expression of an oncogenic variant of β-catenin results in lethal metabolic dysfunction in mice
Source: Oncotarget. 2018 Jan 30;9(13):11243–57. doi: 10.18632/oncotarget.24346 (PMC5834276; doi:10.18632/oncotarget.24346)
Supplement: Supplementary file 3 [file oncotarget-09-11243-s003.docx]

| **A** |  |  |
| --- | --- | --- |
| **Protein name** | **p-value** | **log2 Difference** |
| **Fatty acid metabolism** |  |  |
| Fatty acid synthase (FASN) | 1,986E-08 | 1,961E+00 |
| Fatty acid binding protein 5 (FABP5) | 7,472E-07 | -2,095E+00 |
| **B** |  |  |
| **Protein name** | **p-value** | **log2 Difference** |
| **Glycolysis** |  |  |
| Hexokinase-2 (HXK2) | 0,0013713600 | 1,19387 |
| Glucokinase (HXK4) | 0,0000041248 | -3,89407 |
| Ketohexokinase (KHK) | 0,0001008860 | -0,775563 |
| Fructose-2,6-bisphosphatase 1 (PFKL2) | 0,0000000002 | 1,78669 |
| Fructose-bisphosphate aldolase B (ALDOB) | 0,0000000003 | -1,15212 |
| **Glygogenesis** |  |  |
| Hexokinase-2 (HXK2) | 0,0013713600 | 1,19387 |
| Glucokinase (HXK4) | 0,0000041248 | -3,89407 |
| Phosphoglucomutase-2 (PGM2) | 0,0026849200 | -0,700911 |
| UTP--glucose-1-phosphorylase (UGPA) | 0,0000035441 | -1,01718 |
| **Glycogenolysis** |  |  |
| Glycogen phosphorylase, liver form (PYGL) | 0,0000739230 | -0,6634 |
| **Gluconeogenesis** |  |  |
| Phosphoenolpyruvate carboxykinase (PCK) | 0,0001087830 | -0,888425 |
| **C** |  |  |
| **Protein name** | **p-value** | **log2 Difference** |
| **Mitochondrial beta-oxidation** |  |  |
| Acetyl-CoA acetyltransferase, (ACAT1) | 0,0000000480 | -1,42126 |
| Enoyl-CoA hydratase, (ECHM) | 0,0130188000 | -0,630362 |
| 3-ketoacyl-CoA thiolase,(ACAA2) | 0,0003510840 | -0,752967 |
| Propionyl-CoA carboxylase alpha chain, (PCCA) | 0,0000000090 | -1,0939 |
| Propionyl-CoA carboxylase beta chain, (PCCB | 0,0000000017 | -1,1192 |
| **Citric acid cycle** |  |  |
| Citrate synthase, (CS) | 0,0004430840 | 0,935628 |
| Carbamoyl-phosphate synthase (CPS1) | 0,0000000001 | -2,47337 |
| Ornithine carbamoyltransferase (OCT) | 0,0000001475 | -1,58479 |
| Argininosuccinate synthase (ASS1) | 0,0000004695 | -0,783678 |
| Argininosuccinate lyase (ASL) | 0,0000279406 | -1,16169 |
| Arginase-1 OS (ARG1) | 0,0000000000 | -3,90511 |
| **Melanovate Pathway** |  |  |
| Hydroxymethylglutaryl-CoA synthase (HMCS1) | 0,0000000088 | 3,6474 |
| Mevalonate kinase (MVK) | 0,0000028451 | 4,80807 |
| Phosphomevalonate kinase (PMVK) | 0,0007872120 | 2,61775 |
| Diphosphomevalonate decarboxylase (MVD) | 0,0008617640 | 3,85789 |
| Isopentenyl-diphosphate Delta-isomerase 1 (IDI1) | 0,0000003818 | 4,87344 |
| **Oxidative stress respons** |  |  |
| Glutathione peroxidase 3 (GPX3) | 0,0078942800 | -0,733283 |
| Glutathione S-transferase Mu 6 (GSTM6) | 0,0000381850 | 3,08288 |
| Thioredoxin-dependent peroxide reductase (PRDX3) | 0,0006250570 | 1,5128 |
| Selenium-binding protein 1 (SBP1) | 0,0000011654 | -1,00919 |
| **D** |  |  |
| **Protein name** | **p-value** | **log2 Difference** |
| **Peroxisomal proteins** |  |  |
| 3-ketoacyl-CoA thiolase A (ACAA1A) | 0,0000000035 | 1,65322 |
| 3-ketoacyl-CoA thiolase B (ACAA1B) | 0,0000005718 | 1,94708 |
| Peroxisomal acyl-coenzyme A oxidase 1 (ACOX1) | 0,0001547360 | 1,15807 |
| Non-specific lipid-transfer protein (SCP2) | 0,0000150237 | 0,943616 |
| Peroxisomal bifunctional enzyme (ECHP) | 0,0003609580 | 0,815145 |
| Alpha-methylacyl-CoA racemase (AMACR) | 0,0000000248 | 1,04487 |
| Peroxisomal multifunctional enzyme type 2 ( DBP) | 0,0000111700 | 1,11044 |
| Peroxisomal NADH pyrophosphatase NUDT12 (NUDT12) | 0,0002321030 | 0,780938 |
| Alkyldihydroxyacetonephosphate synthase (AGPS) | 0,0000202979 | 1,59006 |
| Phytanoyl-CoA dioxygenase (PHYH) | 0,0000111080 | 1,28124 |
| Lon protease homolog 2 (LONP2) | 0,0011207300 | 1,03008 |
| **E** |  |  |
| **Alternative energy pathways** |  |  |
| **Cahill Cycle** | **p-value** | **log2 Difference** |
| **Protein name** |  |  |
| Alanine aminotransferase (ALAT) | 0,0000682503 | 0,605698 |
| **Glutamine synthesis pathway** |  |  |
| Ornithine aminotransferase, (OAT) | 0,0000000000 | 5,8653 |
| Isoform Glt-1A of Excitatory amino acid transporter 2 (SLC1A2) | 0,0000000002 | 8,19148 |
| **Lactic acid fermentation - Warburg effect** |  |  |
| L-lactate dehydrogenase B chain (LDHB) | 0,0000005990 | 2,72259 |
| Probable D-lactate dehydrogenase (LDHD) | 0,0000026280 | 1,09511 |
